# The sterlet sturgeon genome sequence and the mechanisms of segmental rediploidization

**DOI:** 10.1038/s41559-020-1166-x

**Published:** 2020-03-30

**Authors:** Kang Du, Matthias Stöck, Susanne Kneitz, Christophe Klopp, Joost M. Woltering, Mateus Contar Adolfi, Romain Feron, Dmitry Prokopov, Alexey Makunin, Ilya Kichigin, Cornelia Schmidt, Petra Fischer, Heiner Kuhl, Sven Wuertz, Jörn Gessner, Werner Kloas, Cédric Cabau, Carole Iampietro, Hugues Parrinello, Chad Tomlinson, Laurent Journot, John H. Postlethwait, Ingo Braasch, Vladimir Trifonov, Wesley C. Warren, Axel Meyer, Yann Guiguen, Manfred Schartl

**Affiliations:** 1 Physiological Chemistry, Biocenter, University of Wuerzburg, Wuerzburg, Germany; 2 Developmental Biochemistry, Biocenter, University of Wuerzburg, Wuerzburg, Germany; 3 Leibniz-Institute of Freshwater Ecology and Inland Fisheries, IGB, Berlin, Germany; 4 Plate-forme Bio-informatique Genotoul, Mathématiques et Informatique Appliquées de Toulouse, INRA, Castanet-Tolosan, France; 5 SIGENAE, GenPhySE, Université de Toulouse, INRA, ENVT, Castanet-Tolosan, France; 6 Lehrstuhl für Zoologie und Evolutionsbiologie, Department of Biology, University of Konstanz, Konstanz, Germany; 7 Department of Ecology and Evolution, University of Lausanne, and Swiss Institute of Bioinformatics, Lausanne, Switzerland; 8 Institute of Molecular and Cellular Biology, Siberian Branch of the Russian Academy of Sciences, Novosibirsk State University, Novosibirsk, Russia; 9 INRAE, US 1426, GeT-PlaGe, Genotoul, Castanet-Tolosan, France; 10 Montpellier GenomiX (MGX), c/o Institut de Génomique Fonctionnelle, Montpellier, France; 11 McDonnell Genome Institute, Washington University School of Medicine, St. Louis, MO USA; 12 Institute of Neuroscience, University of Oregon, Eugene, OR USA; 13 Department of Integrative Biology, Michigan State University, East Lansing, MI USA; 14 Bond Life Sciences Center, University of Missouri, Columbia, MO USA; 15 INRA, UR1037 LPGP, Fish Physiology and Genomics, Rennes, France; 16 The Xiphophorus Genetic Stock Center, Department of Chemistry and Biochemistry, Texas State University, San Marcos, TX USA; 17 Hagler Institute for Advanced Study and Department of Biology, Texas A&M University, College Station, TX USA

**Keywords:** Molecular evolution, Ichthyology, Comparative genomics, Genome evolution, Evolutionary genetics

## Abstract

Sturgeons seem to be frozen in time. The archaic characteristics of this ancient fish lineage place it in a key phylogenetic position at the base of the ~30,000 modern teleost fish species. Moreover, sturgeons are notoriously polyploid, providing unique opportunities to investigate the evolution of polyploid genomes. We assembled a high-quality chromosome-level reference genome for the sterlet, *Acipenser ruthenus*. Our analysis revealed a very low protein evolution rate that is at least as slow as in other deep branches of the vertebrate tree, such as that of the coelacanth. We uncovered a whole-genome duplication that occurred in the Jurassic, early in the evolution of the entire sturgeon lineage. Following this polyploidization, the rediploidization of the genome included the loss of whole chromosomes in a segmental deduplication process. While known adaptive processes helped conserve a high degree of structural and functional tetraploidy over more than 180 million years, the reduction of redundancy of the polyploid genome seems to have been remarkably random.

## Abstract (web summary)

A genome assembly of the sterlet, *Acipenser ruthenus*, reveals a whole-genome duplication early in the evolution of the entire sturgeon lineage and provides details about the rediploidization of the genome.

## Main

Vertebrate genome evolution has been strongly impacted by polyploidization events^1,2^. Early on, vertebrate ancestors experienced two rounds (1R and 2R) of whole-genome duplications (WGDs)^3^. The evolutionary history of the ~30,000 species of teleost fish, which make up more than 99% of all ray-finned fishes (Actinopterygia), is defined by a third WGD (3R) that occurred in their common ancestor about 320 million years ago (Ma), but not in the basal fish (bichirs, reedfish, sturgeons, paddlefishes, bowfins and gars), the land vertebrates or their sarcopterygian forbearing relatives (coelacanths and lungfishes). Some teleost groups, such as salmonids and carps, independently underwent another round (4R) of WGD. Interestingly, among the basal fishes only the sturgeon lineage is known to be prone to polyploidization events and includes many-ploid species, some with up to 380 chromosomes.

Sturgeon genomes, however, are a missing puzzle piece for understanding vertebrate ancestry. Sturgeons are a group of ray-finned fish that diverged from the actinopterygian stem before the teleost-specific 3R duplication and after the ancient 2R event^4,5^. After their divergence from the other ray-finned fish, the various lineages of Acipenseriformes (sturgeon and paddlefish) experienced several polyploidization events^6^, resulting in karyotypes, comprising between **~**120 chromosomes in some species, and **~**360 chromosomes in species that are considered dodecaploid^7^. The genomic basis for this parallelism between basal and derived fish lineages to acquire WGDs is not clear. While teleost lineages that experienced more recent 4R events are still recognizable apparent tetraploids, the other teleost lineages retained on average only 17% of gene duplicates from the ancient 3R ohnologues^5^. The evolutionary trajectories and forces driving species from polyploids to meiotic diploids are the subject of major adaptive hypotheses and their empirical evaluations^8,9^.

The genomic state of sturgeons is much less clear. They are often seen as ancient polyploids. On the basis of some cytogenetic and microsatellite data, others have considered sturgeons to be functional diploids^10^ as result of an evolutionary process, where the gene content of a tetraploid species degenerates to become functionally diploid but maintains twice as many chromosomes, which form regular bivalents^11^. Such far-reaching redundancy reduction leads one to question their polyploidy state^12^.

Because sturgeons branched off early from modern fishes, their genomes may harbour traces of the ancient vertebrate ancestors^13^. Notably, their early embryonic development is of the classical amphibian type and very different from that of all modern fish^14,15^, reflecting the basal divergence of the lineage.

Sturgeons are distributed from subtropical to subarctic rivers, lakes and coastlines of Eurasia and North America^16^. They are long-lived and reproduce late, usually not before reaching an age of ten years. In many sturgeon species, adults migrate repeatedly from the sea into freshwater to spawn^17^. Sturgeons are celebrities among fishes because of their pre-ovulation female gametes, known as caviar. Habitat destruction, the lack of river connectivity, pollution^16,18^ and the 2,000-year-old rural caviar production^19^ culminated in ongoing devastating overexploitation that drove most sturgeon species into a threatened status (https://www.iucnredlist.org/). Because wild caviar can no longer be traded legally, sturgeon aquaculture has gained high economic importance, and in turn can contribute to the protection of wild populations by providing a safe market supply.

Despite their ancient lineage, peculiar biological features and economic value, sturgeon genomes have remained largely unexplored owing to their dauntingly polyploid state^20^. We therefore sequenced the sterlet sturgeon, *Acipenser ruthenus*, a species with only 120 chromosomes, and present here an annotated chromosome-scale genome assembly. We found that this genome represents an ancient WGD, which remained close to tetraploidy owing to the slow evolutionary rate and serves as a good representative of the ancestral actinopterygian genome. In contrast to other polyploid fish, deduplication after the sterlet WGD involves the loss of entire homeologous chromosomes (segmental rediploidization). Adaptive processes in the retention of duplicate genes are only partly responsible for determining the gene content, and they worked in parallel with stochastic events to shape the genomic landscape of the tetraploid sterlet sturgeon.

## Results

### Genome assembly and annotation

Polyploid genomes are extremely challenging for de novo assembly because of the coexistence of ohnologous and allelic sequences of each original locus with various degrees of sequence similarities. To generate a high-quality reference sturgeon genome, we produced 42-fold coverage of Illumina sequences, 54-fold coverage with PacBio long reads and 20-fold coverage of Hi-C sequences of the estimated 1.8-gigabase (Gb) genome of a male *A. ruthenus*^21^. For the assembly process, we considered possible complications owing to the simultaneous presence of polyploidy and heterozygosity (Supplementary Note 1). After reduplication and Hi-C scaffolding, we produced a 1.8-Gb assembly with a final N50 scaffold size of 42.4 megabases (Mb) (Supplementary Fig. 1, and Supplementary Tables 1 and 2). The 60 largest scaffolds correspond to 120 chromosomes of the sterlet karyotype. The chromosome number of *A. ruthenus* can vary, however, by two to four small chromosomes, indicating the occurrence of B chromosomes^22^. B chromosomes are enigmatic accessory elements to the regular chromosome set. They are found in some but not all individuals within a population and are considered to be either non-functional, beneficial or harmful^23^. Scaffold 60 consists mainly of interspersed repetitive DNA (83.9%) and contains only three corrupted gene remnants, thus probably representing a fully assembled B chromosome (Supplementary Table 2 and Supplementary Note 2).

Genome annotation combined gene evidence from homology annotation, de novo annotation and transcripts with a previously established pipeline^24^. We predicted 47,424 protein-coding genes. BUSCO analysis revealed that the annotation contains 2,543 (98.3%) out of 2,586 conserved and complete vertebrate genes (Supplementary Table 3).

### Ancient origin and slow evolution

**Fig. 1:**
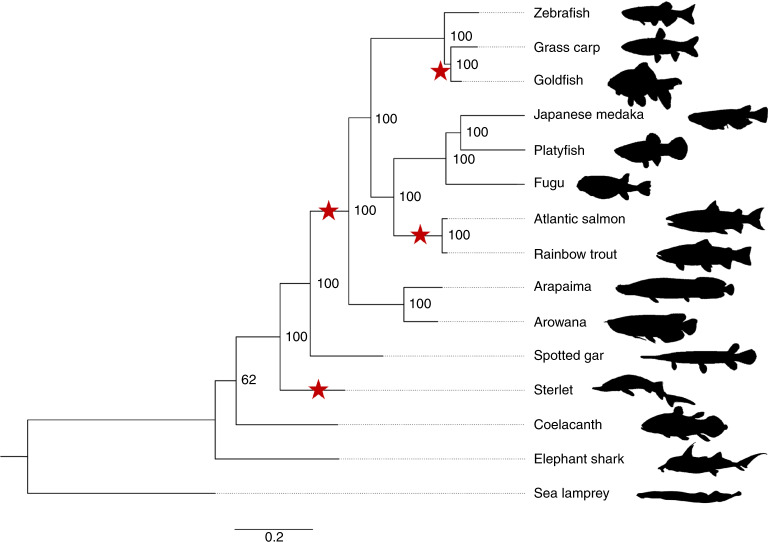
Phylogeny of sterlet and related species. Species tree built using RAxML on the basis of 47 one-to-one orthologues. The sea lamprey was used as the outgroup. The topology of the tree was confirmed by MrBayes (see also Supplementary Fig. 2). Red stars indicate WGDs after the 1R/2R event; numbers at branches indicate bootstrap support values based on 100 resampled data sets; the scale bar indicates the average substitutions per site; the dotted lines associate the taxon names with the branch ends.

Sturgeons are one of the most deeply diverging groups of bony fishes and have been referred to as both the Leviathans and Methuselahs of freshwater fish. They appear in the fossil record between 250 and 200 Ma, near the end of the Triassic. Our phylogenomic trees place the sterlet sturgeon basal to the other ray-finned fishes (Fig. 1 and Supplementary Fig. 2), in agreement with the current tree of life^25–28^. Divergence time inference based on 275 one-to-one orthologues revealed that the sterlet lineage had already diverged from the actinopterygian fish 345 (295–400) Ma during the Upper Devonian or Carboniferous period (Supplementary Fig. 3), in the range of earlier estimates^28^.

Because extant sturgeons show remarkably little morphological change compared with fossils from the Triassic and because most of the 27 extant species differ relatively little except in body size^29^, Charles Darwin called them living fossils^30^. We therefore asked whether the morphological stasis in sturgeons is matched by a slowly evolving genome as inferred from the slower substitution rates of several mitochondrial and nuclear genes^31^. Calculations of pairwise distances from phylogenetic trees (Supplementary Table 4) revealed that proteins in sterlet are indeed evolving much more slowly than in teleosts, including basal species such as arowana and arapaima. The rate of protein evolution is even slower than in gar, and similar to those basal lineages such as coelacanth or elephant shark (Fig. 1, Supplementary Tables 4–6 and Supplementary Note 3).

The repeat content (40.3%) and transposable element (TE) composition (Supplementary Table 7) of the sterlet genome are comparable to those in other fish (teleosts, gar, elephant shark and coelacanth) studied so far^32^. Despite representing an old, slowly evolving lineage, the inferred transposon activity revealed a recent expansion of all major types of TEs (Supplementary Fig. 4a). The presence of TEs in sterlet transcriptomes, in particular of endogenous retrovirus long terminal repeat (EVR-LTR) retrotransposons and transfer-RNA short interspersed nuclear elements (tRNA-SINEs), indicates that the sterlet retains some active transposons (Supplementary Fig. 4b). The mobilome of the sterlet sturgeon thus seems to be similar to that of many modern fish genomes, including fast-evolving teleosts. This situation contrasts notably with the slow evolution of sterlet protein-coding genes, but recently expanding TEs and slow protein evolution also occur in the coelacanth genome^33^.

### The sterlet WGD and its initial rediploidization

Cytogenetic and microsatellite data supported the notion that polyploidy is a general feature of sturgeons. We identified 11,765 genes that have two copies in sterlet but only a single-copy orthologue in gar, coelacanth or elephant shark. We further identified in sterlet 9,914 high-fidelity ohnologue pairs with positional orthology (Supplementary Table 8). A comparison with gar revealed double conserved synteny for 8,752 genes (Supplementary Table 9). This all indicates a WGD in the sterlet lineage (Ars3R) (Supplementary Fig. 5).

To estimate the timing of the Ars3R event, we calculated the pairwise synonymous substitutions per synonymous site (dS) value among sterlet ohnologue pairs (median, 0.064) and between sterlet and one-to-one orthologues of five other sturgeon species (http://publicsturgeon.sigenae.org/home.html) (Supplementary Note 4). On the basis of our timing of the sterlet–Atlantic sturgeon (*A. oxyrinchus*) divergence at 166 (115–208) Ma (Supplementary Fig. 6a) and the dS value between their orthologous pairs (median, 0.059; Supplementary Fig. 6b and Supplementary Table 10), we deduced that the sterlet WGD must have happened around 180 (124–225) Ma. Thus, the Ars3R genome duplication event is older than the salmonid WGD at 80–100 Ma (refs. ^34,35^) and the carp–goldfish 4R estimated at 14 Ma (ref. ^36^).

**Fig. 2:**
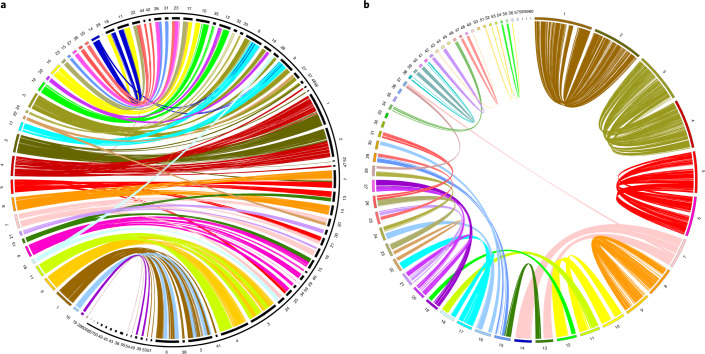
Homology and homeology relationships of sterlet chromosomes. **a**, Chord diagram displaying the gene orthologies between 29 spotted gar chromosomes (left, coloured) and 60 sterlet chromosomes (right, black, bracketed by outer black partial circle) on the basis of 21,085 orthologous pairs (pairwise synteny was confirmed by the criterion of at least four orthologous genes, arranged in a row with the largest gap being fewer than 15 genes). **b**, Chord diagram depicting homeology relationships of 60 sterlet chromosomes on the basis of 9,301 ohnologue pairs (pairwise synteny was confirmed by the criterion of at least five ohnologues, arranged in a row with the largest gap being fewer than 15 genes). The chromosomes are ordered by size.

The analysis of conserved syntenies between sterlet and gar revealed that most gar chromosomes have two counterparts in sterlet (Fig. 2a). When sterlet ohnologous gene pairs were mapped against the genome scaffolds, they delineated 46 scaffolds, also in a pairwise fashion. This result indicates homeologous chromosome segments, as expected from a WGD event (Fig. 2b, Supplementary Figs. 7 and 8, and Supplementary Notes 5 and 6). To confirm this conclusion, we used sequence libraries, prepared from individual microdissected chromosomes or chromosome arms of the sterlet^37,38^. In whole-mount in situ-hybridizations, each of these probes painted two pairs of sterlet metaphase chromosomes and chromosome arms, respectively, identifying likely ohnologous pairs. Reads from each of the libraries aligned specifically to individual scaffolds, which thereby could be assigned to either of the homeologous chromosome segments (Supplementary Figs. 9 and 10, Supplementary Note 6 and Supplementary Data 1).

Remarkably, most of the large homeologous chromosomes (1–6, 8 and 9) are conserved over their full length, while the majority of the intermediate-sized chromosomes have ohnology-relationships to two other chromosomes. The alignment of chromosomes by LAST indicated that whole chromosome arms were exchanged, most probably in reciprocal translocation events (Supplementary Figs. 8 and 11, and Supplementary Note 5).

Interestingly, the remaining 11 scaffolds, corresponding to smaller chromosomes, contain exclusively singletons or only a small region with ohnologues on another chromosome, while the remainder of the chromosome only contains singletons. Those small ohnologue regions are obviously translocations from other chromosomes (Supplementary Fig. 12a). We conclude that the entire homeologue or the majority region of the counterparts of those smaller, whole-chromosome-representing scaffolds, were lost after the Ars3R (Supplementary Fig. 13). This result indicates that a relevant part of the deduplication process in sterlet occurred by the loss of whole chromosomes or large chromosome fragments and is segmental. This mechanistic conclusion is in contrast to the continuous and genome-wide small-scale ohnologue-by-ohnologue loss in carp/goldfish and salmonids (Supplementary Fig. 12b–d). Earlier molecular cytogenetic studies of sterlet also pointed to a karyotype that is segmental rather than ubiquitously polyploid^38^. Such large-scale reduction of duplicates in polyploid organisms, through the loss of whole chromosomes or large chromosome segments, has so far been reported only in autotetraploid yeasts^39,40^, flowering plants^41^ and endopolyploid human cancer cells^42^.

**Fig. 3:**
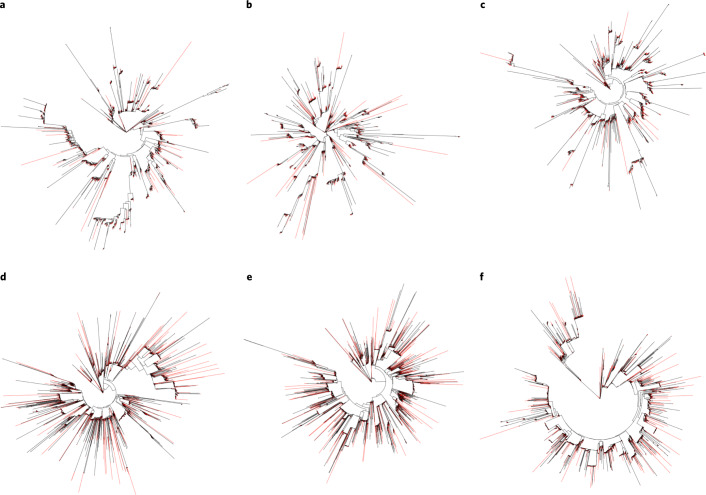
Phylogeny of DNA/PIF-Harbinger and DNA/TcMar-Tc1 repeat families on homologous chromosomes. **a**, DNA/PIF-Harbinger on homologous chromosomes 1 (red) and 2 (black). **b**, DNA/PIF-Harbinger on homologous chromosomes 3 (red) and 4 (black). **c**, DNA/PIF-Harbinger on homologous chromosomes 5 (red) and 6 (black). **d**, DNA/TcMar-Tc1 on homologous chromosomes 1 (red) and 2 (black). **e**, DNA/TcMar-Tc1 on homologous chromosomes 3 (red) and 4 (black). **f**, DNA/TcMar-Tc1 on homologous chromosomes 5 (red) and 6 (black).

Polyploidy can result from duplication of the whole genome in one organism (autopolyploidy) or from the interbreeding of two divergent species with subsequent genome doubling that restores meiotic pairing and disomic inheritance (allopolyploidy). Both of these mechanisms—interspecific hybridization and autopolyploidization—have been discussed to account for the origin of the sterlet chromosome complement, on the basis of conflicting evidence^12^. To clarify this controversy, we used a strategy that was employed to investigate this problem in the allopolyploid African clawed frog, *Xenopus laevis*, where the fast-evolving repeats and relics of the mobilome are specific to the allopolyploid ancestors, and thus markers for the ancestral chromosomal segments of the two parental species^43^. A comparison of the TE landscape of sterlet paralogous chromosomes revealed that each pair has an almost identical TE content and that individual TE families are monophyletic (Fig. 3 and Supplementary Fig. 14). The sterlet genome thus shows no evidence for allopolyploidy.

Chromosomes that have retained a homeologous partner share to a large extent even their gene order (Supplementary Figs. 7 and 8). This phenomenon has also been observed in many polyploid plant species and is called positional orthology^44,45^. It is explained as a consequence of multivalent pairing in meiosis. Multivalent pairing would also explain tetrasomic inheritance in sterlet, noted earlier from microsatellite studies^12^.

The duplication of a whole genome creates a situation, where one of the two copies is in principle dispensable. The retention of duplicates is explained by several models^46^. They may be preserved if one copy evolves a new positively selected function and simultaneously loses the essential function retained by the other copy (neofunctionalization) or if ancestral positively selected functions partition between the two copies (subfunctionalization)^9^. The gene balance hypothesis posits that ohnologues persist because the loss of one copy would lead to a detrimental change in the stoichiometry of macromolecular complexes, the interactome and signalling pathways^47^. The majority of duplicates, however, are predicted to become non-functional or get lost (degeneration)—for example, the ohnologue retention rate from the teleost WGD in the extant teleosts is estimated to be only 15–20%^48^. On the basis of non-coding microsatellites, the sterlet was proposed to have undergone extensive duplicate gene degeneration and has been classified since then even as a functional diploid species^10^. To estimate the duplicate retention rate, we identified 9,914 high-fidelity pairs of ohnologues and 4,175 singletons (Supplementary Note 7). This dataset represents a duplicate retention rate of 70% (Supplementary Table 11), considerably higher than in all teleosts, including the 4R salmonids (Supplementary Note 8). Considering functional terms, we found that sterlet ohnologues are enriched for transcriptional regulators (genes involved in protein turnover, signal transduction, cell proliferation and development), in agreement with predictions from the gene-balance-hypothesis^47^. Sterlet singletons are enriched for genes with functions in DNA metabolism, intracellular transport and mitochondria. Enrichment for such categories has been observed in other polyploids, even in plants^49–51^ (Supplementary Table 12). Like the situation reported for rainbow trout^34^, we found the coding sequence of singletons to be significantly shorter than that of ohnologues (12%, *P* < 2.2 × 10^−16^; Supplementary Fig. 15). Long genes may be over-retained as ohnologues, potentially owing to more opportunities for protein domain subfunctionalization.

In our analysis of transcriptomes from 23 different sterlet organs and developmental stages, we observed the expression of one or both genes for 9,243 of the 9,914 ohnologue pairs. We found 1,139 ohnologue pairs, which showed equal expression in all samples (Supplementary Fig. 16a). We then searched for genes with differing expression patterns among samples, which would be explained by drift models of expression change or would indicate the degeneration or neofunctionalization of one duplicate, or subfunctionalization of both copies. We found 3,230 ohnologue pairs with different expression in at least two samples (Supplementary Fig. 16b and Supplementary Note 8). From just 38 of these ohnologue pairs, only one of them was expressed but never the other in all organs tested. Such a pattern is expected if regulatory elements are degenerating in the redundant copy. For 341 ohnologue pairs, the expression of duplicates was partitioned between different organs or developmental stages. This may indicate subfunctionalization of this subset of genes.

The availability of the sterlet genome now allows the revisitation of important questions concerning the forces that affect the evolutionary fate of gene duplicates. We compared the genomes of sterlet, salmon, trout, goldfish and zebrafish, using gar as the outgroup, to find genes that were commonly retained in duplicate after the various polyploidization events (Supplementary Note 9). We found only 27 such genes (Supplementary Figs. 17a and 18, and Supplementary Table 13). This finding suggests complex, independent, lineage-specific evolutionary processes of duplicate retention.

In the same set of species, we identified 191 genes that are singletons in all of them (Supplementary Note 9). Notably, 39 of these singletons are arranged in eight syntenic blocks. A similar phenomenon was seen for the commonly retained ohnologues (Supplementary Figs. 17b and 19, and Supplementary Table 14). The loss or retention of linked genes after WGDs could be explained by the functional relationships of their gene products—for example, through protein–protein interactions^52^. However, a search of singleton genes, embedded in syntenic blocks using the STRING^53^ database, did not reveal such protein–protein interactions. An alternative explanation for the conservation of microsynteny is the bystander relationship^54^, where the regulatory region of one gene is located in neighbouring genes. Further studies are required to validate this type of physical association of genes on chromosomes over long evolutionary times rather than functional relationships of their encoded proteins.

### Genome and gene evolution

#### Positive selection

Up to 210 genes (Supplementary Table 15) in sterlet are under positive selection, depending on the set of actinopterygian or vertebrate genomes, with which its full gene complement was compared (Supplementary Table 16). Positively selected genes spanned a wide spectrum of cellular and molecular functions and pathways with no particular enrichment.

When the ratios of substitution rates at non-synonymous versus synonymous sites (dN/dS values) were compared between sterlet singletons and ohnologues, we found that most retained ohnologues present higher dN/dS values than singletons (Supplementary Fig. 20), indicating relaxed purifying selection on ohnologues. This result would be expected because of ohnologue redundancy^55,56^. A pairwise test of dN/dS for the 9,914 ohnologue pairs revealed that 207 are under positive selection in sterlet, pointing to neofunctionalization or subfunctionalization at the protein level (Supplementary Table 17). Notably, many immune-related genes are positively selected, indicating that the sterlet host defence system may have made have especially profited from the WGD for evolutionary progress sensu Susumo Ohno^57^. A similar phenomenon was observed for duplicated immune genes in salmon^58^.

#### Dynamics of gene family size

We compared the rates of gene family (8,150 gene families) dynamics between phylogenetic tree branches with different WGD histories and found that gene family sizes changed much faster in branches with 4R and Ars3R than in branches with more ancient polyploidization (Supplementary Note 10). Interestingly, one of the most expanding families is the *zona pellucida* (Zp) sperm-binding proteins (ID: 4190). Zp-proteins prevent polyspermy in mammals^59^ and provide thickness and hardness to the fish egg envelope^60^. A total of 116 *zp* genes were annotated in sterlet (Supplementary Table 18 and Supplementary Note 11). A similar expansion was noted in cold-adapted teleosts and explained as a protection mechanism from physical forces for the developing embryo^61,62^. The biological reason for the *zp* gene family expansion in sturgeon is unclear. Because sturgeons spawn on a coarse substrate often in high current velocities, a hard envelope provides protection against mechanical stress of the adhesive eggs on the spawning substrate as well as against polyspermy that would be possible through the multiple micropyles of their eggs. This biological feature might contribute to the crispness of the caviar.

#### Evolution of sterlet *hox* clusters after genome tetraploidization and inference of the ancestral vertebrate *Hox* complement

**Fig. 4:**
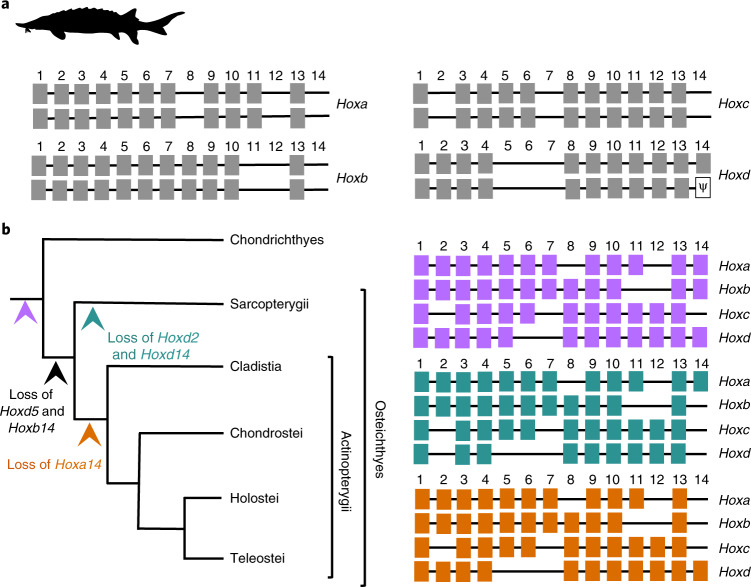
Structure and evolution of *hox* clusters. **a**, Schematic illustration of the sterlet *hox* complement. We identified 88 *hox* genes plus one pseudogenized *hoxd14* gene (indicated by psi). All *hox* clusters are retained in duplicate. **b**, Reconstruction of the ancestral actinopterygian condition and the inference of gene losses across the gnathostome phylogeny on the basis of the sterlet pretetraploidization *hox* complement in combination with that of the gar. The inferred ancestral *Hox* complements are shown in purple (likewise indicated by the purple arrowhead in the tree) for gnathostomes, in blue for Sarcopterygii and in orange for Actinopterygii.

The sterlet has eight *hox* clusters containing 88 genes, reflecting the 1R/2R/3R history of its genome (Fig. 4a and Supplementary Note 12). Pseudogenization was apparent for only one *hoxd14* gene. The sterlet therefore retains the most complete 3R *hox* cluster duplicates and the highest number of 3R *hox* gene ohnologues amongst ray-finned fish. The comparison of the *hoxd* flanking gene deserts, containing long-range regulatory elements^63–66^, indicates high conservation of ultraconserved elements (Supplementary Fig. 21). The preservation of all duplicated *hox* clusters as well as their low divergence, including that of their regulatory regions, shows a remarkable slow evolution of these genomic loci. This stability contrasts sharply with rapidly evolving teleosts, which often show extensive remodelling of duplicated *hox* clusters^4,67–73^.

The *hox* gene complement in sterlets indicates an identical pre-tetraploidization *hox* gene arrangement and repertoire with the gar (diverging ~345 Ma). Because both species represent early-branching ray-finned fish, this similarity strengthens the scenario whereby *hoxd5* and *hoxb14* were lost in the common ancestor of bony vertebrates (Euteleostomi) and *hoxa14* in the common ancestor of actinopterygians^66^ (Fig. 4b).

#### Over-retention of glutamate receptor genes

Glutamate receptor genes (GRGs) show particularly high ohnologue retention rates in teleosts^74^, which has been connected to the extraordinary cognitive abilities of many teleost species compared with other basal vertebrates. We found that 23 of 26 GRGs retained their Ars3R ohnologue, an ohnologue retention rate of 88.5% (Supplementary Fig. 22, Supplementary Table 19 and Supplementary Note 13). Compared with the genome-wide rate of 70% (9,914 ohnologs and 4,175 As3R singletons), the GRG Ars3R ohnologue retention rate is significantly higher (*P* = 0.04345, chi-square test). GRGs have thus been convergently over-retained, following the Ars3R and teleost 3R WGD, although to a lower extent in sturgeons.

#### Absence of differentiated sex chromosomes

The relative rarity of polyploidy in animals versus plants has been ascribed to the disruption of sex determination in gonochoristic animals after genome duplication^75–77^. Differentiated sex chromosome pairs have largely different gene contents, to which many animals have adjusted by elaborate expression dosage compensation mechanisms. The disturbance of dosage compensation and the disruption of the chromosomal system that determines the sex ratio are thus immediate negative consequences of polyploidization^78^. Data from induced gynogenesis led to the common belief that all *Acipenser* species, including sterlet, have a female heterogametic (ZZ/ZW) sex chromosome system^79,80^. To find out if the polyploid sterlet has differentiated sex chromosomes, we searched for sex-linked sequence differences using a restriction site associated DNA (RAD) sequencing approach. A total of 176,735 markers were obtained, but none showed a bias or specificity for males or females (Supplementary Fig. 23). This result indicates that the sterlet does not have sex chromosomes with considerable sequence differentiation that would require dosage compensation and impair the occurrence of polyploidy. Our data are in agreement with the absence of differences in chromosome morphology and previous failures to isolate sex-specific molecular markers^81^.

## Discussion

The high-quality chromosome-level genome of the sterlet sturgeon permitted important advances in our understanding of the evolution of this lineage of ancient fish. Our results show that the sterlet lineage branched from the vertebrate tree of life about 345 Ma, shortly after the basal split between the linage of ray-finned fish and that of lungfish, coelacanth and land vertebrates happened. While the sterlet’s slow evolutionary rate of protein-coding genes is not entirely unexpected, given the morphological stasis exhibited in the sturgeon lineage, many of the features of the sterlet’s polyploid genome are much different from those of other polyploid lineages. Clearly, genomic and phenotypic evolution do not always march to the beat of the same drummer.

All sturgeons are characterized by polyploidy as a genetic hallmark and paramount feature. It has been proposed that those extant sturgeons with ~120 chromosomes (like the sterlet) represent functional diploids, which originated over 200 Ma by a WGD of a 60-chromosome diploid ancestor^82^. The transition between the ancestral fully tetraploid and the modern functional diploids was proposed to have been accompanied by a reduction of duplicate gene functions^12^. Our estimate of 180 Ma for the Ars3R provides evidence for a WGD in the ancestor of all sturgeons, and that the WGDs that led to the ~240- and ~360-chromosome species happened later, on top of the Ars3R. We found that despite the long evolutionary time that has elapsed since the sturgeon WGD, the sterlet has not returned to a diploid state by gene content or gene expression. Instead, the sterlet has retained an unexpectedly high degree of structural and functional polyploidy. This retention can be ascribed to the slow pace of molecular evolution of most fractions of the sterlet genome.

The slow evolution may also explain why the sterlet genome in several aspects represents an earlier step in the process of redundancy-reduction than the salmonid genomes, which originated from a more recent WGD. During the evolution of a polyploid genome, the initial one-to-one relationship of whole chromosomes (as still seen in the goldfish) is reduced to homeology between arms of chromosomes and then further to much smaller regions (as evident in salmonids). Sterlet seems to be in the transition towards the highly dynamic pattern of colinear duplicated blocks, but still has some fully homeologous chromosomes (Supplementary Fig. 24).

A recent wave of TE multiplication apparently swept through the sterlet genome after the Ars3R. The large-scale expansion and movement of TEs are known to increase under genomic stress^83^, suggesting that WGDs cause TE activation. TE expansions in the centromere induce chromosomal instability^84^ and might have facilitated the large chromosome rearrangements of homeologue arm changes.

The timing of the Ars3R to have evolved earlier than the cyprinid and salmonid 4Rs allows comparisons of the three apparent tetraploid lineages to give insights into the processes of polyploid genome evolution. Despite its apparent evolutionary advantage as a source of genomic matter for evolution in the long term, tetraploidy seems to be an evolutionarily unstable situation. In all known instances, the initial dispensability of two sets of genes led to deduplication of the genome, with only a certain fraction of gene duplicates being retained.

The process of duplicate gene loss after the teleost, salmonid and goldfish WGDs affected the whole genome in a homogenous fashion. Unexpectedly, the sterlet genome analysis uncovered a phenomenon that creates a segmental rather than a continuous partial tetraploidy. In the sterlet, most chromosomes or chromosome arms were found to be in either a diploid or a tetraploid state. The loss of entire chromosomes can be seen as a fast stochastic process for rediploidization.

The numbers of genes that were either commonly retained or deduplicated after the WGDs in the fish lineages are substantially above random but are much lower than one would expect if strong adaptive processes determined duplicate retention or loss on the single-gene level. This conclusion, and our finding that structural features rather than protein–protein interactions are relevant for the deduplication of neighbouring genes, suggest complex processes of different lineage-specific evolutionary drivers of duplicate retention, and largely stochastic events in redundancy reduction. In sterlet, besides the adaptive evolutionary mechanisms, neutral processes have considerably shaped its genome, most obviously manifested by the loss of whole chromosomes from homeologues pairs.

## Methods

### Experimental animals

All fish used in this study were derived from the sterlet sturgeon population maintained at the Leibniz-Institute of Freshwater Ecology and Inland Fisheries. This stock is derived from the Danube population of *A. ruthenus*. Adult individuals were sexed by gonad morphology and gamete content. The fish were euthanized by state-of-the-art humane killing (American Veterinary Medical Association, Canadian Council of Animal Care in Science). The experiments were carried out in accordance with the European Directive 2010/63/EU and German national legislation (animal protection law, TierSchG). All experimental protocols that are part of this study were approved through an authorization (File No. ZH 114, issued 6 February 2014) of the LAGeSo, Berlin, Germany.

### Genome sequencing and assembly

The DNA for sequencing was derived from the testis and blood of a single adult male. We generated ×42 Illumina reads (150-base-pair (bp) paired end) on a Novaseq 6000 platform with libraries produced using the TruSeqDNA PCR-Free kit. A 53.7-fold coverage of genome sequences was produced with PacBio Sequel technology. Hi-C library generation was carried out according to a protocol adapted from Foissac et al.^85^. A blood sample was spun down, and the cell pellet was resuspended and fixed in 1% formaldehyde. Five million cells were processed for the Hi-C library. After overnight digestion with HindIII (NEB), the DNA ends were labelled with Biotin-14-DCTP (Invitrogen), using Klenow enzyme (NEB), and then religated. Next, 1.4 µg of DNA were sheared by sonication (Covaris) to an average size of 550 bp. Biotinylated DNA fragments were pulled down using M280 Streptavidin Dynabeads (Invitrogen) and ligated to paired-end adaptors (Illumina). The Hi-C library was amplified using paired-end primers (Illumina) with 10 PCR amplification cycles. The library was sequenced using HiSeq3000 (Illumina) generating 150-bp paired-end reads at 20-fold genome coverage.

The raw Sequel BAM files were converted into subreads in fasta format using the SMRT Link software package (v.5.0.1) from Pacific Biosciences^86^. PacBio reads were assembled with smartdenovo (v.1.0)^87^ with standard parameters. Contigs were polished with two rounds of racon^88^ (v.1.3.1), using long reads aligned with minimap2 (ref. ^89^) (v.2.7) and three rounds of pilon^90^ (v.1.22), using 42-fold Illumina reads. The Illumina reads were aligned with bwa mem (v.0.7.12-r1039)^91^ with standard parameters and the same file, which had been compressed, sorted and indexed with samtools view, sort and index v.1.3.1^92^, using standard parameters before pilon polishing. The genome size was 15% smaller than expected, and a fraction of the contigs showed twice the expected read alignment depth, indicating that chromosome parts had merged during assembly. The single- and double-copy coverage threshold was found by visual inspection of the contig coverage bimodal distribution, and the contigs were separated into two sets, corresponding to single and double coverage. A polymorphism VCF file was generated from the short read alignment file with freebayes^93^ (v.1.1.0) under standard parameters. The VCF file shows an overall much higher variation density in double coverage contigs. PacBio long reads were used in the next steps to generate haplotypes of these variations to split the genomic locations that had been merged. Long reads were aligned to contigs, and the alignments of double coverage contigs were processed with HapCut2^94^ (v.1.0) using the following parameters: extractHAIRS –ont 1 and HAPCUT2 –ea 1. For each contig, a haplotyped VCF file was produced. Some of these files contained more than one haplotypic segment. These contigs have been split according to the haplotypic segment information found in the VCF file, using an in-house script. The resulting haplotyped VCF files were then processed with fgbio (v.0.7.0 using standard parameters)^95^ to generate VCF files, separated by haplotype. These VCF files and the reference were used to produce haplotypic contigs using vcf-consensus from the bcftools^96^ package v.1.8 under standard parameters. Both contig sets, unique and split, were then merged using the Unix cat command. The Hi-C short reads were aligned to the contigs with Juicer^97^, and the scaffolding was performed with 3D-DNA^98^ with parameter -r = 0. Finally, the candidate assembly was manually reviewed using the Juicebox Assembly Tools^99^. The contig metrics were calculated with the assemblathon_stats.pl script.

### Repeat annotation and TE analysis

To search for repeated elements, the sterlet genome and raw Illumina reads were used as input. The assembled genome was used in the RepeatModeler open-1.0.11 tool^100^ with standard settings. LTR-retriever v.2.5 (ref. ^101^) was used to search for full-length LTR elements, and the data were used as input derived from the LTRharvest^102^ (-similar, 90; -vic, 10; -seed, 20; -seqids, yes; -minlenltr, 100; -maxlenltr, 7,000; -mintsd, 4; -maxtsd, 6; -motifmis, 1) and LTR_FINDER^103^ (-D, 15,000; -d, 1,000; -L, 7,000; -l, 100; -p, 20; -CM, 0.9) tools. To exclude non-LTR (-linelib) and DNA transposons (-dnalib), protein sequences of these TEs from the RepeatPeps database of the RepeatMasker tool^104^ were used. This also excluded protein sequences that were not related to TEs. The SWISS-PROT sequence library^105^ was also used (-plantprotlib).

The sequences obtained using the previous steps were combined into a single FASTA file using CD-HIT-est^106^ (-aS, 1; -c, 1; -r, 1; g, 1; p, 0). The resulting FASTA file was aligned against the RepBase v.24.07 (ref. ^107^) and FishTEDb^108^ databases using blastn (-evalue, 10 × 10^−100^) and against SWISS-PROT and RepeatPeps using blastx (-evalue, 10 × 10^−100^)^109^ to filter incorrectly annotated sequences.

Raw reads were used in the TAREAN tool^110^, which is part of RepeatExplorer^111^. The reads were first trimmed using the fastp tool^112^ to remove low-quality and adapter sequences (detect_adapter_for_pe -g -c -l 50 -5 -3), after which RepeatExplorer was used with standard settings. We saved only satellite sequences with high confidence and added them to the library of repeated sequences. In addition, using REXdb^113^, a database of TE domains implemented in RepeatExplorer2, the correctness of the previous TE annotation was further verified. The content of repeated elements in the genome was estimated using RepeatMasker open-4-0-9-p2 (-s −no_low −lib). To build the Kimura plot, the createRepeatLandscape.pl script from the RepeatMasker tool was used.

To analyse the expression of TEs, raw reads from RNA-seq were used. The reads were trimmed using fastp (–detect_adapter_for_pe -g -c -5 -3) and then aligned against the FASTA file containing TE sequences obtained in the previous step using bowtie2 v.2.3.5.1 (ref. ^114^) (–very-sensitive –dovetail). The raw read count for each superfamily was calculated. The raw counts were normalized to the total number of sequences (reads per million, the number of aligned reads for each superfamily × 1000000/total number of reads), and then the proportion of superfamilies in the transcriptome was calculated (reads per million × 100/total number of aligned reads). To compare the RNA-seq data with the genome proportion of the respective TE superfamily, the proportion of TEs in the genome was calculated (the number of nucleotides occupied by superfamily in the genome × 100/total nucleotides occupied by TEs in the genome). The results were transformed to the log_10_ values and visualized with ggplot2^115^ and MATLAB^116^.

### Genome annotation

Genome annotation was done by an in-house pipeline (Supplementary Fig. 25) improved from a previous version^24^. First, the pipeline assessed the assembly quality using BUSCO on the basis of the Actinopterygii odb9 database^117^. The parameter -long was used for the first training of AUGUSTUS v.3.2.3 (ref. ^118^). The pipeline then identified and masked repeat elements from the assembly. Repeat elements were identified using blastx v.2.2.28+ with the protein repeat database RepeatPeps (http://www.repeatmasker.org/), and using RepeatMasker with two nucleotide repeat databases, one produced by RepeatModel (http://www.repeatmasker.org/), and the other an in-house fish repeat database combining our annotation and the one from Shao et al.^108^. Simple and low-complexity repeats were then softmasked, while those with known family were hardmasked. After repeat masking, the pipeline collected gene evidence from homology annotation, de novo annotation and RNA-seq annotation. For homology annotation we first pooled protein sequences from SWISS-PROT (www.uniprot.org) and 13 Ensembl genomes (v.95, http://www.ensembl.org): human (*Homo sapiens*), mouse (*Mus musculus*), coelacanth (*Latimeria chalumnae*), spotted gar (*Lepisosteus oculatus*), zebrafish (*Danio rerio*), cod (*Gadus morhua*), tilapia (*Oreochromis niloticus*), medaka (*Oryzias latipes*), platyfish (*Xiphophorus maculatus*), fugu (*Takifugu rubripes*), tetraodon (*Tetraodon nigroviridis*), stickleback (*Gasterosteus aculeatus*) and sea lamprey (*Petromyzon marinus*), and reduced the redundancy using CD-HIT (http://www.bioinformatics.org/cd-hit/), which resulted in 544,476 proteins. These were mapped to the assembly using exonerate v.2.2.0^119^ and Genewise2-2-0 (ref. ^120^) respectively. Before Genewise was implemented, genBlastA1.0.1 (ref. ^121^) was used to roughly locate each protein on the assembly. For de novo annotation, SNAP v.2006-07-28 (http://korflab.ucdavis.edu) and GeneMark-ES^122^ were independently used. For RNA-seq annotation, RNA-seq reads from juvenile male mixed organs, adult male muscle, spleen, skin, testis, female brain, liver and ovary were mapped and assembled using Tophat and cufflinks v.2.1.1 (ref. ^123^). In parallel, HISAT2 v.2.1.0, Trinity v.2.4.0 and PASA v.2.2.0 (refs. ^124,125^) were also used for RNA-seq read mapping and assembly. In total, 89.5% of all transcriptome reads mapped to the genome.

All gene evidence obtained from the three kinds of annotation was collected and transferred to EVidenceModeler v.1.1.1 (ref. ^126^), where gene models confirmed by all lines of evidence were extracted as high-quality gene models. They were used for the second training of AUGUSTUS. Finally, the AUGUSTUS specially trained for sterlet took all the hints from BUSCO, repeat masking and all three annotations to predict the final set of gene models for sterlet. Some broken or artificial chimaeric gene models were found and replaced by comparing the AUGUSTUS prediction with the homology gene evidence. Low-quality gene models were removed afterwards. To assign gene symbols, their protein sequences were blasted to the SWISS-PROT database (www.uniprot.org/e) (blastp v.2.2.28+ (ref. ^127^); percentage of identical matches, >20%; e-value, <1 × 10^−5^), and the symbol of the best hit was taken (https://biodbnet-abcc.ncifcrf.gov/)^128^. DeepGO was used to annotate gene ontology terms for each gene^129^.

To annotate non-coding RNAs (ncRNAs), we adapted the method from Ensembl (http://ensemblgenomes.org/info/data/ncrna). tRNAs were screened using tRNAscan-SE v.2.0.3 (ref. ^130^), and ribosomal RNAs were identified using RNAmmer^131^. The rest of the ncRNAs were then predicted using Infernal with Rfam v.14.1 (ref. ^132,133^).

### Orthology assignment

To infer gene homology among sterlet, *P. marinus* (sea lamprey), *C. milii* (elephant shark), *L. chalumnae* (coelacanth), *L. oculatus* (spotted gar), *A. gigas* (Arapaima), *S. formosus* (arowana), *O. mykiss* (rainbow trout), *S. salar* (Atlantic salmon), *T. rubripes* (Japanese fugu), *X. maculatus* (platyfish), *O. latipes* (Japanese medaka), *C. auratus* (goldfish), *C. idellus* (grass carp) and *D. rerio* (zebrafish) (see Supplementary Table 20), we used a method that reconciles species trees for the inference of orthologues. We kept the longest protein sequence for each gene and performed an all-against-all blast using blastp v.2.2.28+ with an e-value cut-off at 1 × 10^−5^ (ref. ^127^). Between each two protein sequences, the similarity distance was measured using H-score^134^, on the basis of which all protein sequences were clustered into groups (gene families) using Hcluster_sg^135^ with sea lamprey set as the outgroup. For each group, a gene tree was constructed using TreeBeST v.0.5 (ref. ^136^) with the species tree guiding. Then, on the basis of the gene tree, orthology relationships among genes were determined as *n* to *m* (*n* and *m* are positive integers; there are cases where *n* = *m*) using an in-house Perl (https://www.perl.org) script.

### Phylogenetic analysis and divergence time estimation

We reconstructed the phylogenomic tree for sterlet on the basis of one-to-one orthologues across 15 species. These protein sequences were first aligned using MUSCLE v.3.8.31 (ref. ^137^); regions with bad quality were then trimmed using trimAl^138^ with the following parameters: -gt, 0.8; –st, 0.001; –cons, 60. The resulting alignments were concatenated and transferred to RAxML v.8.2.9 (ref. ^139^) for phylogenetic tree reconstruction. The parameter PROTGAMMAAUTO was used to select the optimal amino acid substitution model. Sea lamprey was set as the outgroup, and 100 bootstraps were performed to test for robustness.

For an additional confirmation of the phylogenomic tree, we also used MrBayes v.3.2.6 (ref. ^140^). The Markov chain Monte Carlo algorithm was implemented in 3 runs with a total of 6 chains for 500,000 generations. Trees were sampled every 1,000 generations, and in the end the first 25% of the sampling were discarded as burn-in. After the burn-in threshold, the average standard deviation of split frequencies remained ≤0.01.

To infer divergence time, we used MCMCTree^141^ under a relaxed-clock model (correlated molecular clock) with approximate likelihood calculation and maximum likelihood estimation of branch lengths performed^142^. First, the phylogenetic tree and the coding sequences alignment were imported into baseml^141^ to roughly estimate the substitution rate. The substitution model was determined using modelgenerator.jar^143^. Then mcmctree was run for the first time to estimate the gradient and Hessian. The resulting file, out.BV, was then used for the final run of MCMCTree to perform approximate likelihood calculations. The final Markov chain Monte Carlo process was run for 2,005,000 steps. The first 5,000 steps were discarded as burn-in; then 20,000 samples were collected with sampling every 100 steps. We set four fossil calibrations: *O. latipes*–*T. nigroviridis* (~96.9–150.9 Ma), *D. rerio*–*G. aculeatus* (~149.85–165.2 Ma)^144^, *A. gigas*–*S. formosus* (~110–156 Ma)^145,146^ and a time for the root (<700 Ma).

### Positive selection analysis

Protein and complementary DNA fasta files from all fish (Supplementary Table 14) were downloaded. To identify orthologous proteins, all protein sequences were compared with sterlet using inparanoid^147^ with default settings. To match proteins and cDNA, sequences were blasted by tblastn, and only 100% hits were kept. Codon alignments for the protein–cDNA sequence pairs were constructed using pal2nal v.14 (ref. ^148^). The resulting sequences were aligned by MUSCLE^137^ (option: -fastaout), and poorly aligned positions and divergent regions of cDNA were eliminated by Gblocks v.0.91b (ref. ^149^) (options: -b4, 10; -b5, n; –b3, 5; –t = c). An in-house script was used to convert the Gblocks output to paml format.

For the generation of a phylogenetic tree as input for the detection of positive selection, sequences from all homologous genes, detected by inparanoid, were concatenated after the selection of conserved blocks by Gblocks and aligned using MUSCLE. The tree was generated using Phylip v.3.696^150^ with *Callorhinchus milii* (comparison1–3) or *L. chalumnae* (comparison4) as the outgroup (Supplementary Table 14). For the phylogenetic analysis by maximum likelihood, we used the Environment for Tree Exploration toolkit^151^, which automates CodeML and Slr analyses by using preconfigured evolutionary models. For the detection of genes under positive selection in sterlet, we compared the branch-specific model bsA1 (neutral) with the model bsA (positive selection) using a likelihood ratio test (FDR ≤ 0.05). To detect sites under positive selection, naive empirical Bayes probabilities for all four classes were calculated for each site. Sites with a probability >0.95 for either site class 2a (positive selection in the marked branch and conserved in the rest) were considered. The common species tree was drawn by the interactive Tree of Life tool (iTOL, https://itol.embl.de/) with default settings.

### Transcriptome analysis

Total RNA was isolated using TRIzol Reagent (Thermo Fisher Scientific) according to the supplier’s recommendation, in combination with the RNeasy Mini Kit (Qiagen). To support genome annotation, the same adult female and male sterlets (from the broodstock of the Leibniz-Institute of Freshwater Ecology and Inland Fisheries) as used for the whole-genome sequencing were sampled. RNAs were obtained from six adult male (brain, testes, muscle, spleen, liver and skin) and three adult female (ovary, liver and brain) tissues. In addition, mixed RNAs (brain, heart, eyes and spleen) of one juvenile male (20 cm) were sequenced. RNA-Seq reads were used as transcriptomic evidence for genome annotation and sex-biased expression analysis. Custom sequencing (BGI) of TruSeq libraries generated 25–30 million 100-bp paired-end reads for each sample on the Illumina Hiseq4000 platform.

For differential gene expression analysis, reads were aligned to the sterlet genome using STAR (–quantMode GeneCounts)^152^.

Owing to the sequence similarity between ohnologues, the mapping results were further filtered for uniquely mapped reads and reads with no mismatches to be able to obtain a reliable read assignment. To compare expression between different genes from an ohnologue pair, we used transcripts per million (TPM) values. For further analyses, genes not expressed (TPM < 5) in both ohnologues and in all included organs or ohnologue pairs without sufficient discriminating single nucleotide polymorphisms were excluded. Ohnologues were considered to be expressed at different levels if the absolute value (ohnologue1(log_2_TPM + 1) − ohnologue2(log_2_TPM + 1)) was greater than one (representing a twofold difference) in at least two different sterlet organs and developmental stages. For functional clustering, the web tool DAVID (https://david.ncifcrf.gov/) was used, on the basis of human orthologues and all ohnologues as background.

### RAD-tag sequencing and analysis of sex-specific tags

The genomic DNA of 31 females and 30 males was extracted from 90% ethanol-preserved fin clips using a classical phenol/chloroform protocol. The sterlet RAD-tag library was built according to standard protocols^153^, using Sbf1 as a single restriction enzyme, and sequenced on a single lane of Hiseq 2500, using the v4 SR100nt mode. The resulting read file was then demultiplexed using the process-radtags.pl script of STACKS software v.1.44 (ref. ^154^) with default settings.

Demultiplexed reads were analysed with RADSex v.0.2.0^155^. RADSex sorts reads from the demultiplexed dataset into groups sharing the exact same sequence, and reads that would belong to the same polymorphic locus using standard analysis software are simply split into multiple markers. As a result, RADSex markers are non-polymorphic, thus allowing straightforward presence–absence comparison between individuals.

First, a table of depth for each RADSex marker in each individual from the dataset was generated using radsex *process* with default settings. The distribution of markers in males and females was then computed with radsex *distrib*, using a minimum depth of 10 (--min-cov 10) to consider a marker present in an individual, and a tile plot was generated from this distribution using the plot_sex_distribution() function from RADSex-vis (https://github.com/RomainFeron/RADSex-vis). The same analysis was performed with minimum depths of 1, 2 and 5, but the results were not qualitatively affected. A total of 176,735 markers were obtained that were present in at least one individual with a minimum depth of 10.

### Reporting Summary

Further information on research design is available in the Nature Research Reporting Summary linked to this article.

## Supplementary information

Supplementary Information Supplementary Figs. 1–28 and Notes 1–13.

Reporting Summary

Supplementary Tables Supplementary Tables 1–22.

## Data Availability

The *Acipenser ruthenus* Whole Genome Shotgun project has been deposited at DDBJ/ENA/GenBank under the accession number VTUV00000000. The version described in this paper is version VTUV01000000. Genomic and transcriptomic reads are deposited in the Sequence Read Archive under accession numbers SRR10188515-10188518 and SRR11013451-11013458.

## Code availability

The in-house scripts have been deposited in Github (https://github.com/dukecomeback/sterlet_Msch).
