# Supplementary material for: The sterlet sturgeon genome sequence and the mechanisms of segmental rediploidization
Source: Nat Ecol Evol. 2020 Mar 30;4(6):841–52. doi: 10.1038/s41559-020-1166-x (PMC7269910; doi:10.1038/s41559-020-1166-x)
Supplement: Supplementary file 1 — Supplementary Figs. 1–28 and Notes 1–13. [file 41559_2020_1166_MOESM1_ESM.pdf]

nature ecology & evolution

SUPPLEMENTARY INFORMATION
https://doi.org/10.1038/s41559-020-1166-x

In the format provided by the authors and unedited.

The sterlet sturgeon genome sequence and the mechanisms of segmental rediploidization

Kang Du[1,2], Matthias Stöck[3] ✉, Susanne Kneitz[1], Christophe Klopp[4,5], Joost M. Woltering[6], Mateus Contar Adolfi[1], Romain Feron[7], Dmitry Prokopov[8], Alexey Makunin[8], Ilya Kichigin[8], Cornelia Schmidt[1], Petra Fischer[1], Heiner Kuhl[3], Sven Wuertz[3], Jörn Gessner[3], Werner Kloas[3], Cédric Cabau[4,5], Carole Iampietro[9], Hugues Parrinello[10], Chad Tomlinson[11], Laurent Journot[10], John H. Postlethwait[12], Ingo Braasch[13], Vladimir Trifonov[8], Wesley C. Warren[14], Axel Meyer[6], Yann Guiguen[15] and Manfred Schartl[2,16,17] ✉

[1]Physiological Chemistry, Biocenter, University of Wuerzburg, Wuerzburg, Germany. [2]Developmental Biochemistry, Biocenter, University of Wuerzburg, Wuerzburg, Germany. [3]Leibniz-Institute of Freshwater Ecology and Inland Fisheries, IGB, Berlin, Germany. [4]Plate-forme Bio-informatique Genotoul, Mathématiques et Informatique Appliquées de Toulouse, INRA, Castanet-Tolosan, France. [5]SIGENAE, GenPhySE, Université de Toulouse, INRA, ENVT, Castanet-Tolosan, France. [6]Lehrstuhl für Zoologie und Evolutionsbiologie, Department of Biology, University of Konstanz, Konstanz, Germany. [7]Department of Ecology and Evolution, University of Lausanne, and Swiss Institute of Bioinformatics, Lausanne, Switzerland. [8]Institute of Molecular and Cellular Biology, Siberian Branch of the Russian Academy of Sciences, Novosibirsk State University, Novosibirsk, Russia. [9]INRAE, US 1426, GeT-PlaGe, Genotoul, Castanet-Tolosan, France. [10]Montpellier GenomiX (MGX), c/o Institut de Génomique Fonctionnelle, Montpellier, France. [11]McDonnell Genome Institute, Washington University School of Medicine, St. Louis, MO, USA. [12]Institute of Neuroscience, University of Oregon, Eugene, OR, USA. [13]Department of Integrative Biology, Michigan State University, East Lansing, MI, USA. [14]Bond Life Sciences Center, University of Missouri, Columbia, MO, USA. [15]INRA, UR1037 LPGP, Fish Physiology and Genomics, Rennes, France. [16]The Xiphophorus Genetic Stock Center, Department of Chemistry and Biochemistry, Texas State University, San Marcos, TX, USA. [17]Hagler Institute for Advanced Study and Department of Biology, Texas A&M University, College Station, TX, USA. ✉ e-mail: matthias.stoeck@igb-berlin.de; phch1@biozentrum.uni-wuerzburg.de

nature ecology & evolution

SUPPLEMENTARY INFORMATION
https://doi.org/10.1038/s41559-020-1166-x

In the format provided by the authors and unedited.

The sterlet sturgeon genome sequence and the mechanisms of segmental rediploidization

Kang Du[1,2], Matthias Stöck[3] ✉, Susanne Kneitz[1], Christophe Klopp[4,5], Joost M. Woltering[6], Mateus Contar Adolfi[1], Romain Feron[7], Dmitry Prokopov[8], Alexey Makunin[8], Ilya Kichigin[8], Cornelia Schmidt[1], Petra Fischer[1], Heiner Kuhl[3], Sven Wuertz[3], Jörn Gessner[3], Werner Kloas[3], Cédric Cabau[4,5], Carole Iampietro[9], Hugues Parrinello[10], Chad Tomlinson[11], Laurent Journot[10], John H. Postlethwait[12], Ingo Braasch[13], Vladimir Trifonov[8], Wesley C. Warren[11], Axel Meyer[6], Yann Guiguen[14] and Manfred Schartl[2,15,16] ✉

[1]Physiological Chemistry, Biocenter, University of Wuerzburg, Wuerzburg, Germany. [2]Developmental Biochemistry, Biocenter, University of Wuerzburg, Wuerzburg, Germany. [3]Leibniz-Institute of Freshwater Ecology and Inland Fisheries, IGB, Berlin, Germany. [4]Plate-forme Bio-informatique Genotoul, Mathématiques et Informatique Appliquées de Toulouse, INRA, Castanet-Tolosan, France. [5]SIGENAE, GenPhySE, Université de Toulouse, INRA, ENVT, Castanet-Tolosan, France. [6]Lehrstuhl für Zoologie und Evolutionsbiologie, Department of Biology, University of Konstanz, Konstanz, Germany. [7]Department of Ecology and Evolution, University of Lausanne, and Swiss Institute of Bioinformatics, Lausanne, Switzerland. [8]Institute of Molecular and Cellular Biology, Siberian Branch of the Russian Academy of Sciences, Novosibirsk State University, Novosibirsk, Russia. [9]INRAE, US 1426, GeT-PlaGe, Genotoul, Castanet-Tolosan, France. [10]Montpellier GenomiX (MGX), c/o Institut de Génomique Fonctionnelle, Montpellier, France. [11]Bond Life Sciences Center, University of Missouri, Columbia, MO, USA. [12]Institute of Neuroscience, University of Oregon, Eugene, OR, USA. [13]Department of Integrative Biology, Michigan State University, East Lansing, MI, USA. [14]INRA, UR1037 LPGP, Fish Physiology and Genomics, Rennes, France. [15]The Xiphophorus Genetic Stock Center, Department of Chemistry and Biochemistry, Texas State University, San Marcos, TX, USA. [16]Hagler Institute for Advanced Study and Department of Biology, Texas A&M University, College Station, TX, USA. ✉e-mail: matthias.stoeck@igb-berlin.de; phch1@biozentrum.uni-wuerzburg.de

Supplementary Note

The sterlet sturgeon genome sequence and the mechanisms of segmental rediploidization

Du Kang[1], Matthias Stöck[2,*], Susanne Kneitz[1], Christophe Klopp[3], Joost Woltering[4], Mateus Adolfi[1], Romain Feron[5], Dmitry Prokopov[6], Alexey Makunin[6], Ilya Kichigin[6], Cornelia Schmidt[1], Petra Fischer[1], Heiner Kuhl[2], Sven Wuertz[2], Jörn Gessner[2], Werner Kloas[2], Cedric Cabau[3], Carole Iampietro[7], Hugues Parrinello[8], Chad Tomlinson[9], Laurent Journot[8], John H. Postlethwait[10], Ingo Braasch[11], Vladimir Trifonov[6], Wesley C. Warren[9,12], Axel Meyer[4], Yann Guiguen[13], Manfred Schartl[14,15,16*]

[1] Physiological Chemistry, Biocenter, University of Wuerzburg, 97074 Wuerzburg, Germany

[2] Leibniz-Institute of Freshwater Ecology and Inland Fisheries, IGB, Müggelseedamm 301, D-12587 Berlin, Germany

[3] Plate-forme Bio-informatique Genotoul, Mathématiques et Informatique Appliquées de Toulouse, INRA, Castanet Tolosan, France and SIGENAE, GenPhySE, Université de Toulouse, INRA, ENVT, Castanet Tolosan, France

[4] Lehrstuhl für Zoologie und Evolutionsbiologie, Department of Biology, University of Konstanz, Universitätsstraße 10, 78457 Konstanz, Germany

[5] Department of Ecology and Evolution, University of Lausanne, and Swiss Institute of Bioinformatics, 1015 Lausanne, Switzerland.

[6] Institute of Molecular and Cellular Biology, Siberian Branch of the Russian Academy of Sciences, Novosibirsk State University, 630090 Novosibirsk, Russia.

[7] INRA, US 1426, GeT-PlaGe, Genotoul, Castanet-Tolosan, France

[8] Montpellier GenomiX (MGX), c/o Institut de Génomique Fonctionnelle, 141 rue de la cardonille, 34094 Montpellier Cedex 05, France

[9] Bond Life Sciences Center, University of Missouri, Columbia, MO USA

[10] Institute of Neuroscience, University of Oregon, Eugene, Oregon, OR 97401, USA

[11] Department of Integrative Biology, Michigan State University, MI 48824, USA
[12] Bond Life Sciences Center, University of Missouri, Columbia, MO, USA
[13] INRA, UR1037 LPGP, Fish Physiology and Genomics, F-35042 Rennes, France
[14] The Xiphophorus Genetic Stock Center, Department of Chemistry and Biochemistry, Texas State University, San Marcos, Texas, USA
[15] Hagler Institute for Advanced Study and Department of Biology, Texas A&M University, College Station, Texas 77843, USA
[16] Developmental Biochemistry, Biocenter, University of Wuerzburg, 97074 Wuerzburg, Germany

[*] Corresponding authors

Supplementary Note 1. Genome assembly

The first smartdenovo assembly of the PacBio reads had a length of 1.56 Gb (Supplementary Table 1) which is 16.1% lower than the expected 1.86-1.87 (http://www.genomesize.com) and showed multiple contigs having twice the expected raw read coverage. 1193 contigs, corresponding to 280 Mb had a twice larger depth as expected and were processed with freebayes, HAPCUT2, fgbio and vcf-consensus to generate haplotyped contigs. Only 83 contigs did not end in a single haplotyped segment and had to be split in different sub-contigs. 1,110 contigs were haplotyped as a single segment. The single and multiple segment haplotype contigs corresponded to 472 Mb and 88 Mb, respectively. The re-duplication led to an assembly size of 1.84 Gb and the assembly did not present the double coverage pattern (Supplementary Table 1). In the following step the Hi-C data were used for scaffolding and manual inspection, which decreased assembly size to 1.8Gb because all re-duplicated contigs showing no link to other contigs were removed.

Supplementary Note 2. B chromosome

B chromosomes (Bs) are enigmatic accessory elements to the regular chromosome set (A). They are found in some but not all individuals within a population and are considered either non-functional, beneficial or harmful[1]. B chromosome refers to those chromosomes that are essential for life and may be lacking in some individuals[1]. For scaffold 60 we noticed a high content of repeat elements (89.7%) and only three low quality genes annotated (two failed to be supported by transcript evidence, another showing protein similarity to XP_028669235 but is only a fragment of a full length orthologue) (Supplementary Table 2). Additionally, the gene evidence from homology collected for scaffold 60 revealed that all protein alignments either contained frameshift/premature stop or a fragmented alignment (< 30% alignment). When assembled transcripts from the RNA-seq data were used, all mapped transcripts had no blast hit to the NR database. Moreover, no ncRNA was found on this scaffold. Taking together, scaffold 60 most probably represents a fully assembled B-chromosome.

Supplementary Note 3. Relative rate of gene evolution

To compare the molecular evolutionary rate between the sterlet lineage and the other fish, we first collected 275 one-to-one orthologs among sterlet, medaka, platyfish, fugu,

zebrafish, arapaima, arowana, spotted gar, coelacanth, elephant shark and sea lamprey. Protein sequences for each ortholog were aligned using MUSCLE and trimmed using trimAl. The 275 alignments were then concatenated into a super-alignment. From this we reconstructed phylogenomic trees using RAxML and Mrbayes respectively. In particular, from the super alignment we retrieved the Fourfold Degenerate Synonymous Site (4DTV) and used it to optimize the branch length of RAxML tree. Hence in total we obtained three phylogenomic trees to compare molecular evolutionary rate between different lineages.

Lineage pairwise distance was calculated using cophenetic.phylo[2] for all three trees (Supplementary Table 4). With sea lamprey as outgroup we found that sterlet evolved almost as slow as coelacanth or elephant shark, and clearly slower than teleosts.

Surprisingly, suggested by Tajima's relative rate test and two-cluster test implemented by MEGA7 (https://www.megasoftware.net/) and LINTRE (http://www.kms.ac.jp/~genomelb/takezaki/lintre/index.html) respectively (Supplementary Table 5, 6), sterlet is the slowest evolving in comparison with elephant shark, coelacanth and gar.

Supplementary Note 4. Time inference for the sterlet whole genome duplication

The age of WGDs were normally deduced linearly based on the pairwise dS (synonymous substitutions) values of ohnolog pairs and the amount of MY (million year) a dS unit represents[3 4]. However, synonymous substitutions in different lineage are accumulated in different speed, hence it is important to make calibration from an event happened in the lineage or a close lineage. In a previous study, the age of Ss4R (salmonid-specific 4th WGD) was deduced to ~100 mya based the divergence time of Atlantic salmon and rainbow trout. Our recalculation revealed the same results, however, when used as the calibration the divergence time of rainbow trout and spotted gar, Ss4R was wrongly estimated to 33 mya (Supplementary Table 21).

Given the ancient origin and slow molecular evolutionary rate of sterlet, the divergence of sterlet and spotted gar is hardly to be an appropriate calibration. Instead we made use of the available transcriptomes of five sturgeon species (*Acipenser baerii*, *Acipenser*

oxyrinchus, *Acipenser schrencki*, *Acipenser sinensis* and *Acipenser transmontanus*; http://publicsturgeon.sigenae.org/home.html). Among the five sturgeons, spotted gar, arapaima, Asian arowana, medaka, fugu, zebrafish and spotted gar; 387 one-to-one orthologs were identified to reconstruct the phylogeny tree using RAxML 8.2.9 and MrBayes 3.2.6[5 6]; and to infer the divergence time using MCMCTree[7] (Supplementary Fig. 6a). The result is mainly in agreement with a previous study[8].

We then calculated the pairwise dS for 9914 sterlet ohnolog pairs, 9009 one-to-one orthologous pairs between sterlet and *A. baerii*, 7893 between sterlet and *A. sinensis*, 8540 between sterlet and *A. schrencki*, 7939 between sterlet and *A. transmontanus*, and 6289 between sterlet and *A. oxyrinchus* using codeML under "-runmodel=-2" (Supplementary Fig. 6b; Supplementary Table 10). Results with S*dS <=1 were discarded. The alignment between two sequences was first constructed using MAFFT[9] in amino acid sequence then translated to coding sequence using pal2nal[10].

According to the results, the pairwise dS between sterlet ohnolog pairs (median value 0.068) is even larger than that of one-to-one orthologous pairs between sterlet and *A. oxyrinchus* (median value 0.059), indicating the WGD happened before the divergence of sterlet and *A. oxyrinchus*. To verify that we collected 8159 pairs of sterlet ohnolog with their orthologs in the five other sturgeon species and constructed gene tree for each group using TreeBeST 0.5.1[11]. Topology of gene trees confirmed that the WGD happened before the divergence of sterlet and *A. oxyrinchus* (for examples see Supplementary Fig. 26).

Given that the dS value between sterlet and *A. oxyrinchus* and that among sterlet ohnolog pairs is closest, we use their divergence as the time calibration, and deduced that the WGD happened at around 170 (121~237) mya.

Supplementary Note 5. DNA sequence alignment revealing ohnology and arm exchange between chromosomes in sterlet

According to the chord diagram of sterlet (Fig. 2b), chromosome 1 and 2, 3 and 4, 5 and 6, 8 and 9 show homoeology and common ancestry over their whole lengths, while chromosome 7, 10-31 and 36 reveal more complex structural relations. The remaining small chromosomes (32-35 and 37-55) have lost their homeologous counterpart completely.

To verify this pattern, we aligned the DNA sequences of those homeologous chromosome pairs using LAST (http://last.cbrc.jp/) under instruction of example “2017 human-ape alignments” (https://github.com/mcfrith/last-genome-alignments). Alignments with error probability > 1e-8 were discarded.

The results confirmed the homeology relationships as revealed in the chord diagram (Supplementary Fig. 7, 8, 13), and deciphered a history of chromosomal translocations and inversions. Intriguingly, the break of homology frequently is located in the centre of the metacentric chromosomes. A peak of repeat element content in the same region can be taken as evidence that these are the centromeres (Supplementary Fig. 8, 11), and that entire chromosome arms were reciprocally exchanged.

Supplementary Note 6. Sequencing of single sterlet chromosomes validates genome wide assembly and ohnology relationships

We studied several pairs of sterlet paralogous chromosomes with different morphology: the paralogous pairs of large chromosomes ARU1/ARU2, ARU3/ARU4, ARU5/ARU6, ARU8/9 and two paralogous regions on chromosome ARU7.

We previously generated chromosome-specific sequence libraries from microdissected *A. ruthenus* metaphase chromosomes[12,13] (Supplementary Fig. 9). Following amplification and Illumina sequencing, the datasets representing sterlet chromosomes ARU1, ARU2, ARU3, ARU4, ARU5, ARU6, ARU7, ARU8, ARU9, ARU13 and ARU14 were obtained. We applied DOPseq to analyze each dataset[14] (https://github.com/lca-imcb/dopseq): we aligned the reads from chromosome specific library onto sterlet scaffolds. We only analyzed regions with p-value <0.01.

Most reads from sequenced chromosomes (ARU1 - ARU9) densely marked corresponding scaffolds (from HiC_scaffold_1 to HiC_scaffold_9). Besides, reads from each chromosome revealed additional signals on paralogous scaffolds (or scaffold parts) (Supplementary Fig. 10; Supplementary Data 1).

This confirmed previously obtained physical mapping data, when single chromosome microdissection derived libraries painted in whole mount in-situ hybridizations two paralogous regions in sterlet genome[12].

Thus, using sequences from microdissected sterlet chromosomes we could unambiguously assign scaffolds to physical chromosomal regions and determined paralogous regions.

Supplementary Note 7. Double conserved synteny, identification of ohnolog/singleton, and WGD retention rate

A WGD in the sterlet genome is suggested by a dS plot of sterlet paranome[15] (Supplementary Fig. 5).

To confirm and reveal the WGD pattern of sterlet, we mapped 18341 gar genes (http://www.ensembl.org/Lepisosteus_oculatus/Info/Annotation) to the sterlet genome. Based on sequence similarity and conserved microsynteny (at least four genes arranged in a row with a gap of less than 15 genes), 12216 gar genes were confirmed as single-copy orthologs to 22211 sterlet genes (Supplementary Table 9). 8764 gar genes mapped onto two different sterlet chromosomes, while 3452 genes interspersed between ohnologs mapped only to one sterlet chromosome, resulting in a WGD retention rate of 71.7%.

Considering a single species as outgroup (here: species that did not undergo the WGD) may cause reduced identification of orthologs and thus ohnologs or singletons. Hence, we added coelacanth and elephant shark as outgroup to identify ohnologs and singletons in sterlet. We first included 11765 pairs of paralogs in sterlet that have only a single-copy ortholog in gar, coelacanth or elephant shark, then confirmed 9914 of them to show paralogous synteny (at least 5 genes ranked in a row with a gap of less than 15 genes). These genes are considered to be high fidelity ohnologs. For detailed information about location and corresponding single-copy genes in outgroup species see Table sterlet_ohno for DCS checking (Supplementary Table 8).

With this conservative criterion, we also identified 10050 ohnolog pairs in Atlantic salmon and 10210in rainbow trout, as results from the Ss4R; 8383 in goldfish, resulting from the carp WGD (Cs4R). To depict ohnology relationship between chromosomes, we investigated on which chromosome each ohnolog is located, and generated the chord diagram for sterlet (Fig. 2b), goldfish, rainbow trout (Supplementary Fig. 24) and Atlantic salmon (Supplementary Fig. 27) using circos[16] or package “circlize” in R[17].

Singletons were defined as those genes with "one to one" orthology in other species which did not experience the WGD. We identified 4175 singletons in sterlet, 8832 in Atlantic salmon, 8998 in rainbow trout and 6754 in goldfish, as results from the rediploidization of the corresponding special WGD. The presence of 9914 ohnolog pairs and 4175 singletons in sterlet results in a duplicate retention rate of 70%, confirming to the estimation from the DCS analysis above.

The remaining genes ("undefined genes") were neither categorized as ohnolog or singleton from the latest WGD, either because their single-copy orthology relationships were lost in the species that did not experience this WGD or because they resulted from an older WGD, or because they had relationships other than 1:1 or 2:1 (which means the gene is a local duplication in either one or both species).

To reveal the pattern of deduplication all ohnologs, singletons, undefined genes and their location information on chromosomes, were depicted for sterlet, goldfish, Atlantic salmon and rainbow trout on loci-plots (Supplementary Fig. 12).

Supplementary Note 8. Gene fate after Ars3R

Deduplication, subfunctionalization and neofunctionalization are suggested to be the three possible fates of gene pairs after gene duplication[18]. The dN/dS value and expression patterns can give clues for investigating the fate of paralogous genes.

In sterlet, we found 4175 singletons and 9914 pairs of ohnolog, indicating a 70-% deduplication rate as a result from Ars3R, higher to the rates in goldfish (43.7%), Atlantic salmon (46.7%) and rainbow trout （46.9%, despite the different time when each WGD had happened (sterlet ~180mya, goldfish ~14mya and salmonids ~95mya) (Supplementary Table 11). dN/dS value was calculated to evaluate the selection pressure. For each singleton or ohnolog pair, we collected their one-copy orthologs in other species. Protein sequences were aligned using MAFFT[19] and transformed to CDS using pal2nal[10]. Gaps were trimmed using Gblocks[20]. Then for each alignment, an unrooted gene tree was reconstructed using QuickTree 2.5 guided by the species phylogeny[21]. We calculated the dN/dS value using codeML under branch-free model. No GO terms are significantly enriched for the common singletons (using fdr-p value).

When the dN/dS values were compared between sterlet singletons and ohnologs, we found that ohnolog present a higher percentage with high dN/dS values than singleton (Supplementary Fig. 20), indicating less stringent purifying selection on ohnologs.

To test for positive selection of each ohnolog pair, we implemented an LRT (likelihood-ratio test) between two pairwise models using PAML[7]. In the null model we set the omega to 0.5, while in the alternative model, the omega was freely estimated. Only those ohnolog pairs with LRT p-value smaller than 0.05, alternative lnL (log-Likelihood) larger than null lnL and the estimated omega higher than 0.5 were scored as being under positive selection. Out of 9914 ohnolog pairs of sterlet 207 such pairs were found.

To investigate the expression status of ohnologs we extracted the TPM for each paralog from the RNA-seq data of 23 different sterlet organs and developmental stages. To be able to assign reads with high confidence to one of the ohnologs we filtered the alignment file for uniquely aligned reads with no mismatches. Genes with either no discriminating SNPs or unexpressed in any of the samples (TPM<5) were excluded from further analyses (n=671). The remaining 4369 pairs were categorized either as either showing similar expression from both ohnologs in all samples (n=1139) or showing different expression patterns in at least two samples (n= 3230) (Supplementary Fig. 16, 28). Within the last group we found 38 pairs with only one gene expressed in all samples, the other being unexpressed in all samples (Supplementary Fig. 28). For 341 ohnolog pairs duplicates expression was partitioned between different organs or developmental stages, indicating subfuntionalization.

Supplementary Note 9. Comparison of the conservation of synteny from Ars3R with other WGDs

To compare the Ars3R with the teleost WGDs the gar genome was used as reference. 16243 spotted gar genes and their "1 to X" (X>=1) orthologs in Atlantic salmon, rainbow trout, goldfish, zebrafish, medaka, arapaima and sterlet were investigated. To identify duplicated genes, which are the result of a WGD rather than local gene duplications, we only included those rows with pairwise synteny confirmed, meaning at least 4 genes to be ranked in a row with gap size of less than 15 genes.

In the end, 15216 gar genes were kept for the analysis. By checking their orthologs' location on chromosomes, we found 27 genes with their orthologs located on at least two different chromosomes in sterlet, arapaima, zebrafish, medaka, and on at least four chromosomes in Atlantic salmon, rainbow trout and goldfish (Supplementary Table 12), indicating they were always retained after WGDs. In addition, 191 genes have their orthologs always located on one chromosome in each species, indicating they were always deduplicated (Supplementary Table 13).

To investigate if these genes were retained or deduplicated by chance, we ran 10,000 time simulations under a stochastic process of keeping or losing duplication after WGDs (Supplementary Fig. 17). Results show that the observed counts are always higher than expectation distribution, indicating that number of commonly retained or duplicate lost gene is above the stochastic expectation.

Intriguingly, according to their location on gar chromosomes, we found amongst the 191 genes that always were deduplicated 102 genes neighboring each other (with in between not more than 5 genes); and 39 genes arranged in 8 synteny blocks (with at least 4 genes in a row a gap of less than 15 genes). These are significantly higher numbers than under expectation of a random process (10,000 bootstraps of 191 no-return resampling from the 15216 gar genes; Supplementary Fig. 18, 19), indicating it is not a stochastic process. In summary, this indicates that many genes evolved dependent on their physical distance, namely, that if one gene is lost this leads to the "death" of its neighbor.

Supplementary Note 10. Expansion and contraction of gene families

CAFE 4.2[22] was used to analyze the dynamic of gene family size. We imported the gene group (family) size resulting from Hcluster_sg, and a corresponding species tree adapted from TIMETREE database (http://www.timetree.org/)). Gene families were defined by clustering 445,487 genes from 15 species after an all vs. all blast. Since CAFE assumes that each family has at least one gene at the root of the tree, we only included those gene families into the analysis that occur in more than 12 branches. Also failure of CAFE could be caused by a very large change in gene family size on a single branch. 8,139 gene families are present in the most recent common ancestor (MRCA) of all taxa and have <100 gene copies, hence qualified for the analysis of gene family size dynamics. We put aside the

gene families with one or more species that have ≥ 100 gene copies, and analyzed them later with estimated parameter values.

To build model 1, we set that all the branches share a single changing rate (λ), and ran 1000 Monte Carlo random samplings with p value threshold of 0.01 to search for the λ value. Then we built model 2 by setting different λ for the branches leading to sterlet branch, representing Ars3R; to Atlantic salmon, rainbow trout, and goldfish, representing the Ss4R and Cs4R, to the rest of teleost branches, representing branches that only underwent the Ts3R; and to the rest of the tree (underwent 1R and 2R). The two models were compared by a likelihood test based on 100 simulations. The results suggested that model 2 fit better than model 1, and the branches with 4R and Ars3R (λ 0.0062 and 0.0017) have their gene family changed much faster than in branches with more ancient polyploidization (λ 0.0007 and 0.0004).

Since model 2 had a better fit it was used to parse the gene family size data. At last, a gene family was reported as significantly changed in size only when the p value was <0.01. In goldfish, 597 gene families expanded and five contracted, in Atlantic salmon ten expanded, in rainbow trout two expanded and one contracted, in sterlet 63 expanded and three contracted (Supplementary Table 22). No common gene family was detected to expand or contract in all four tetraploid lineages.

Supplementary Note 11. Ab-initio annotation of zp gene family

To identify zona pellucida genes in sterlet, arapaima, coelacanth, elephant shark, gar, goldfish, medaka, Atlantic salmon, Tanaka snailfish, rainbow trout, zebrafish (Supplementary Table 20), Antarctic blackfin icefish[23], Mariana hadal snailfish and Tanaka snailfish[24], we adapted the method used for identification of olfactory receptor genes[25]. First we collected 117 zona pellucida proteins from the previous study[26], and used them as query to blast to the assemblies using blastp[27]. Results with alignment less than 40 aa were discarded. Then to determine the gene structure, each query protein was aligned to its hit region using GeneWise[28]. This method identified 130 zona pellucida genes in Antarctic blackfin icefish, similar to[23] and 116 in sterlet.

Supplementary Note 12. Evolution of sterlet Hox clusters after genome tetraploidization and inference of the ancestral vertebrate Hox complement

Hox genes are highly conserved developmentally active transcription factors, which have been widely used to understand gene evolution after genome duplications, generally within the context of subfunctionalization, degeneration or neofunctionalization[29]. The genomic history of vertebrate *Hox* clusters was shaped by the 1R and 2R rounds of duplication leading to four original gnathostome *Hox* clusters (*Hoxa-d*) that are maintained as the minimal *Hox* complement in all vertebrates. After their 3R duplication, the rapidly evolving teleosts underwent extensive loss and remodelling of their initial eight *Hox* clusters (*Hoxaa* - *Hoxdb*)[30-34] as well as subsequent subfunctionalization of ohnologs[35,36]. Analysis of the sterlet genome finds 88 *hox* genes arranged in eight clusters (Fig. 4a). An intact *hoxd14* gene is present on chromosome 12 whereas the *hoxd14* ohnolog on chromosome 10 has been pseudogenized through several frameshift mutations in exon1 and exon2. Interestingly, selective loss of one *hoxd14* ohnolog has apparently independently occurred in Polyodon[37]. No further loss or pseudogenization of *hox* genes was detected. Therefore, the fates of *hox* genes following genome duplications in sterlet and teleosts differs strongly. LAGAN Vista comparison of the *hoxd* flanking gene desserts, which are involved in the long-range transcriptional regulation of the cluster[38-41], indicates that all ultra conserved elements shared with gar are retained in each of the sterlet's ohnologous *hoxd* synteny regions (Supplementary Fig. 21). This suggests that the low divergence of the sterlet *Hox* clusters extends to their regulatory regions and strengthens the hypothesis of a slow post-tetraploidization evolution. *Hoxa14*, *hoxd5* or *hoxb14* were not detected in the slowly evolving sterlet genome. This indicates an extreme stability of the number of hox genes present in the early branching ray finned fish, with an identical *hox* complement in gar and sterlet, that share a last common ancestor ~335MYA. This provides further evidence for a scenario whereby *hoxd5* and *hoxb14* were lost in the common ancestor of bony vertebrates (Osteichthyes) and *hoxa14* in the common ancestor of actinopterygians[41] (Figure hox/b

Supplementary Note 13. Glutamate receptor ohnolog retention following the sterlet genome duplication

We and others have previously found that following the Teleost WGD (TS3R), nervous system and neuronal genes with functions in cognition and/or behavior particularly often escaped the non-functionalization fate and were over-retained in teleosts as ohnologous pairs compared to the genome-wide background TGD ohnolog retention rate [e.g.[42-44]].

Our previous survey[42] furthermore revealed that among these nervous system genes, glutamate receptor (GRGs) genes show particularly high Ts3R ohnolog retention rates: clupeocephalan teleosts such as medaka and zebrafish have retained 74.1% and 70.4% (20/27 and 19/27; Supplementary Fig. 22; Supplementary Table 18) of GRGs as Ts3R ohnologous pairs, respectively, even after more than 200 million years since the Ts3R duplication event. This exceptionally high ohnolog retention rate is seen across teleost lineages, as e.g. the distantly related osteoglossiform teleost arowana has kept 70.4% (19/27; Supplementary Fig. 22; Supplementary Table 18) of GRG Ts3R ohnologs as well.

Here we asked whether a convergent trend is observed following the sterlet whole genome duplication (Ars3R) event. Using the gene annotation as a guide, we generated a manually curated annotation of sterlet orthologs of 27 GR genes of both the metabotropic and ionotropic type as present in the spotted gar. Spotted gar thereby serves as an "unduplicated" ray-finned outgroup to both the sterlet and the teleost genome duplications.

An overview of our GRG ohnolog survey in sterlet compared to human, gar, and the teleost representatives zebrafish, medaka, and arowana is shown (Supplementary Fig. 22); accession numbers are given (Supplementary Table 18).

Of the 27 GRGs present in gar, 26 were at least present in one copy in the sterlet genome. Ionotropic NMDA gene *grin2B* was not found in the sterlet genome at all. At this point, we cannot distinguish between a loss of this gene in the sturgeon lineage before the Ars3R event or independent losses of both Ars3R ohnologs following duplication. Hence, *grin2B* was excluded from calculating the Ars3R GRG ohnolog retention rate for sterlet.

We found that 23 of 26 GR genes have retained their Ars3R ohnolog after the sterlet-specific genome duplication, resulting in an ohnolog retention rate of 88.5%, which is

significantly higher than the genome-wide Ars3R ohnolog retention rate of 70% [8,534 Ars3R ohnolog pairs. Thus, GRG genes have been convergently over-retained following the Ars3R and Ts3R genome duplication events compared to the genome-wide average although to a lower extent in sterlet than in teleosts.

1 Valente, G. T. *et al.* B chromosomes: from cytogenetics to systems biology. *Chromosoma* **126**, 73-81 (2017).

2 Paradis, E., Claude, J. & Strimmer, K. APE: analyses of phylogenetics and evolution in R language. *Bioinformatics* **20**, 289-290 (2004).

3 Berthelot, C. *et al.* The rainbow trout genome provides novel insights into evolution after whole-genome duplication in vertebrates. *Nature communications* **5**, 3657 (2014).

4 Chen, Z. *et al.* De novo assembly of the goldfish (Carassius auratus) genome and the evolution of genes after whole-genome duplication. *Science Advances* **5**, eaav0547 (2019).

5 Ronquist, F. *et al.* MrBayes 3.2: efficient Bayesian phylogenetic inference and model choice across a large model space. *Systematic biology* **61**, 539-542 (2012).

6 Stamatakis, A. RAxML version 8: a tool for phylogenetic analysis and post-analysis of large phylogenies. *Bioinformatics* **30**, 1312-1313 (2014).

7 Yang, Z. PAML 4: phylogenetic analysis by maximum likelihood. *Molecular biology and evolution* **24**, 1586-1591 (2007).

8 Luo, D. *et al.* Highly Resolved Phylogenetic Relationships within Order Acipenseriformes According to Novel Nuclear Markers. *Genes* **10**, 38 (2019).

9 Castresana, J. Selection of conserved blocks from multiple alignments for their use in phylogenetic analysis. *Molecular biology and evolution* **17**, 540-552 (2000).

10 Suyama, M., Torrents, D. & Bork, P. PAL2NAL: robust conversion of protein sequence alignments into the corresponding codon alignments. *Nucleic acids research* **34**, W609-W612 (2006).

11 Ponting, C. (2007).

12 Romanenko, S. A. *et al.* Segmental paleotetraploidy revealed in sterlet (Acipenser ruthenus) genome by chromosome painting. *Molecular cytogenetics* **8**, 90 (2015).

13 Andreyushkova, D. *et al.* Next generation sequencing of chromosome-specific libraries sheds light on genome evolution in paleotetraploid sterlet (Acipenser ruthenus). *Genes* **8**, 318 (2017).

14 Makunin, A. I. *et al.* Contrasting origin of B chromosomes in two cervids (Siberian roe deer and grey brocket deer) unravelled by chromosome-specific DNA sequencing. *BMC genomics* **17**, 618 (2016).

15 Maere, S. *et al.* Modeling gene and genome duplications in eukaryotes. *Proceedings of the National Academy of Sciences* **102**, 5454-5459 (2005).

16 Krzywinski, M. *et al.* Circos: an information aesthetic for comparative genomics. *Genome research* **19**, 1639-1645 (2009).

17 Gu, Z., Gu, L., Eils, R., Schlesner, M. & Brors, B. circlize implements and enhances circular visualization in R. *Bioinformatics* **30**, 2811-2812 (2014).

18 Lien, S. *et al.* The Atlantic salmon genome provides insights into rediploidization. *Nature* **533**, 200 (2016).

19 Nakamura, T., Yamada, K. D., Tomii, K. & Katoh, K. Parallelization of MAFFT for large-scale multiple sequence alignments. *Bioinformatics* **34**, 2490-2492 (2018).

20 Talavera, G. & Castresana, J. Improvement of phylogenies after removing divergent and ambiguously aligned blocks from protein sequence alignments. *Systematic biology* **56**, 564-577 (2007).

21 Howe, K., Bateman, A. & Durbin, R. QuickTree: building huge Neighbour-Joining trees of protein sequences. *Bioinformatics* **18**, 1546-1547 (2002).

22 De Bie, T., Cristianini, N., Demuth, J. P. & Hahn, M. W. CAFE: a computational tool for the study of gene family evolution. *Bioinformatics* **22**, 1269-1271 (2006).

23 Kim, B.-M. *et al.* Antarctic blackfin icefish genome reveals adaptations to extreme environments. *Nature ecology & evolution* **3**, 469 (2019).

24 Wang, K. *et al.* Morphology and genome of a snailfish from the Mariana Trench provide insights into deep-sea adaptation. *Nature ecology & evolution* **3**, 823 (2019).

25 Niimura, Y. On the origin and evolution of vertebrate olfactory receptor genes: comparative genome analysis among 23 chordate species. *Genome Biology and Evolution* **1**, 34-44 (2009).

26 Cao, L. *et al.* Neofunctionalization of zona pellucida proteins enhances freeze-prevention in the eggs of Antarctic notothenioids. *Nature communications* **7**, 12987 (2016).

27 Camacho, C. *et al.* BLAST+: architecture and applications. *BMC bioinformatics* **10**, 421 (2009).

28 Birney, E., Clamp, M. & Durbin, R. GeneWise and genomewise. *Genome research* **14**, 988-995 (2004).

29 Meyer, A. & Van de Peer, Y. From 2R to 3R: evidence for a fish‐specific genome duplication (FSGD). *Bioessays* **27**, 937-945 (2005).

30 Amores, A. *et al.* Zebrafish hox clusters and vertebrate genome evolution. *Science* **282**, 1711-1714 (1998).

31 Mungpakdee, S. *et al.* Differential evolution of the 13 Atlantic salmon Hox clusters. *Mol Biol Evol* **25**, 1333-1343, doi:10.1093/molbev/msn097 (2008).

32 Martin, K. J. & Holland, P. W. Enigmatic orthology relationships between Hox clusters of the African butterfly fish and other teleosts following ancient whole-genome duplication. *Mol Biol Evol* **31**, 2592-2611, doi:10.1093/molbev/msu202 (2014).

33 Kuraku, S. & Meyer, A. The evolution and maintenance of Hox gene clusters in vertebrates and the teleost-specific genome duplication. *Int J Dev Biol* **53**, 765-773, doi:10.1387/ijdb.072533km (2009).

34 Woltering, J. M. & Durston, A. J. The zebrafish hoxDb cluster has been reduced to a single microRNA. *Nat Genet* **38**, 601-602, doi:10.1038/ng0606-601 (2006).
35 McClintock, J. M., Kheirbek, M. A. & Prince, V. E. Knockdown of duplicated zebrafish hoxb1 genes reveals distinct roles in hindbrain patterning and a novel mechanism of duplicate gene retention. *Development* **129**, 2339-2354 (2002).
36 Takamatsu, N. *et al.* Duplicated Abd-B class genes in medaka hoxAa and hoxAb clusters exhibit differential expression patterns in pectoral fin buds. *Dev Genes Evol* **217**, 263-273, doi:10.1007/s00427-007-0137-4 (2007).
37 Crow, K. D., Smith, C. D., Cheng, J.-F., Wagner, G. P. & Amemiya, C. T. An independent genome duplication inferred from Hox paralogs in the American paddlefish—a representative basal ray-finned fish and important comparative reference. *Genome biology and evolution* **4**, 937-953 (2012).
38 Montavon, T. *et al.* A regulatory archipelago controls Hox genes transcription in digits. *Cell* **147**, 1132-1145, doi:10.1016/j.cell.2011.10.023 (2011).
39 Beccari, L. *et al.* A role for HOX13 proteins in the regulatory switch between TADs at the HoxD locus. *Genes Dev* **30**, 1172-1186, doi:10.1101/gad.281055.116 (2016).
40 Woltering, J. M., Noordermeer, D., Leleu, M. & Duboule, D. Conservation and divergence of regulatory strategies at Hox Loci and the origin of tetrapod digits. *PLoS Biol* **12**, e1001773, doi:10.1371/journal.pbio.1001773 (2014).
41 Braasch, I. *et al.* The spotted gar genome illuminates vertebrate evolution and facilitates human-teleost comparisons. *Nat Genet* **48**, 427-437, doi:10.1038/ng.3526 (2016).
42 Schartl, M. *et al.* The genome of the platyfish, Xiphophorus maculatus, provides insights into evolutionary adaptation and several complex traits. *Nature genetics* **45**, 567 (2013).
43 Roux, J., Liu, J. & Robinson-Rechavi, M. Selective constraints on coding sequences of nervous system genes are a major determinant of duplicate gene retention in vertebrates. *Molecular biology and evolution* **34**, 2773-2791 (2017).
44 Bayés, A. *et al.* Evolution of complexity in the zebrafish synapse proteome. *Nature communications* **8**, 14613 (2017).

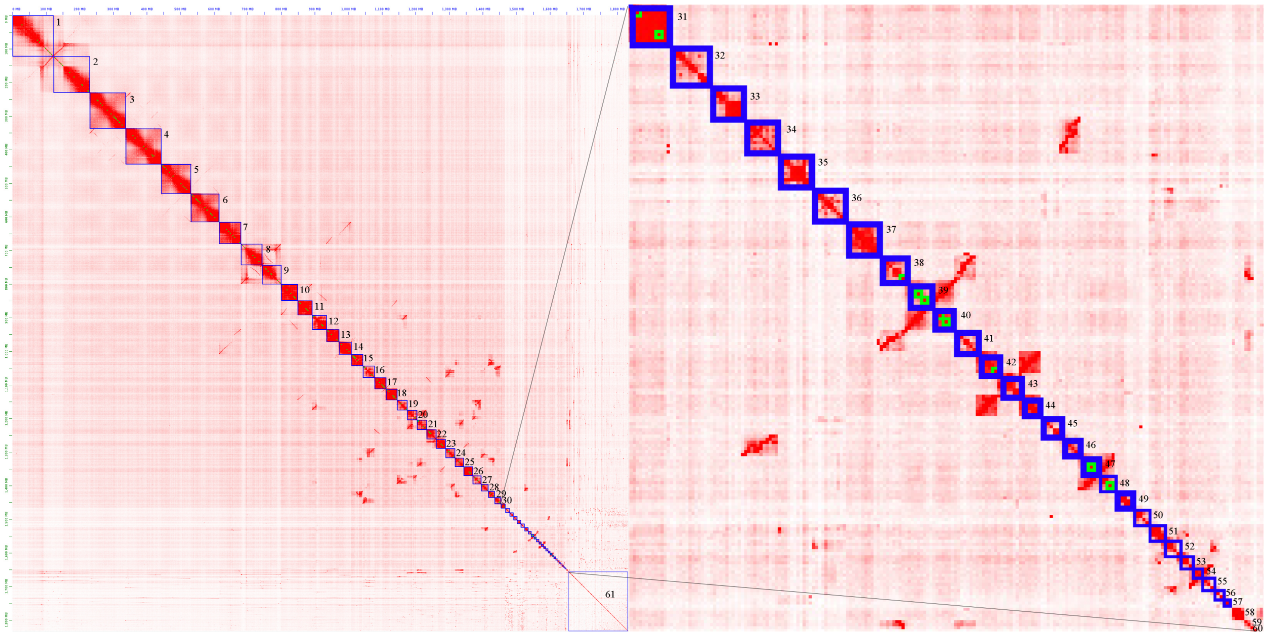

Supplementary Fig. 1. Heatmap of interactions within and among chromosomes according to Hi-C analysis. Chromosomes size scaffolds are indicated by the blue frames and numbered according to size.

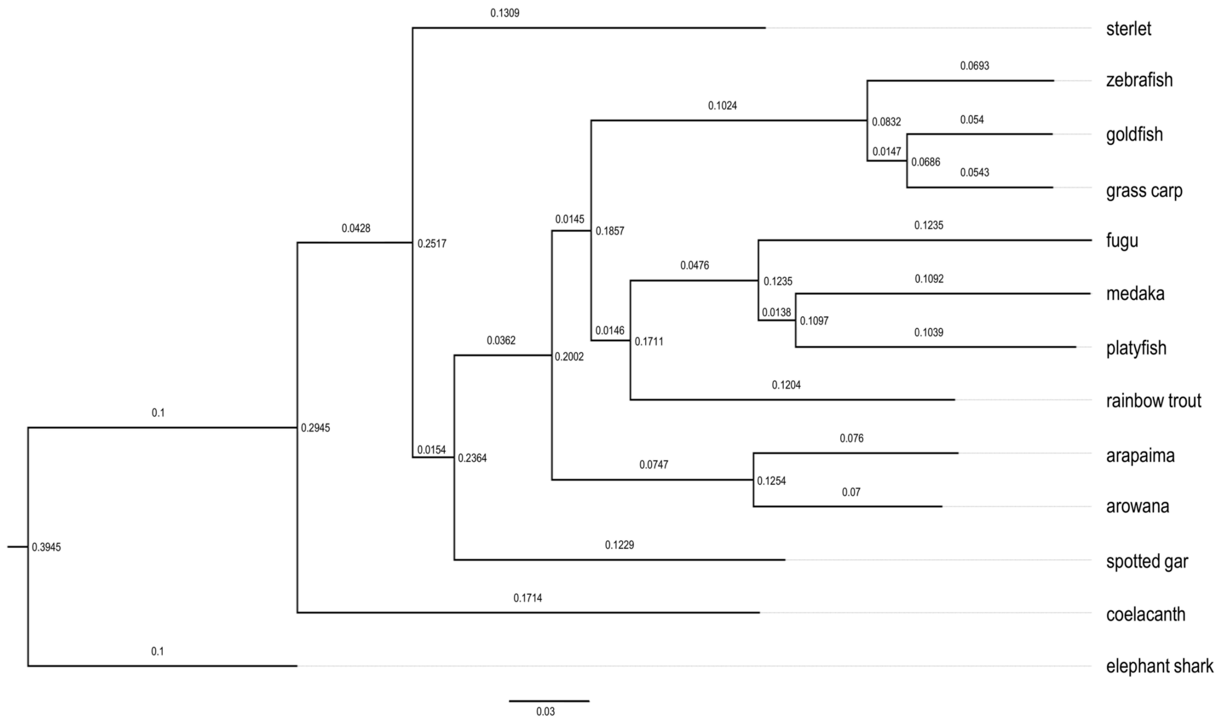

Supplementary Fig. 2. phylogenetic tree drawn by the interactive Tree Of Life tool (iTOL, https://itol.embl.de/)) with default settings based on all homologes resulting from comparison2 (positive selection analysis). Numbers on the branch indicate branch length. Bar represents 0.03 substitutions per site.

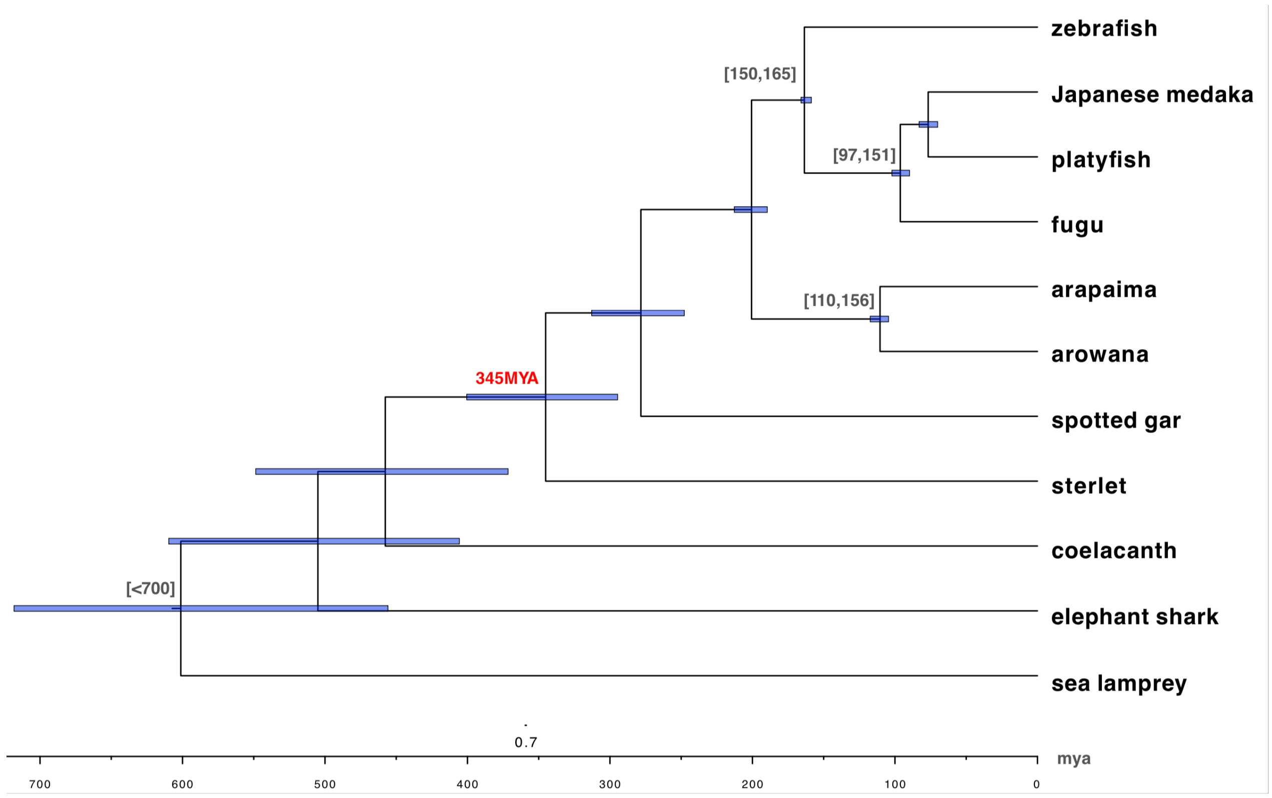

Supplementary Fig. 3. Divergence time of sterlet. The timescale was calculated from a phylogenetic tree based on 275 one-to-one orthologs using MCMCtree. Branch lengths were calibrated by using the fossil records for the split of medaka/fugu, zebrafish/stickleback, arapaima/arowana and sea lamprey. Numbers in black brackets indicate MYA of the fossil calibrations. Blue bars refer to the 95% confidence interval. Red numbers indicates the estimated time of sterlet divergence 345 MYA (295 - 400, 95% confidence level).

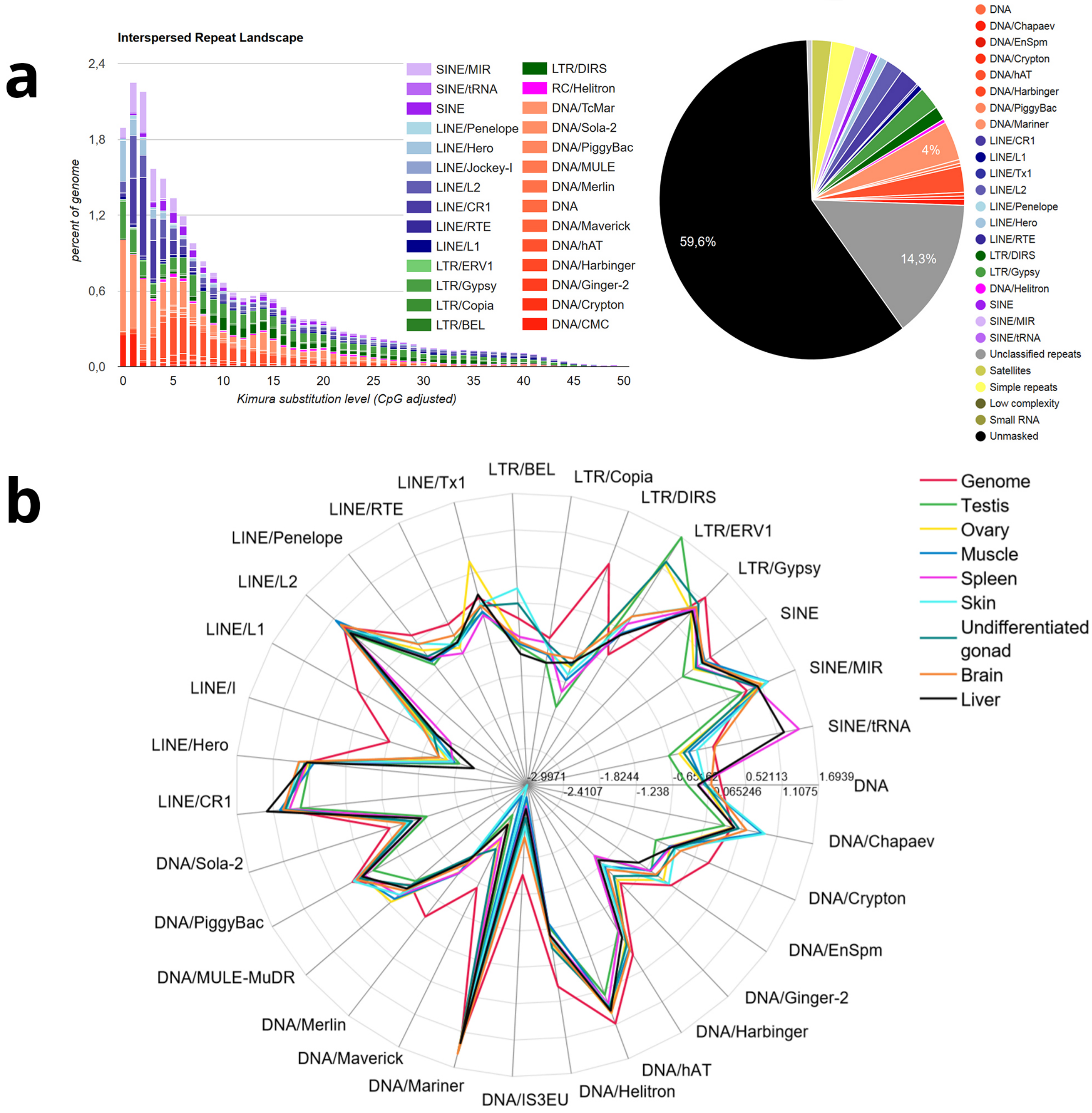

Supplementary Fig. 4. Evolutionary history and expression of TEs. a) Copy-divergence analysis of TE classes in sterlet, based on Kimura 2 parameter distances. The percentages of TEs in genomes (y axis) are clustered based on their Kimura values (x axis; K values from 0 to 50; arbitrary values). Older copies are located on the right side of the graphs while recent copies are located on the left side. b) The proportion of TE superfamily representation in the genome and eight organ transcriptomes of sterlet. The proportion of eachTE superfamily was initially calculated as (% of TE superfamily Å~ 100) / total % of TEs in the genome or transcriptome, and then for the spider graph transformed to log10 values. The expression of LTR/ERV1 elements in gonads and SINE/tRNA in liver and spleen might be the result of their activity rather than of general background expression because their relative fraction is notably higher in the transcriptome than in the genome.

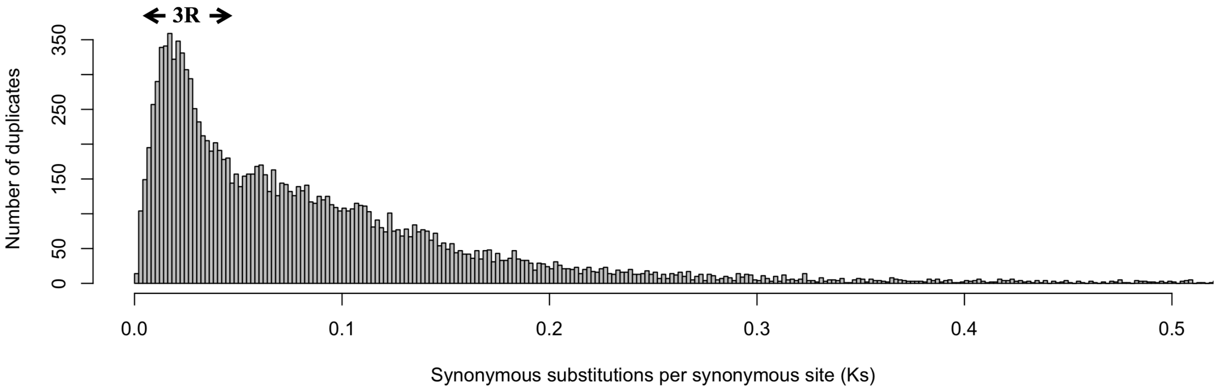

Supplementary Fig. 5. Age distribution of the sterlet paranome based on Ks values . The 3R event is obvious and indicated, while there is no visible signal from the 2R and 1R WGDs probably due to their very ancient occurrence.

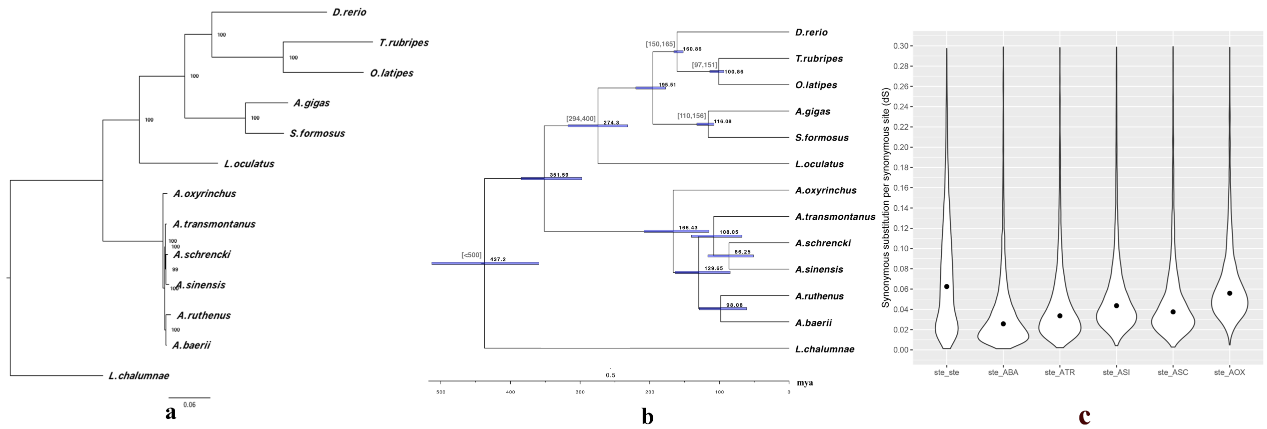

Supplementary Fig. 6. Estimation of sterlet WGD age. a) Phylogenetic tree showing the divergence of protein sequence among species b) Chronogram showing the divergence times of sturgeons and Teleos with L. chalumnae as out group. Divergence time were calibrated by using the fossil records for the split of medaka/fugu, zebrafish/stickleback, arapaima/arowana, and inferred time for gar/sterlet and coelacanth (the root). Numbers in black brackets indicate MYA of the calibrations. Blue bars refer to the 95% confidence interval. c) Violinplot comparing the distribution of pairwise dS among orthologous pairs between sterlet and *A. baerii* (ste_ABA); sterlet and *A. transmontanus* (ste_ATR); sterlet and *A. schrencki* (ste_ASC); sterlet and *A. sinensis* (ste_ASI); sterlet and *A. oxyrinchus* (ste_AOX); and between sterlet ohnolog pairs (ste_ste). Pairwise dS was calculated using codeml (PAML 4.9, runmodel=-2).

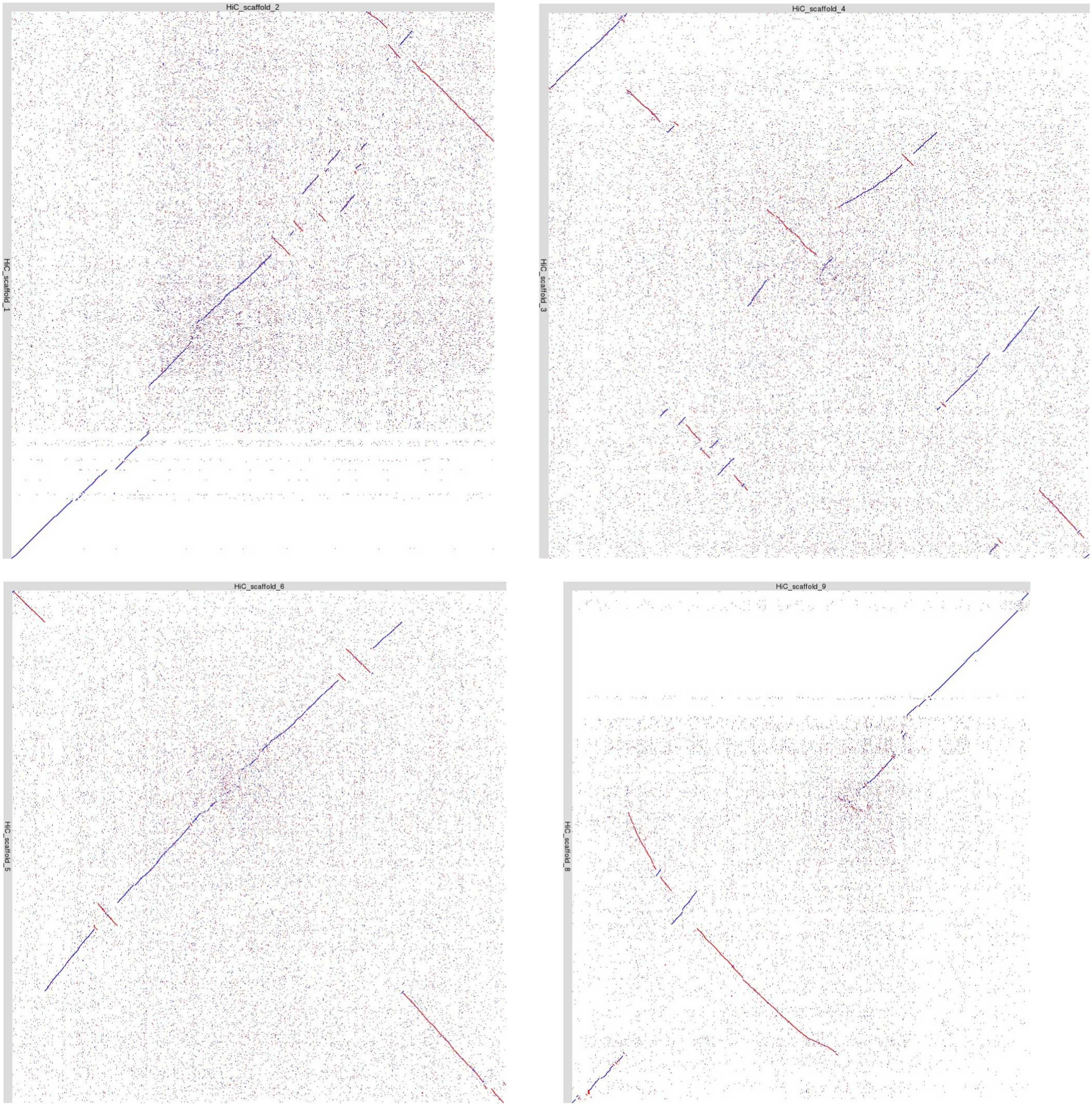

Supplementary Fig. 7. Dotplots showing sequence alignments between sterlet chromosomes 1 and 2 (upper left), 3 and 4 (upper right), 5 and 6 (bottom left), 8 and 9 (bottom right).

Corresponding chromosomes were aligned using LAST. Alignments with error probability > 10e-8 were discarded. The long homologous regions imply gene synteny and conservation of the gene order.

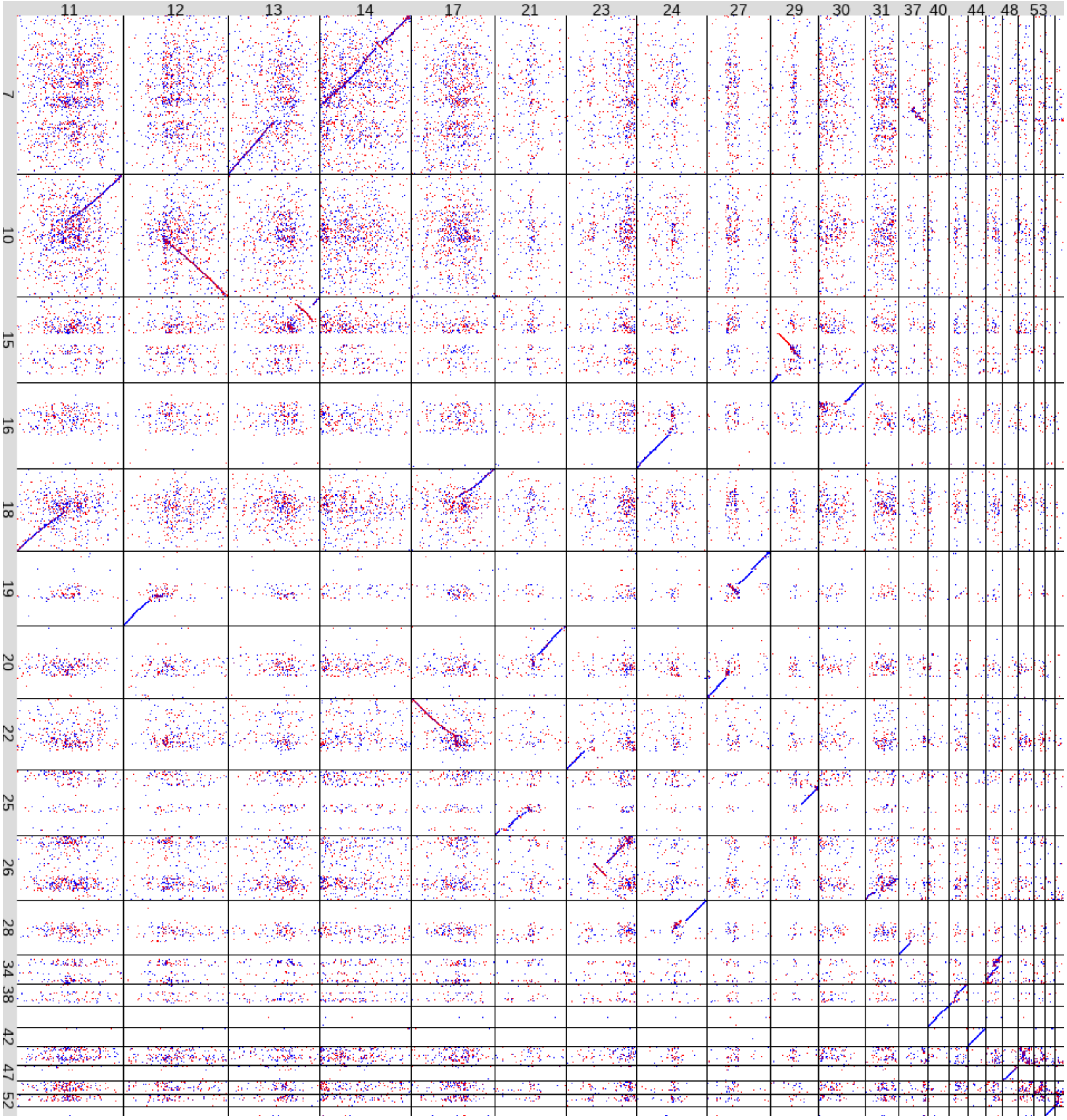

Supplementary Fig. 8. Dotplots showing sequence alignments among sterlet chromosomes 7, 10-31, 34, 37-44, 46-49 and 51-56. Corresponding chromosomes were aligned using LAST. Alignments with error probability > 10e-8 were discarded. The long homologous regions imply gene synteny and conservation of the gene order.

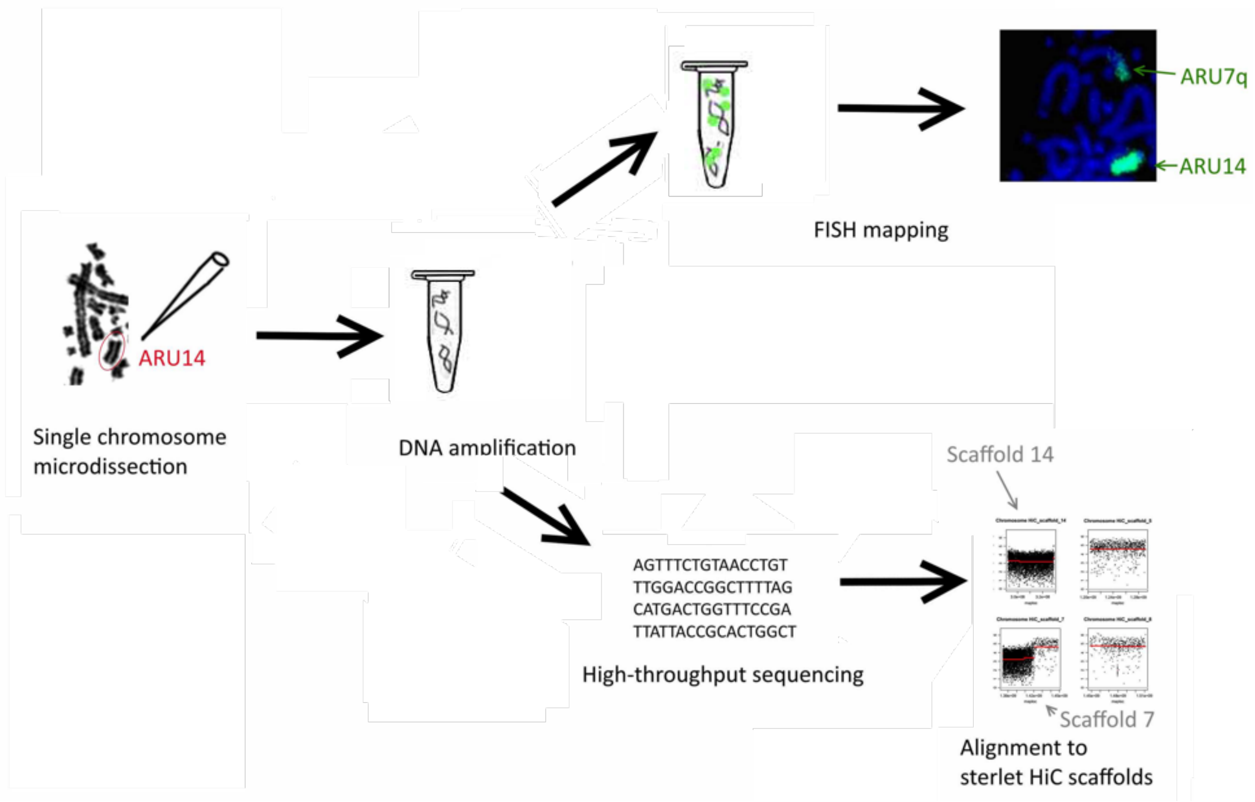

Supplementary Fig. 9. Schematic drawing of the strategy for validation of genome assembly using single chromosome low-coverage sequencing. Paralogous chromosomes are revealed both by FISH (i.e. ARU14 paints both ARU14 and ARU7q) and DOPSeq alignment to sterlet sca!olds (ARU14 library reveals strong signals on sca!olds 14 and 7q).

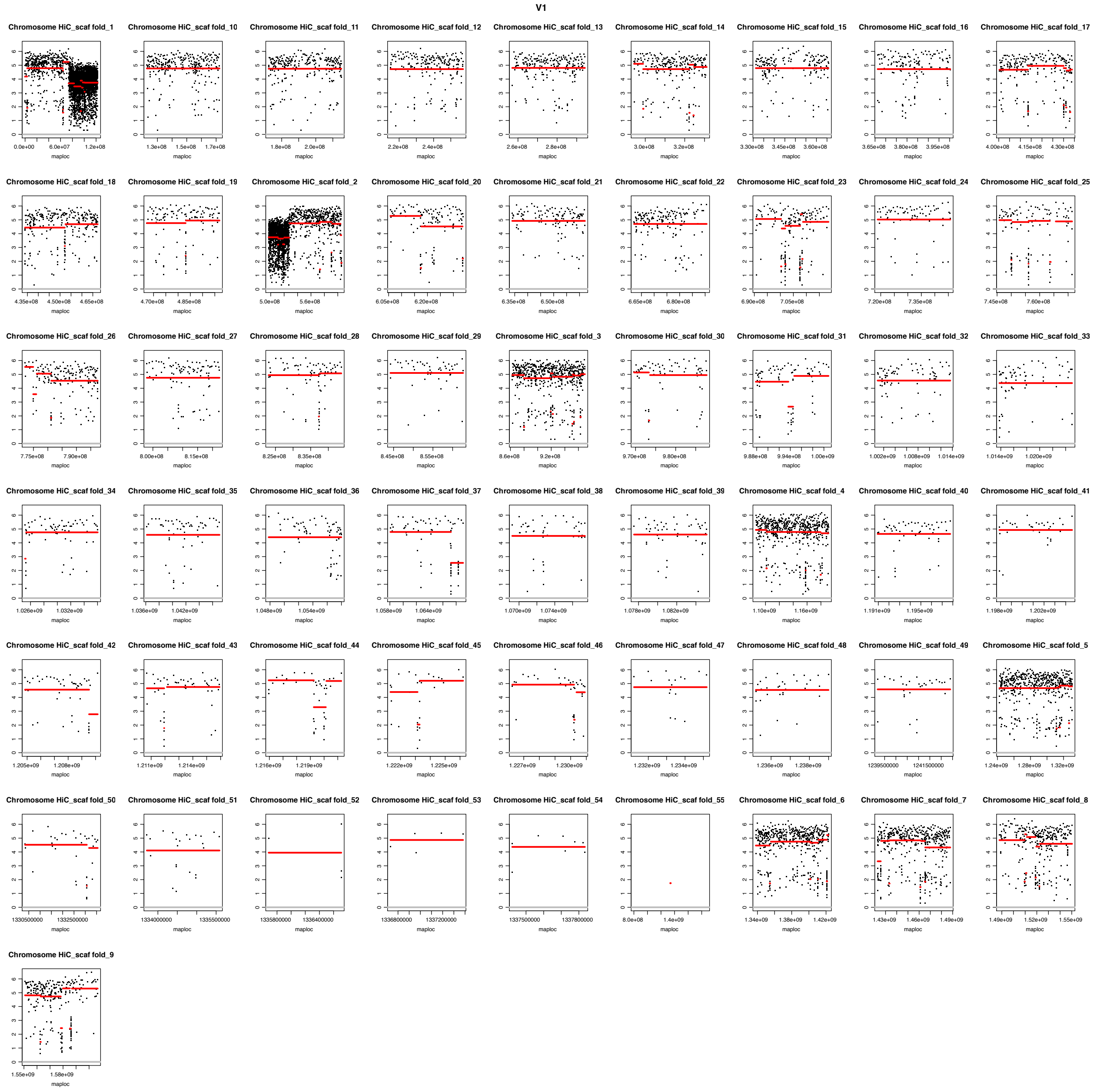

Supplementary Fig. 10. Mapping blots of Aru1p library on sterlet assemblies

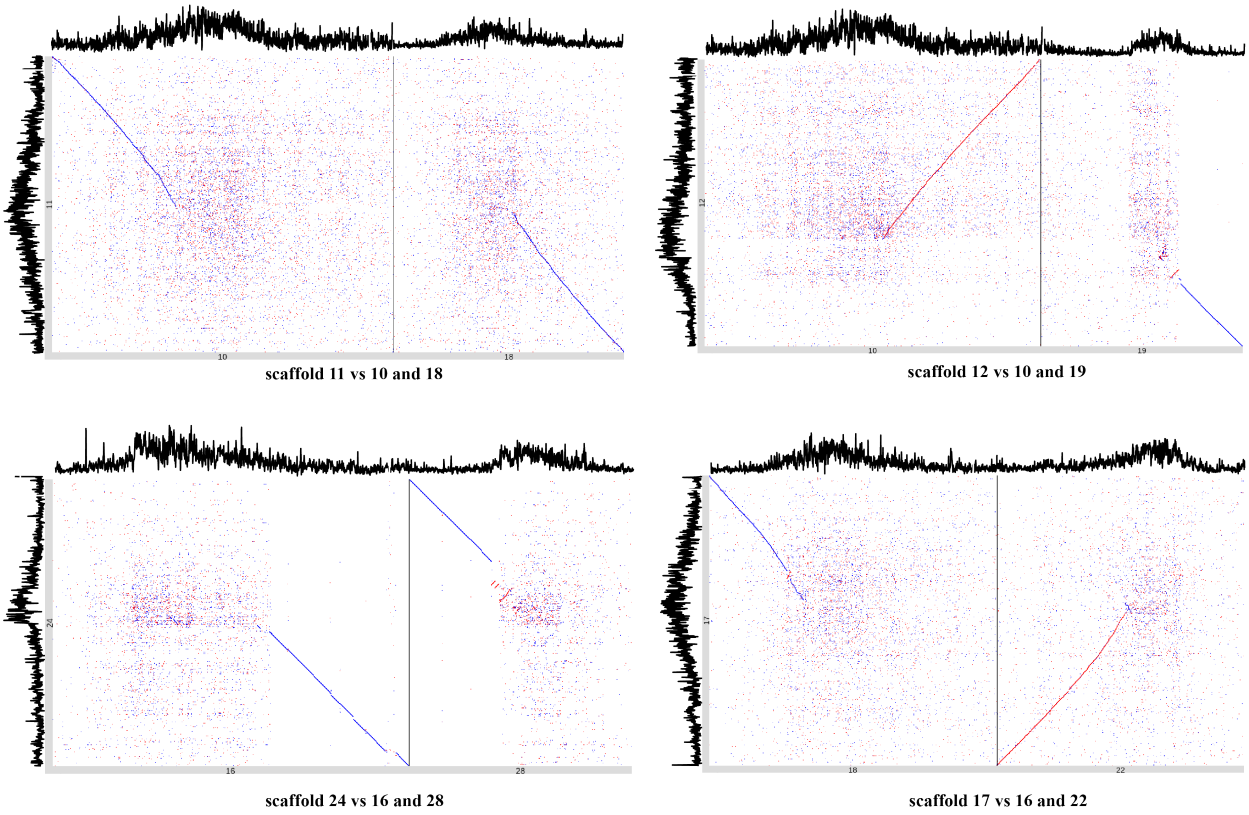

Supplementary Fig. 11. Dotplot showing sequence alignments and line charts revealing the content of repeat elements. Corresponding chromosomes were aligned using LAST. Alignments with error probability >10e-8 were discarded. The line chart on the left and top of each dotplot represents the percentage of repeat elements of corresponding sequence regions (window size 30k).

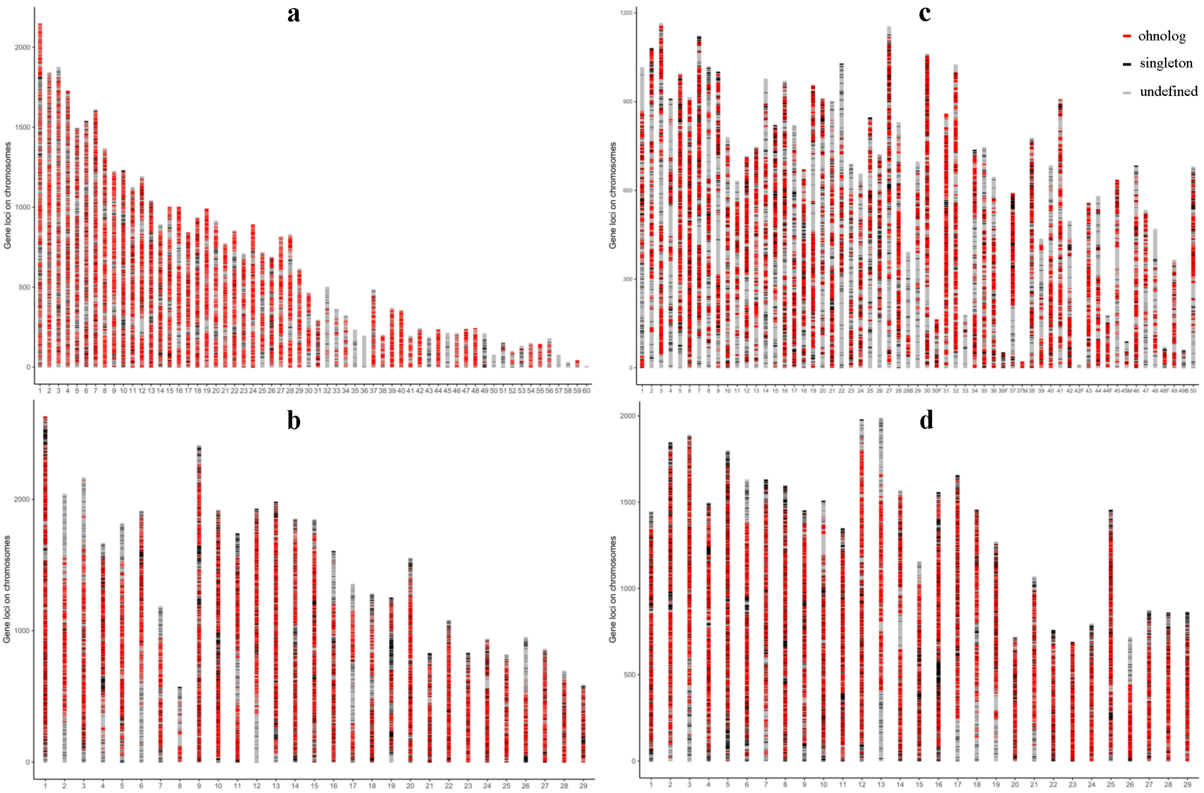

Supplementary Fig. 12. Location of singletons and ohnologs on chromosomes of sterlet (a), goldfish (b), Atlantic salmon (c) and rainbow trout (d). Red bars represent ohnologs, black are singletons and grey is for undefined.

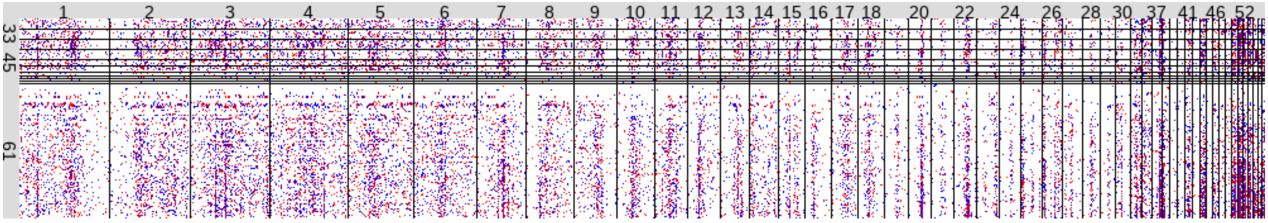

Supplementary Fig. 13. Dotplot showing sequence alignments of sterlet chromosomes 32, 33, 35, 36, 45, 50, 57-60 and unassigned (scaffold 61) to the reset of genome. Corresponding chromosomes were aligned using LAST. Alignments with error probability > 10e-8 were discarded. The plot reveals no linear alignment of sterlet chromosome 32-35, 37-55 and U to the other chromosomes, indicating they have lost their homeologous counterparts during rediploidization.

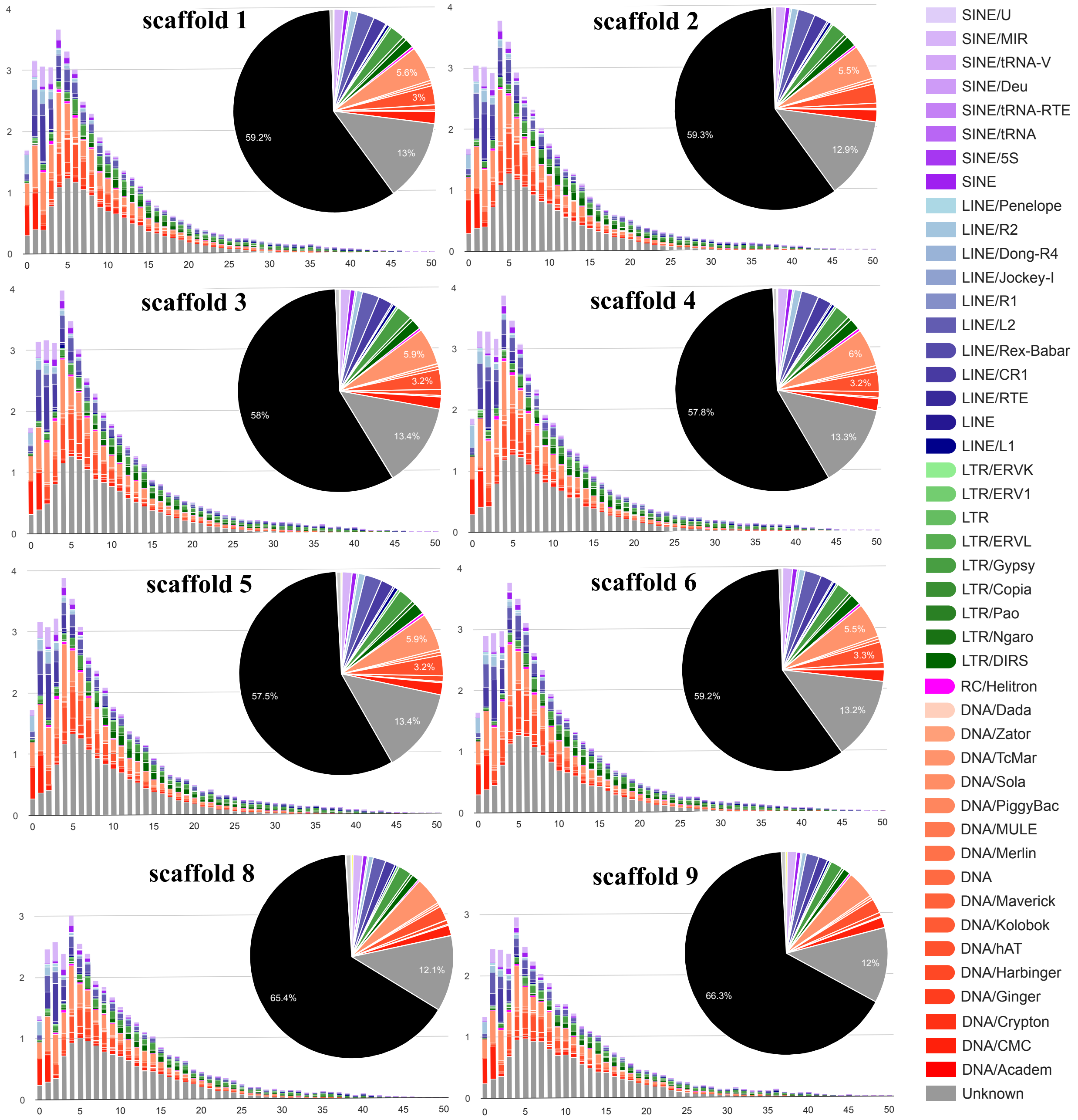

Supplementary Fig. 14. Similarity of Kimura-landscape of repeat elements revealing autopolyploidy of sterlet. Comparison of Kimura-landscape of repeat elements between the homeologous scaffold pairs 1-2, 3-4, 5-6 and 8-9. Percentages of repeats (Y-axis) are clustered based on their Kimura values (X-axis), which are arbitrary values calculated from nuclear divergence. Left side of X-axis represents recent copies while those on the right side are more ancient.

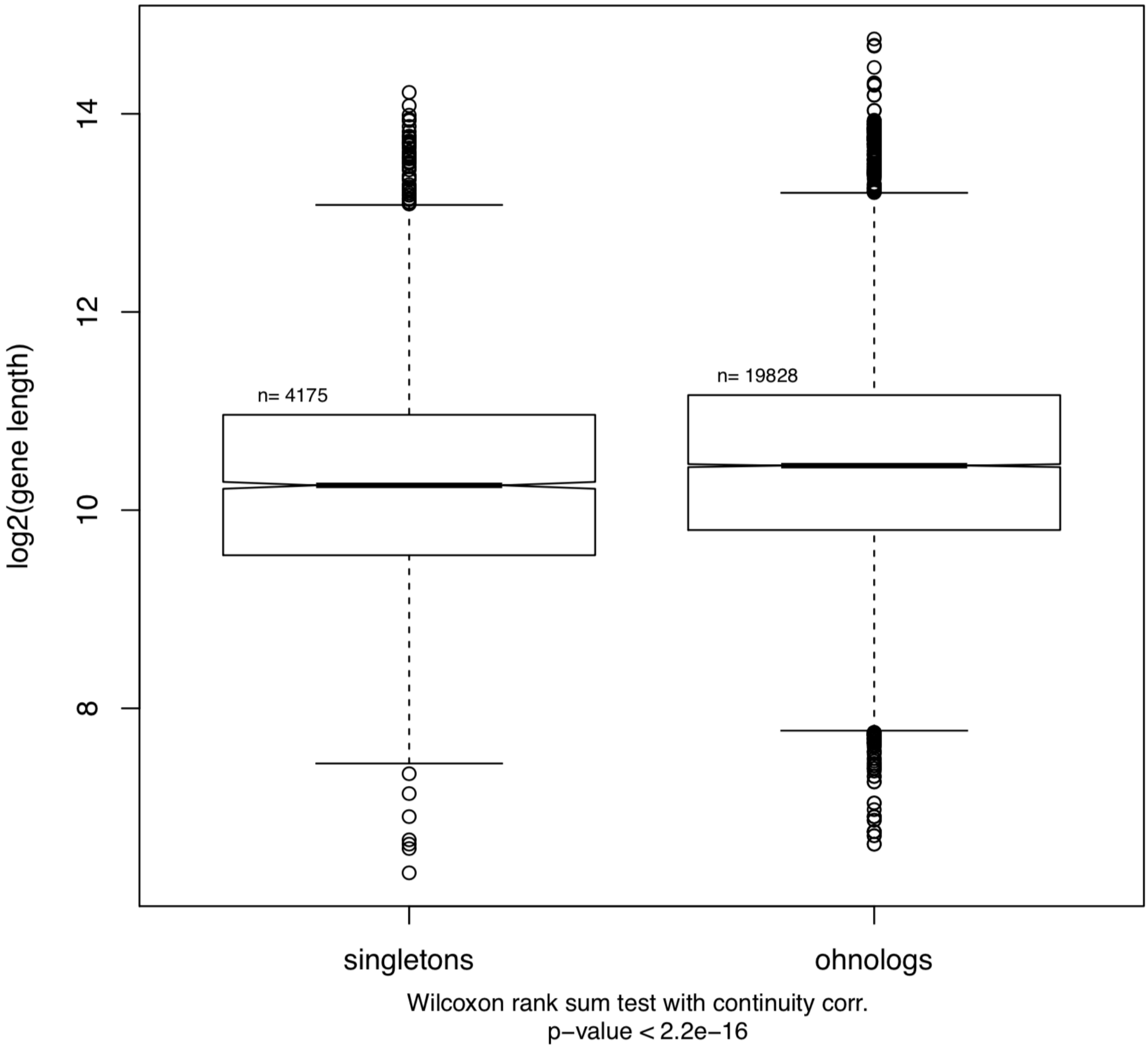

Supplementary Fig. 15. Boxplot of log2(gene lengths) for singletons and ohnologs.

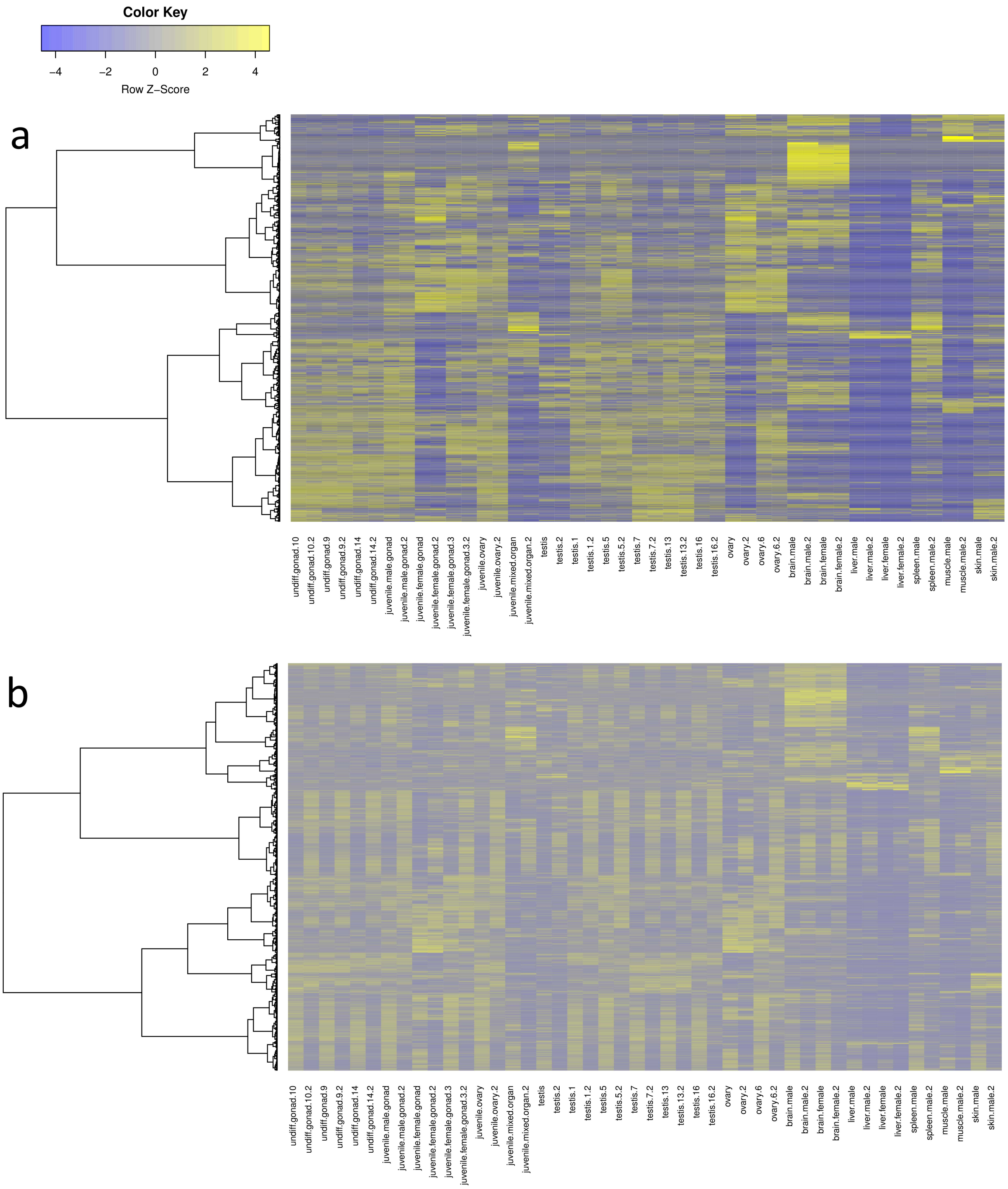

Supplementary Fig. 16. Heatmaps of genes equally expressed (a) or differentially expressed between ohnologs in at least two samples (b). Only expressed ohnologs were considered (TPM>5 in at least one sample). Ohnologes were considered to be different expression levels, if the value for one onolog was at least twice the value for the second onolog in at least two samples. Heatmap color displays the z-score of log2TPM+1 ranging from blue (low expression) to yellow (high expression). Columns represent individual samples, while rows represent genes. The values for both ohnologs are plotted in adjacent columns with '.2' denoting the gene values in the same sample for the second ohnolog.

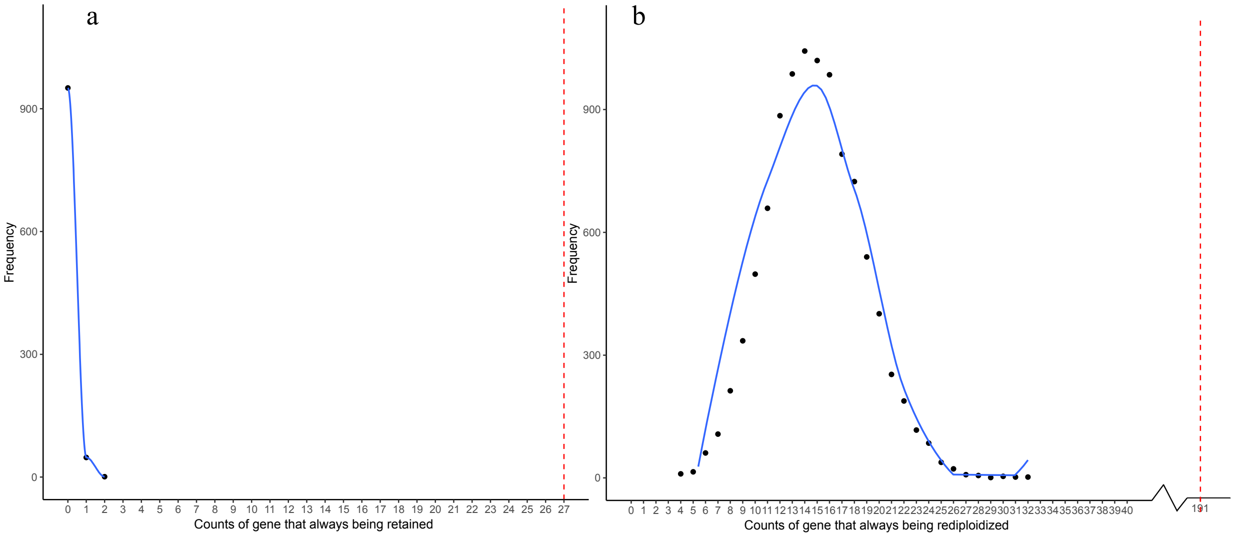

Supplementary Fig. 17. Dotplot of expected number of genes that are always retained (a) or deduplicated (b) after WGDs under random retaining/deduplication process. Starting from 15216 genes, we simulated a stochastic process by randomly retaining or deduplicating the ohnologs after each WGD in sterlet, arapaima, zebrafish, goldfish, medaka, Atlantic salmon and rainbow trout. For each of the 10,000 simulations, the genes that were always deduplicated or retained were counted. The dashed red vertical line indicates the count observed.

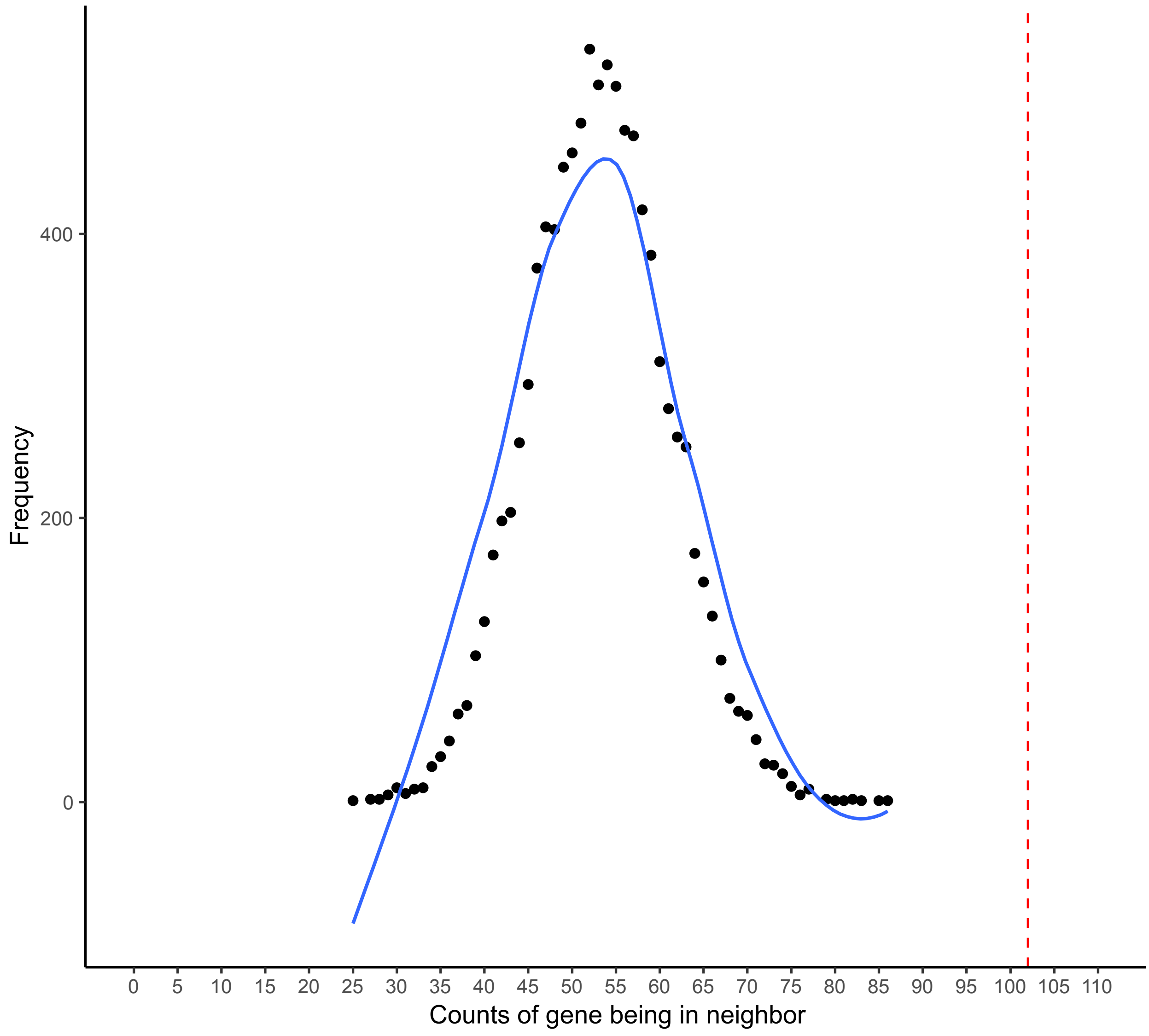

Supplementary Fig. 18. Dotplot of expected gene counts for close linkage under a random rediploidization process. From 15216 gar genes, we randomly resampled, with no return, 191 genes (the number of observed genes always being deduplicated after WGDs) to count the genes neighbouring each other (with in between not more than 5 genes missing). We repeated the resampling for 10,000 times for the expectation distribution. The dashed red vertical line indicates the count observed.

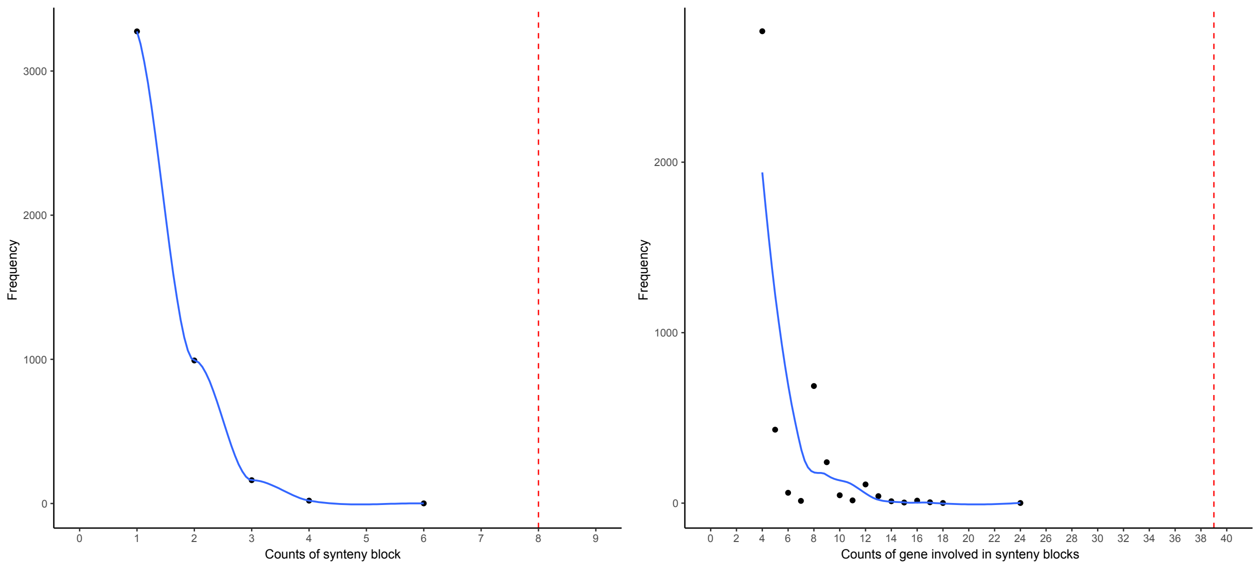

Supplementary Fig. 19. Dotplot of expected synteny block counts and number of genes involved under random rediploidization process. From 15216 gar genes, we randomly resampled, with no return, 191 genes (the number of observed genes always being deduplicated after WGDs) to count the synteny blocks (containing five genes at least, with gap <15) and the number of genes in blocks. We repeated the resampling for 10,000 times for the expectation distributions. The dashed red vertical lines indicates the count observed.

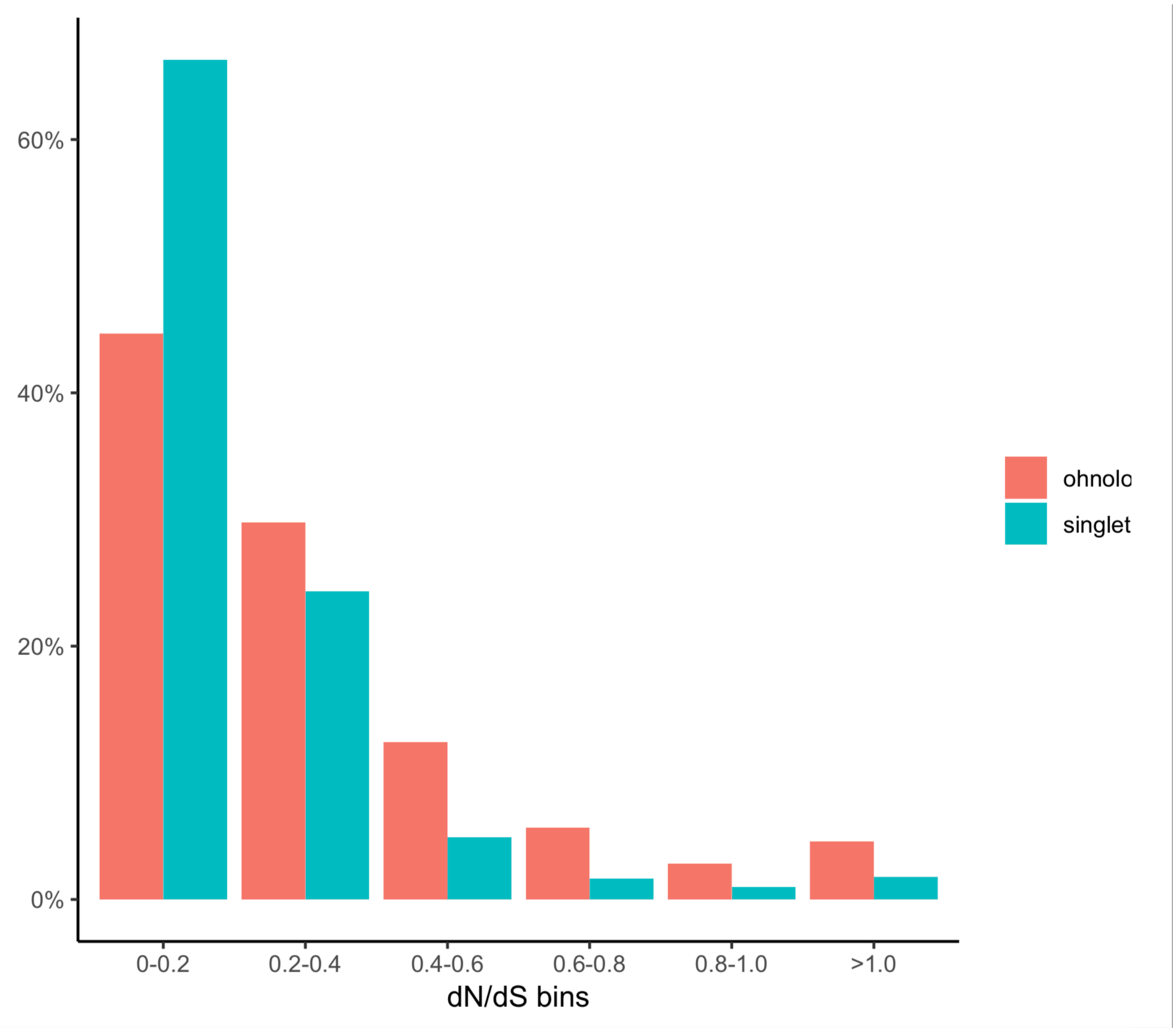

Supplementary Fig. 20. Distribution of omega (dN/dS) values of ohnologs and singletons in sterlet. While singletons have a higher fraction of genes with low omega values than ohnologs, ohnologs are enriched for genes with higher dN/dS values. Omega values were calculated using codeML (PAML4.9) under free-ratio model. For each sterlet singleton or pair of ohnologs their single-copy orthologies in other species were included to reconstruct the multiple alignment and gene tree (guided by species tree).

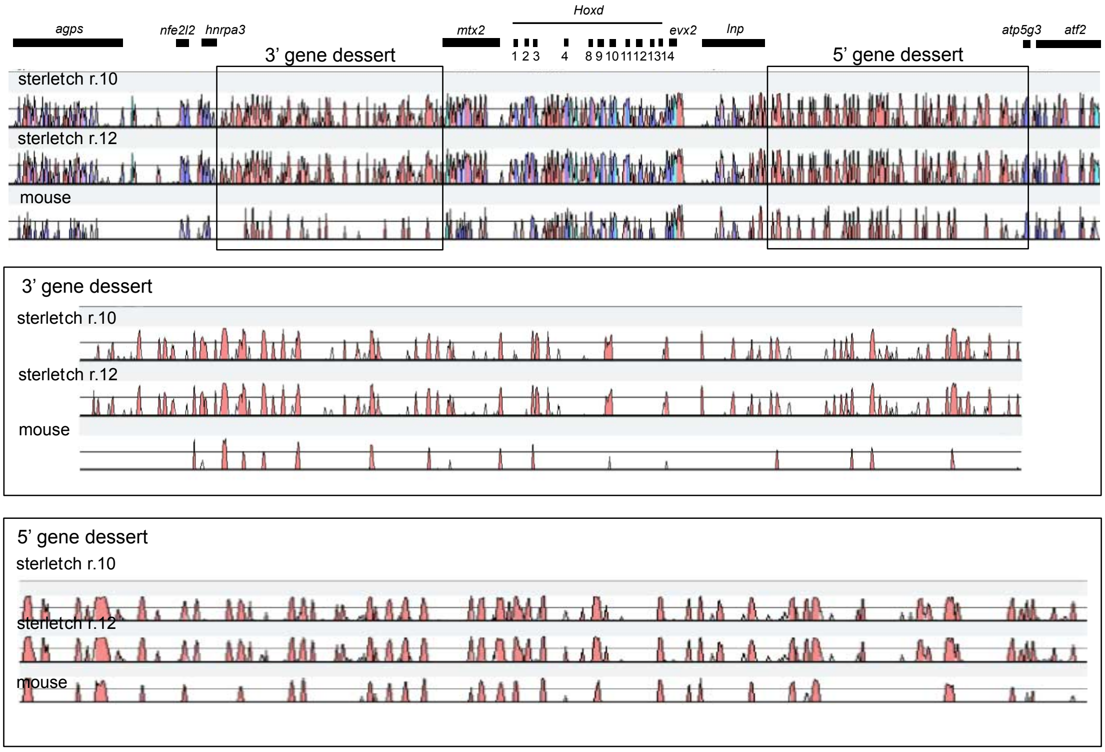

Supplementary Fig. 21. Lagan VISTA plot for the hoxd cluster synteny region from agps to atf2. The spotted gar sequence was used as baseline, shown in comparison with the mouse and the two sterlet Hoxd clusters (from chromosome 10 and chromosome 12 respectively). The gnathostome Hoxd clusters are flanked on either end by gene deserts enriched for ultra conserved non-coding elements (UCNEs) (light red), which are involved in long-range gene regulation. The 3' gene desert is located between hrnp3a and mtx2 and the 5' gene dessert between lnp and atp5g3. The extent of both gene desserts is indicated on the synteny plot in the top panel. Separate enlargements for the 3' and 5' gene desserts are shown in the lower two panels. Both gene deserts are characterised by a large number of UCNEs. The conservation profile for each of the sterlet Hoxd clusters is very similar and all UCNEs shared with the spotted gar are present in both ohnologous synteny regions.

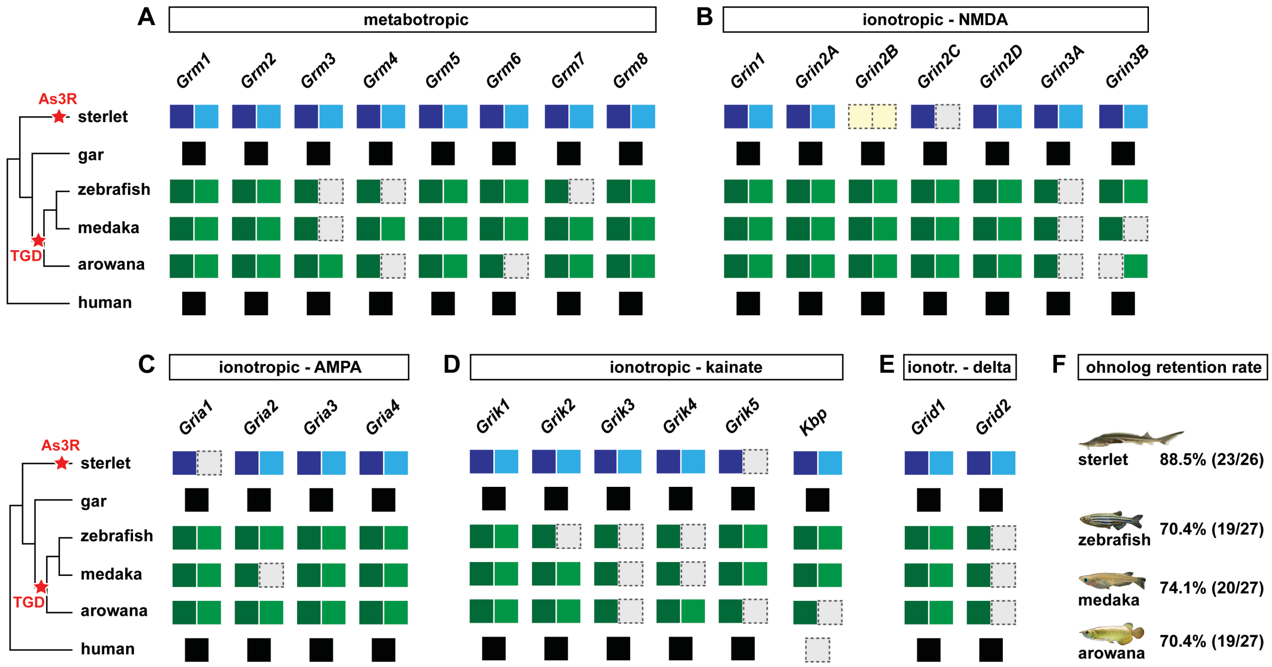

Supplementary Fig. 22. GR gene repertoire in bony vertebrates. Genes are symbolized by filled squares. Ohnologs from the As3R and TGD event are indicated my dark and light blue and green squares, respectively. Squares with dashed lines indicate gene losses.

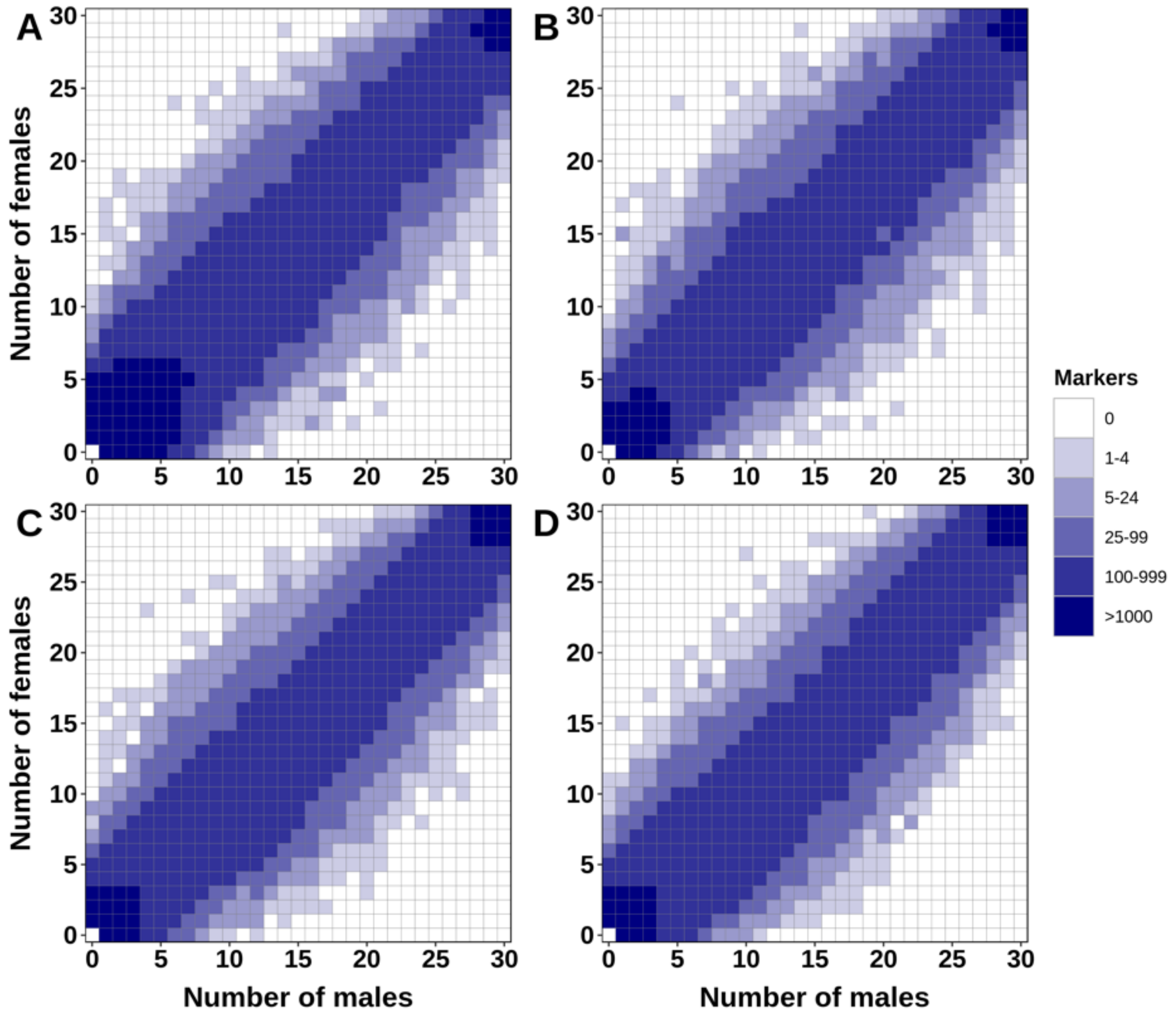

Supplementary Fig. 23. distribution of RADSex markers in males and females for *A. ruthenus*. The distribution of markers in male and female individuals was computed with RADSex with a minimum depth to consider a marker present in an individual of 1 (A), 2 (B), 5 (C), and 10 (D). In each tile plot, the number of males and number of females are represented on the horizontal and vertical axes respectively, and the color of a tile indicates the number of markers present in the corresponding number of males and females. There was no marker associated with phenotypic sex (i.e. markers found in most individuals from one sex and absent from most individuals from the other sex) for any minimum depth value.

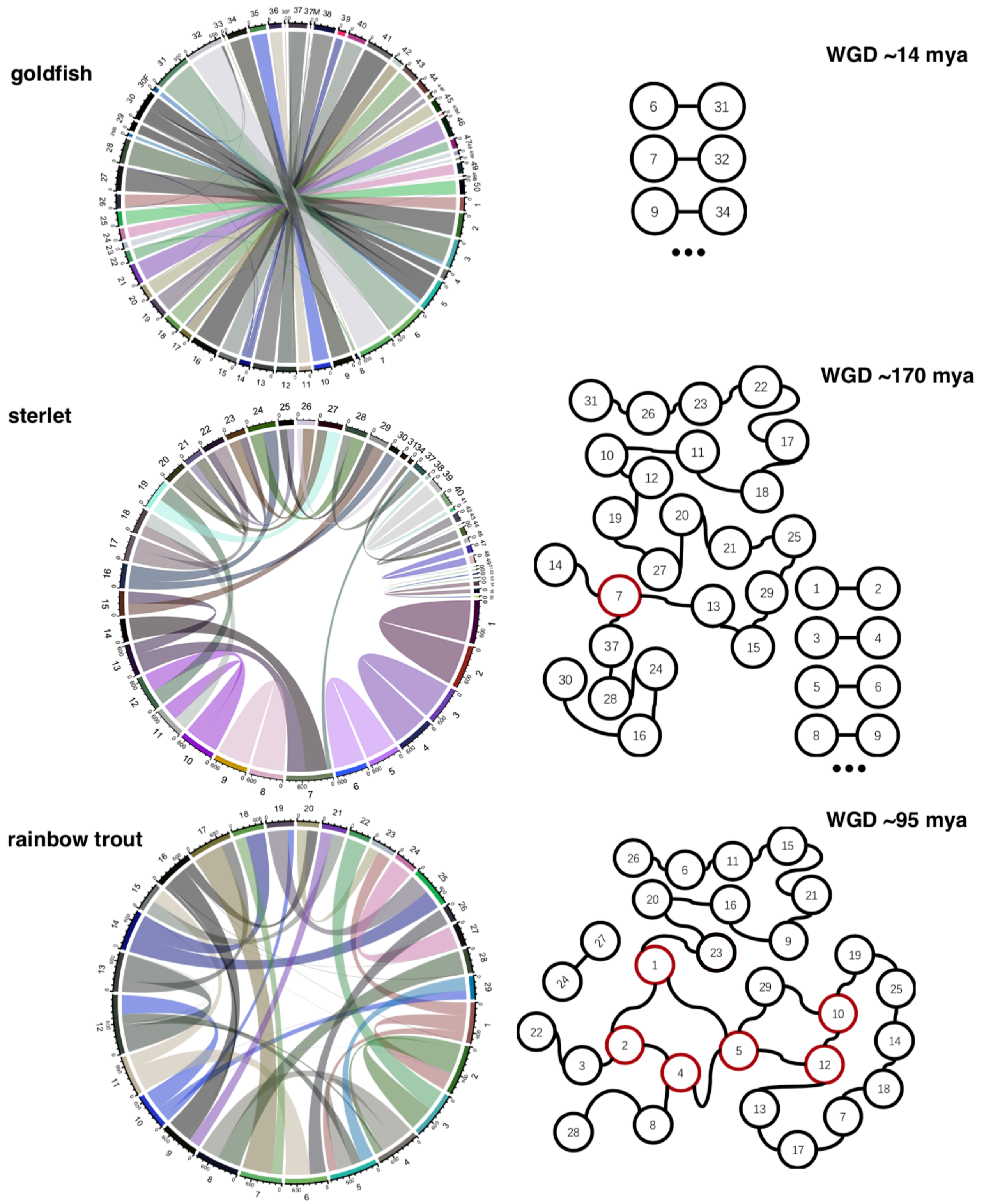

Supplementary Fig. 24. Schematic diagram demonstrating the chromosome dynamics after WGD in goldfish, sterlet and rainbow trout. Left, chord diagrams showing the pairwise homeology relations. Right, schematic representation of homeolog correspondence between whole chromosomes or chromosome arms. In goldfish, all chromosomes are homeologous over the whole length, in sterlet only four chromosome pairs show full correspondence, while in rainbow trout only one such pair is found. The arrangement in the chains is inferring the sequence of chromosome arm exchanges. Hubs are painted in red.

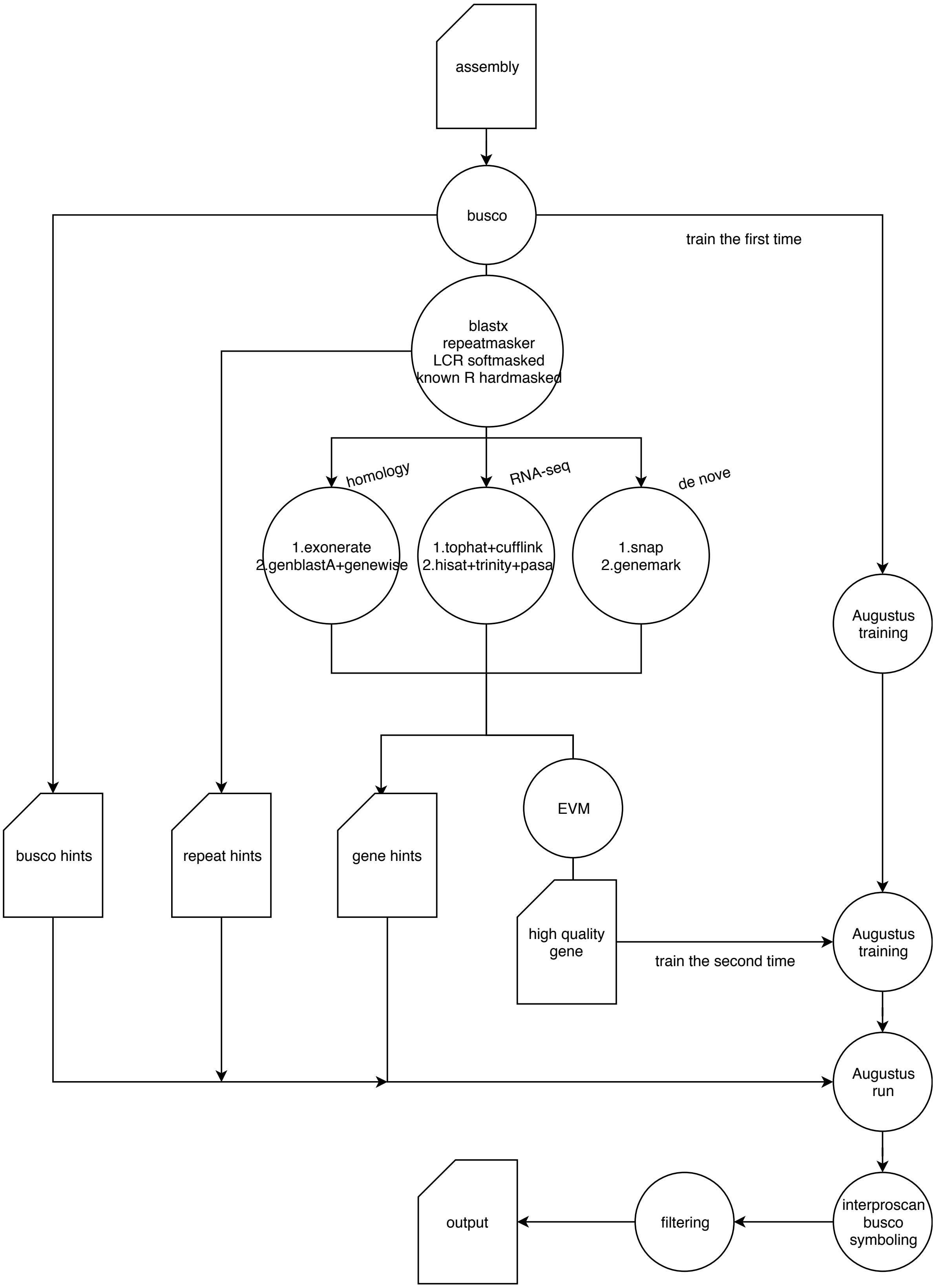

Supplementary Fig. 25. Flowchart of the genome annotation process. For explanation see Materials and methods section.

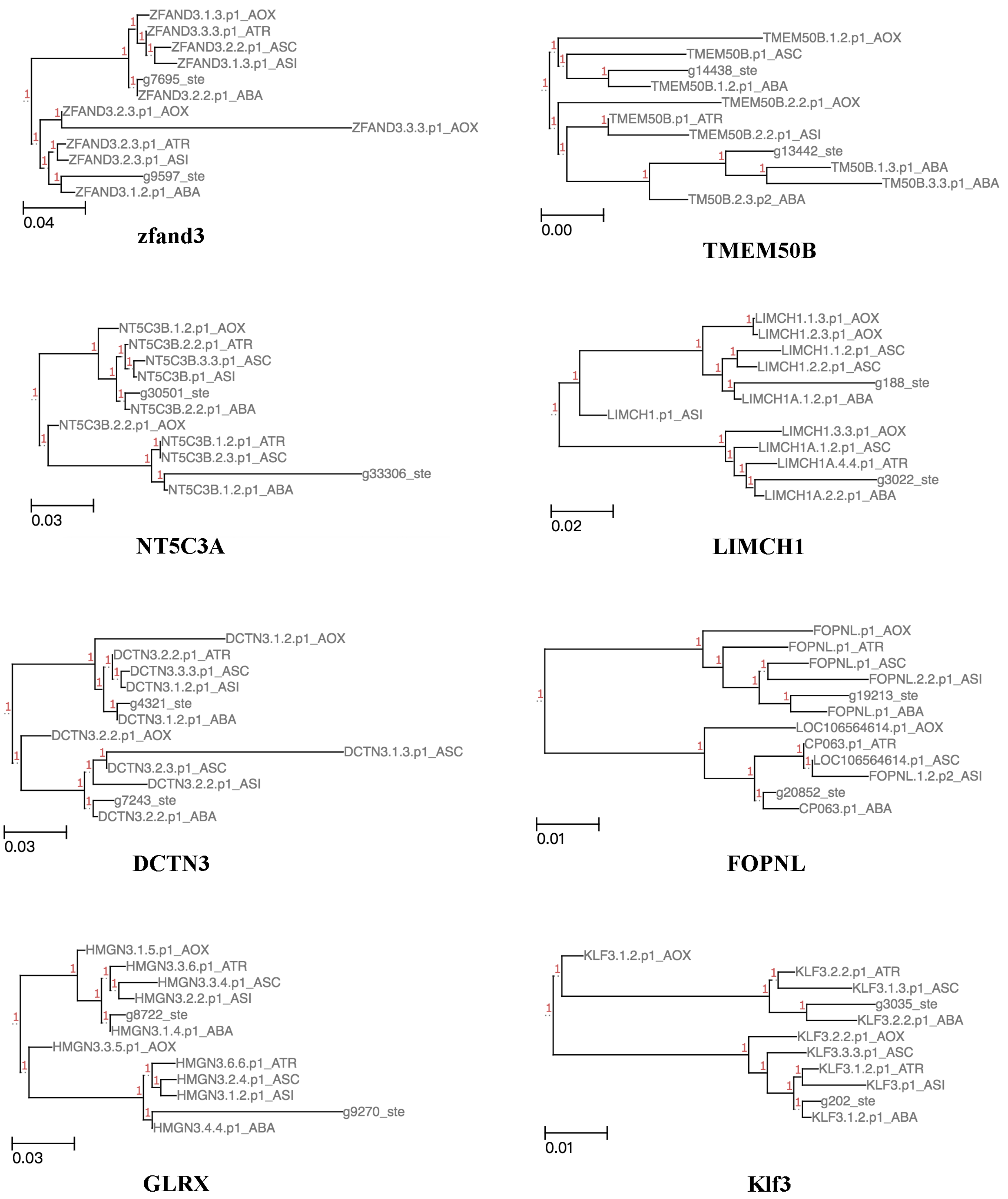

Supplementary Fig. 26. Examples of gene trees indicating the sterlet WGD happened before the sterlet/*A. oxyrinchus* split. Gene trees were constructed using TreeBeST 0.5.1. The last three letters after "_" of each tip refer to species names as follows, "ste" refers to sterlet; "AOX", *A. oxyrinchus*; "ATR", *A. transmontanus*; "ASC", *A. schrencki*; "ASI", *A. sinensis*; and "ABA", *A. baerii*.

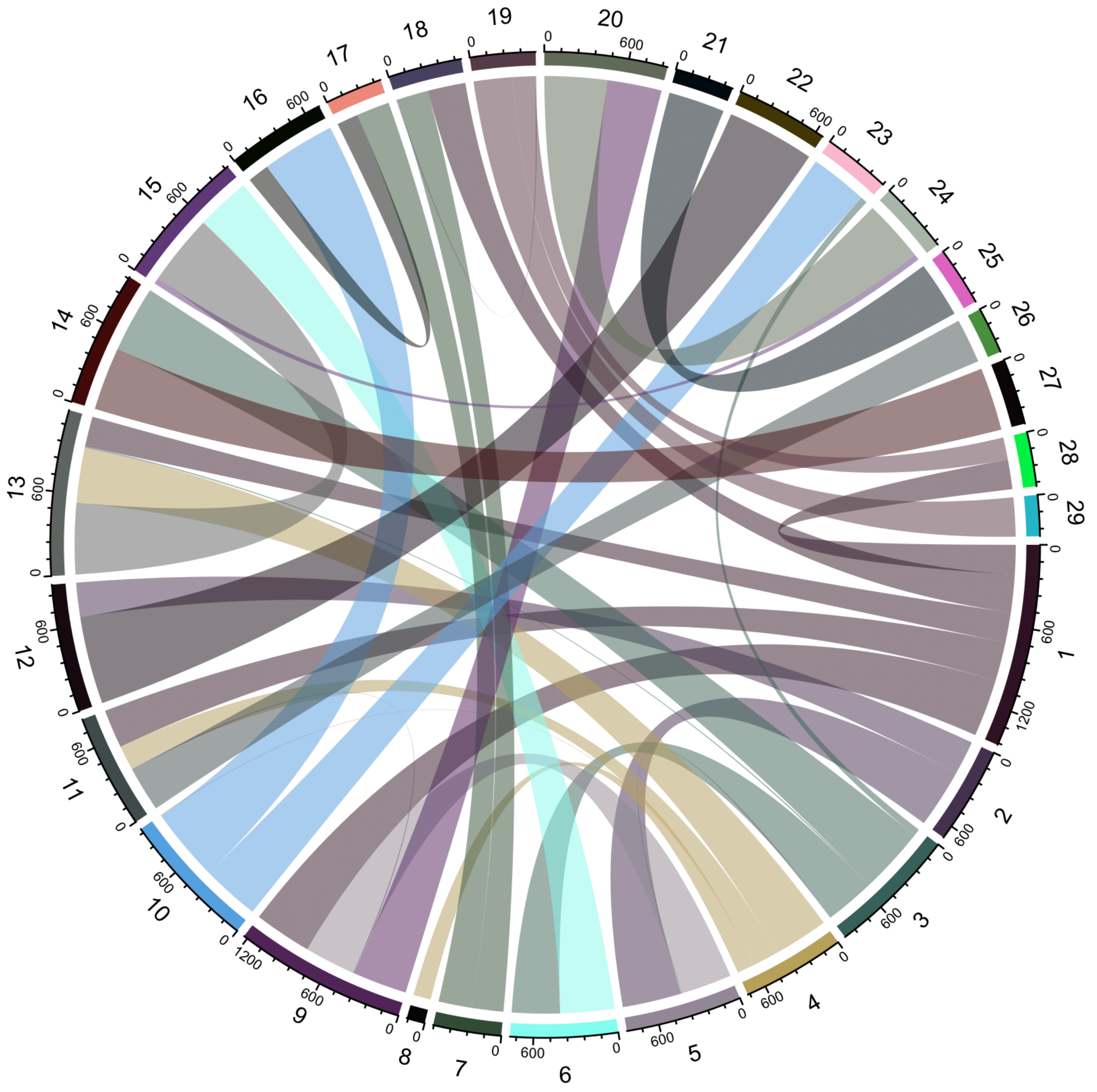

Supplementary Fig. 27. Chord diagram for Atlantic salmon

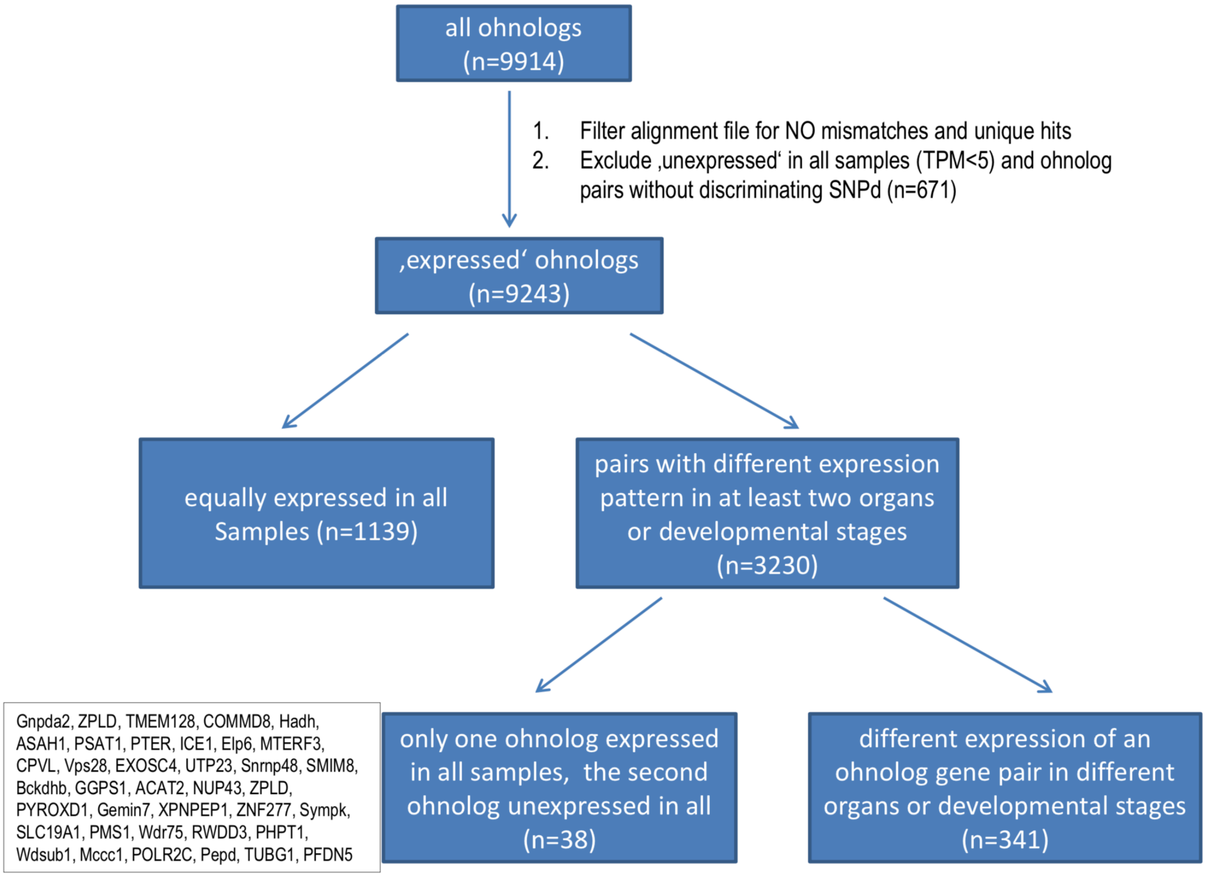

Supplementary Fig. 28. Scheme of ohnolog groups with different expression patterns.

Supplementary Data 1

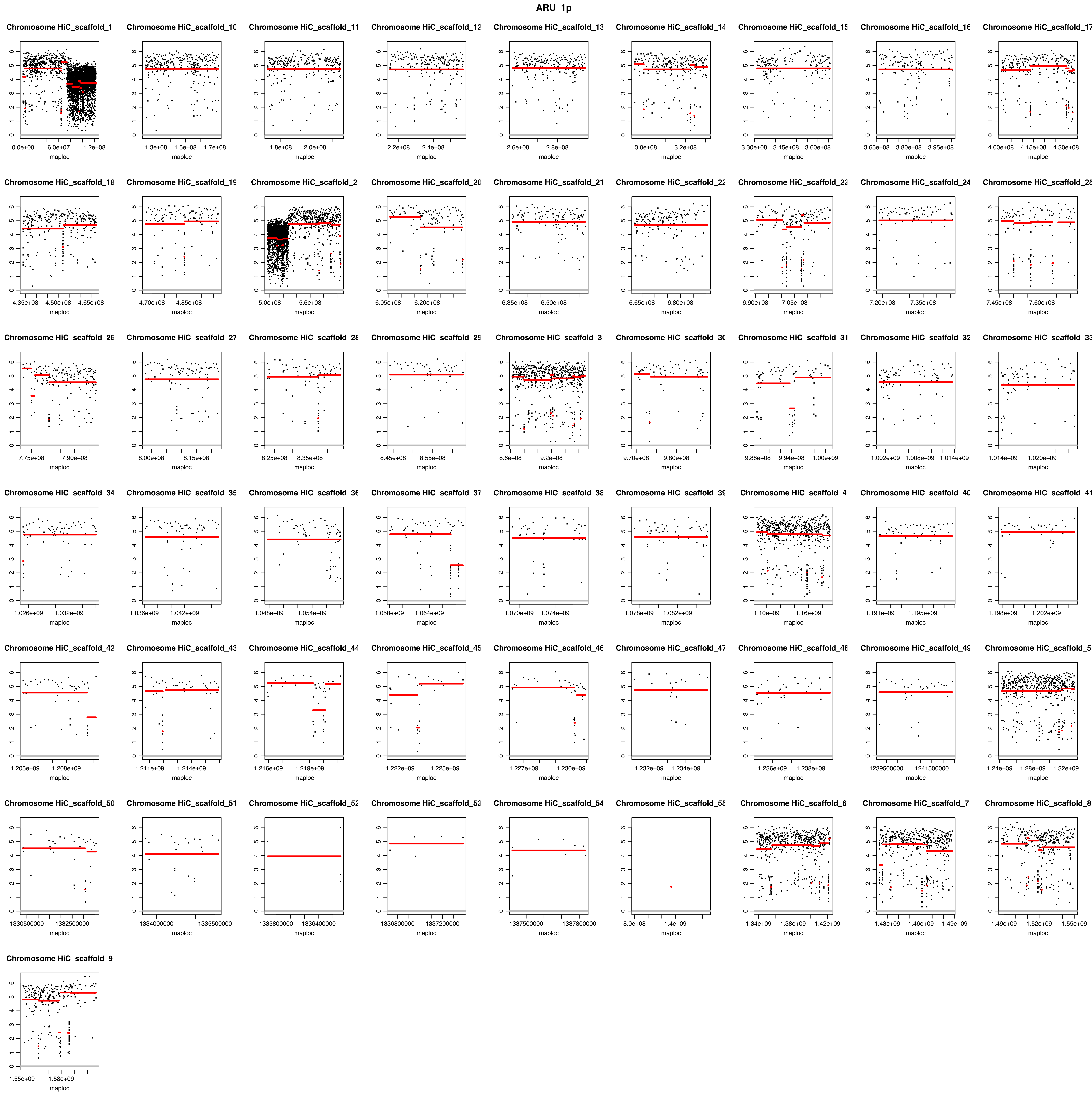

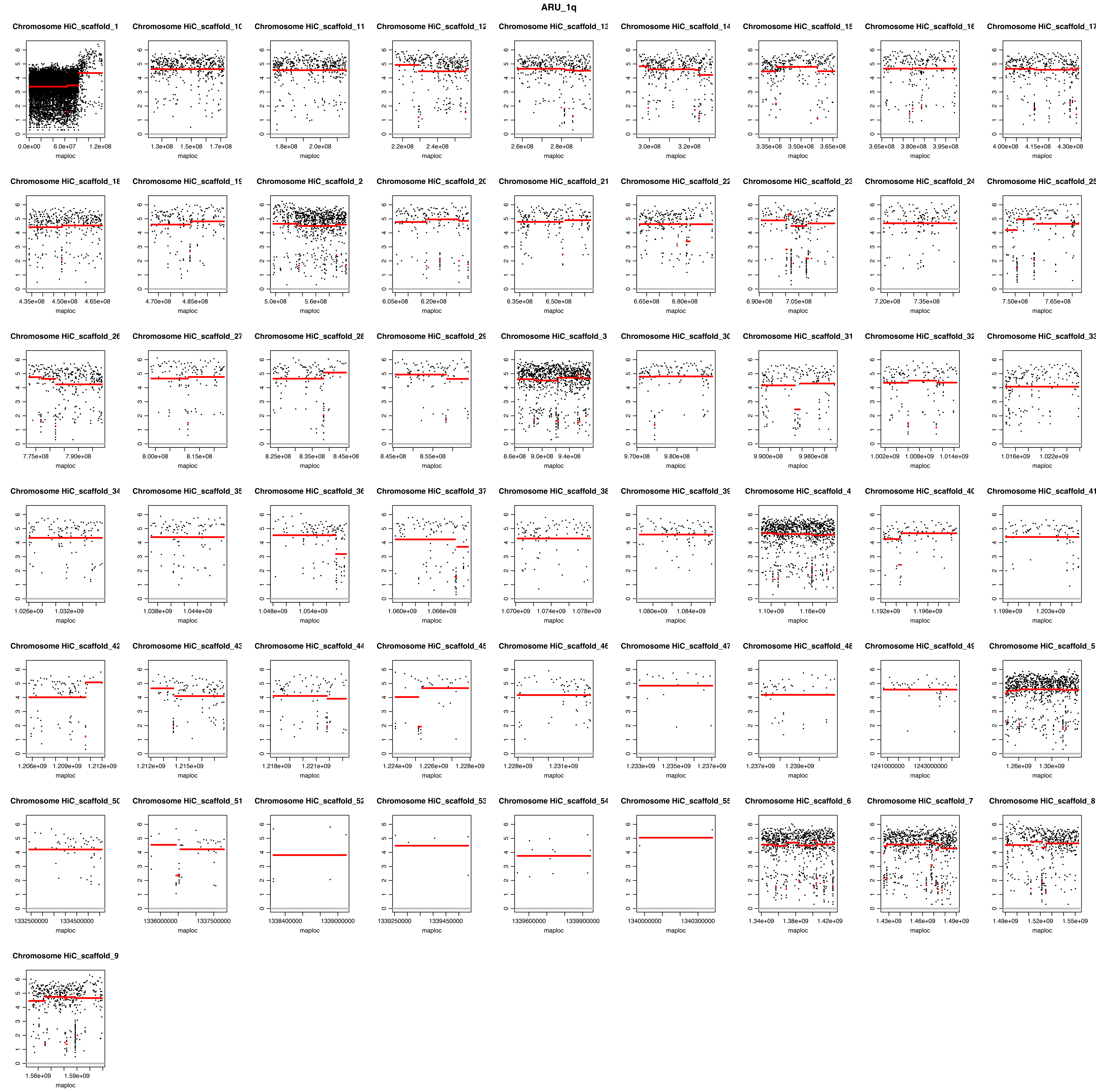

ARU_1q
Chromosome HiC_scaffold_1
Chromosome HiC_scaffold_10
Chromosome HiC_scaffold_11
Chromosome HiC_scaffold_12
Chromosome HiC_scaffold_13
Chromosome HiC_scaffold_14
Chromosome HiC_scaffold_15
Chromosome HiC_scaffold_16
Chromosome HiC_scaffold_17
Chromosome HiC_scaffold_18
Chromosome HiC_scaffold_19
Chromosome HiC_scaffold_2
Chromosome HiC_scaffold_20
Chromosome HiC_scaffold_21
Chromosome HiC_scaffold_22
Chromosome HiC_scaffold_23
Chromosome HiC_scaffold_24
Chromosome HiC_scaffold_25
Chromosome HiC_scaffold_26
Chromosome HiC_scaffold_27
Chromosome HiC_scaffold_28
Chromosome HiC_scaffold_29
Chromosome HiC_scaffold_3
Chromosome HiC_scaffold_30
Chromosome HiC_scaffold_31
Chromosome HiC_scaffold_32
Chromosome HiC_scaffold_33
Chromosome HiC_scaffold_34
Chromosome HiC_scaffold_35
Chromosome HiC_scaffold_36
Chromosome HiC_scaffold_37
Chromosome HiC_scaffold_38
Chromosome HiC_scaffold_39
Chromosome HiC_scaffold_4
Chromosome HiC_scaffold_40
Chromosome HiC_scaffold_41
Chromosome HiC_scaffold_42
Chromosome HiC_scaffold_43
Chromosome HiC_scaffold_44
Chromosome HiC_scaffold_45
Chromosome HiC_scaffold_46
Chromosome HiC_scaffold_47
Chromosome HiC_scaffold_48
Chromosome HiC_scaffold_49
Chromosome HiC_scaffold_5
Chromosome HiC_scaffold_50
Chromosome HiC_scaffold_51
Chromosome HiC_scaffold_52
Chromosome HiC_scaffold_53
Chromosome HiC_scaffold_54
Chromosome HiC_scaffold_55
Chromosome HiC_scaffold_6
Chromosome HiC_scaffold_7
Chromosome HiC_scaffold_8
Chromosome HiC_scaffold_9
maploc

ARU_2q

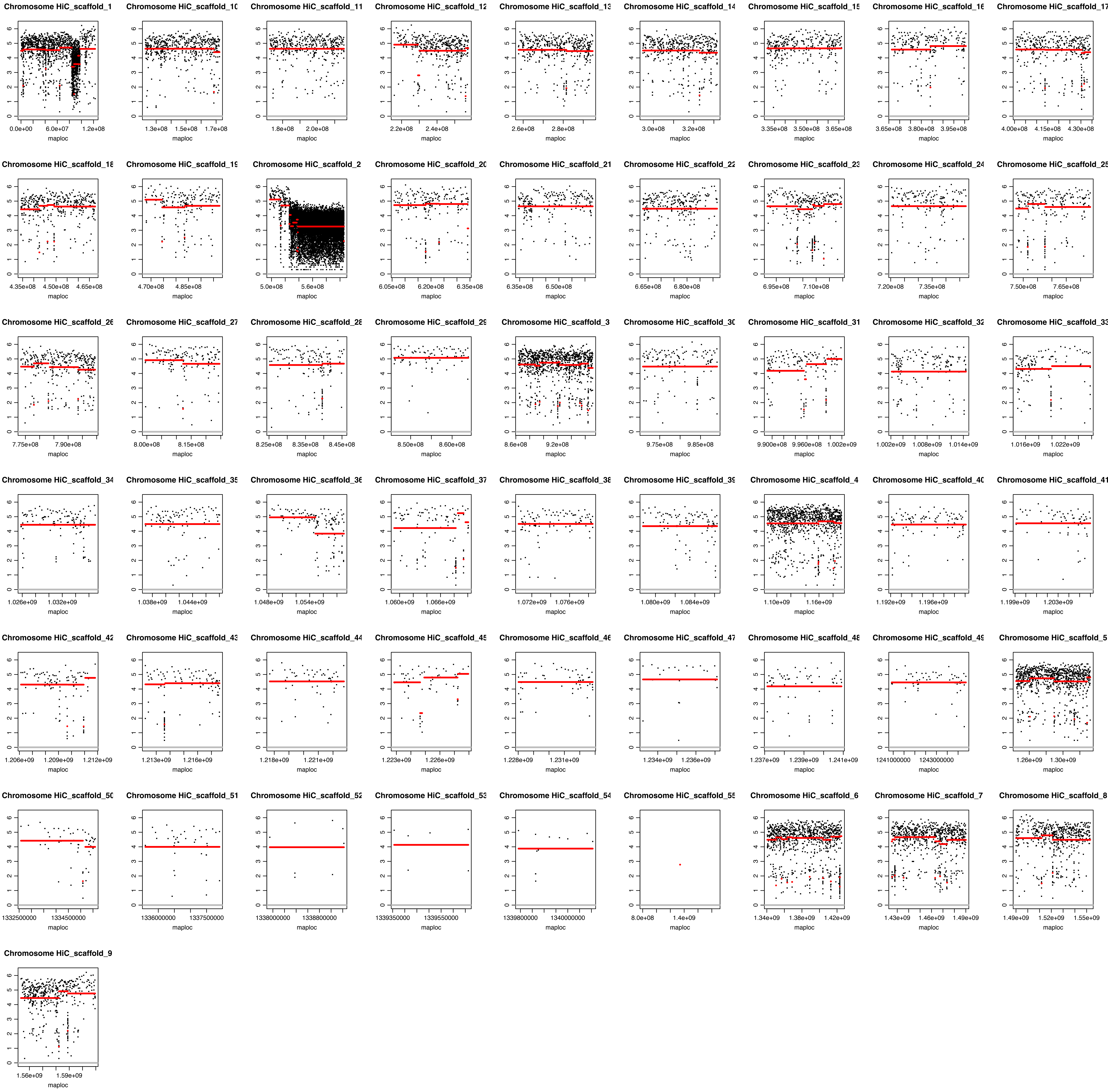

Chromosome HiC_scaffold_1
0.0e+00 6.0e+07 1.2e+08
maploc
Chromosome HiC_scaffold_10
1.3e+08 1.5e+08 1.7e+08
maploc
Chromosome HiC_scaffold_11
1.8e+08 2.0e+08
maploc
Chromosome HiC_scaffold_12
2.2e+08 2.4e+08
maploc
Chromosome HiC_scaffold_13
2.6e+08 2.8e+08
maploc
Chromosome HiC_scaffold_14
3.0e+08 3.2e+08
maploc
Chromosome HiC_scaffold_15
3.35e+08 3.50e+08 3.65e+08
maploc
Chromosome HiC_scaffold_16
3.65e+08 3.80e+08 3.95e+08
maploc
Chromosome HiC_scaffold_17
4.00e+08 4.15e+08 4.30e+08
maploc
Chromosome HiC_scaffold_18
4.35e+08 4.50e+08 4.65e+08
maploc
Chromosome HiC_scaffold_19
4.70e+08 4.85e+08
maploc
Chromosome HiC_scaffold_2
5.0e+08 5.6e+08
maploc
Chromosome HiC_scaffold_20
6.05e+08 6.20e+08 6.35e+08
maploc
Chromosome HiC_scaffold_21
6.35e+08 6.50e+08
maploc
Chromosome HiC_scaffold_22
6.65e+08 6.80e+08
maploc
Chromosome HiC_scaffold_23
6.95e+08 7.10e+08
maploc
Chromosome HiC_scaffold_24
7.20e+08 7.35e+08
maploc
Chromosome HiC_scaffold_25
7.50e+08 7.65e+08
maploc
Chromosome HiC_scaffold_26
7.75e+08 7.90e+08
maploc
Chromosome HiC_scaffold_27
8.00e+08 8.15e+08
maploc
Chromosome HiC_scaffold_28
8.25e+08 8.35e+08 8.45e+08
maploc
Chromosome HiC_scaffold_29
8.50e+08 8.60e+08
maploc
Chromosome HiC_scaffold_3
8.6e+08 9.2e+08
maploc
Chromosome HiC_scaffold_30
9.75e+08 9.85e+08
maploc
Chromosome HiC_scaffold_31
9.900e+08 9.960e+08 1.002e+09
maploc
Chromosome HiC_scaffold_32
1.002e+09 1.008e+09 1.014e+09
maploc
Chromosome HiC_scaffold_33
1.016e+09 1.022e+09
maploc
Chromosome HiC_scaffold_34
1.026e+09 1.032e+09
maploc
Chromosome HiC_scaffold_35
1.038e+09 1.044e+09
maploc
Chromosome HiC_scaffold_36
1.048e+09 1.054e+09
maploc
Chromosome HiC_scaffold_37
1.060e+09 1.066e+09
maploc
Chromosome HiC_scaffold_38
1.072e+09 1.076e+09
maploc
Chromosome HiC_scaffold_39
1.080e+09 1.084e+09
maploc
Chromosome HiC_scaffold_4
1.10e+09 1.16e+09
maploc
Chromosome HiC_scaffold_40
1.192e+09 1.196e+09
maploc
Chromosome HiC_scaffold_41
1.199e+09 1.203e+09
maploc
Chromosome HiC_scaffold_42
1.206e+09 1.209e+09 1.212e+09
maploc
Chromosome HiC_scaffold_43
1.213e+09 1.216e+09
maploc
Chromosome HiC_scaffold_44
1.218e+09 1.221e+09
maploc
Chromosome HiC_scaffold_45
1.223e+09 1.226e+09
maploc
Chromosome HiC_scaffold_46
1.228e+09 1.231e+09
maploc
Chromosome HiC_scaffold_47
1.234e+09 1.236e+09
maploc
Chromosome HiC_scaffold_48
1.237e+09 1.239e+09 1.241e+09
maploc
Chromosome HiC_scaffold_49
1241000000 1243000000
maploc
Chromosome HiC_scaffold_5
1.26e+09 1.30e+09
maploc
Chromosome HiC_scaffold_50
1332500000 1334500000
maploc
Chromosome HiC_scaffold_51
1336000000 1337500000
maploc
Chromosome HiC_scaffold_52
1338000000 1338800000
maploc
Chromosome HiC_scaffold_53
1339350000 1339550000
maploc
Chromosome HiC_scaffold_54
1339800000 1340000000
maploc
Chromosome HiC_scaffold_55
8.0e+08 1.4e+09
maploc
Chromosome HiC_scaffold_6
1.34e+09 1.38e+09 1.42e+09
maploc
Chromosome HiC_scaffold_7
1.43e+09 1.46e+09 1.49e+09
maploc
Chromosome HiC_scaffold_8
1.49e+09 1.52e+09 1.55e+09
maploc
Chromosome HiC_scaffold_9
1.56e+09 1.59e+09
maploc

ARU_3

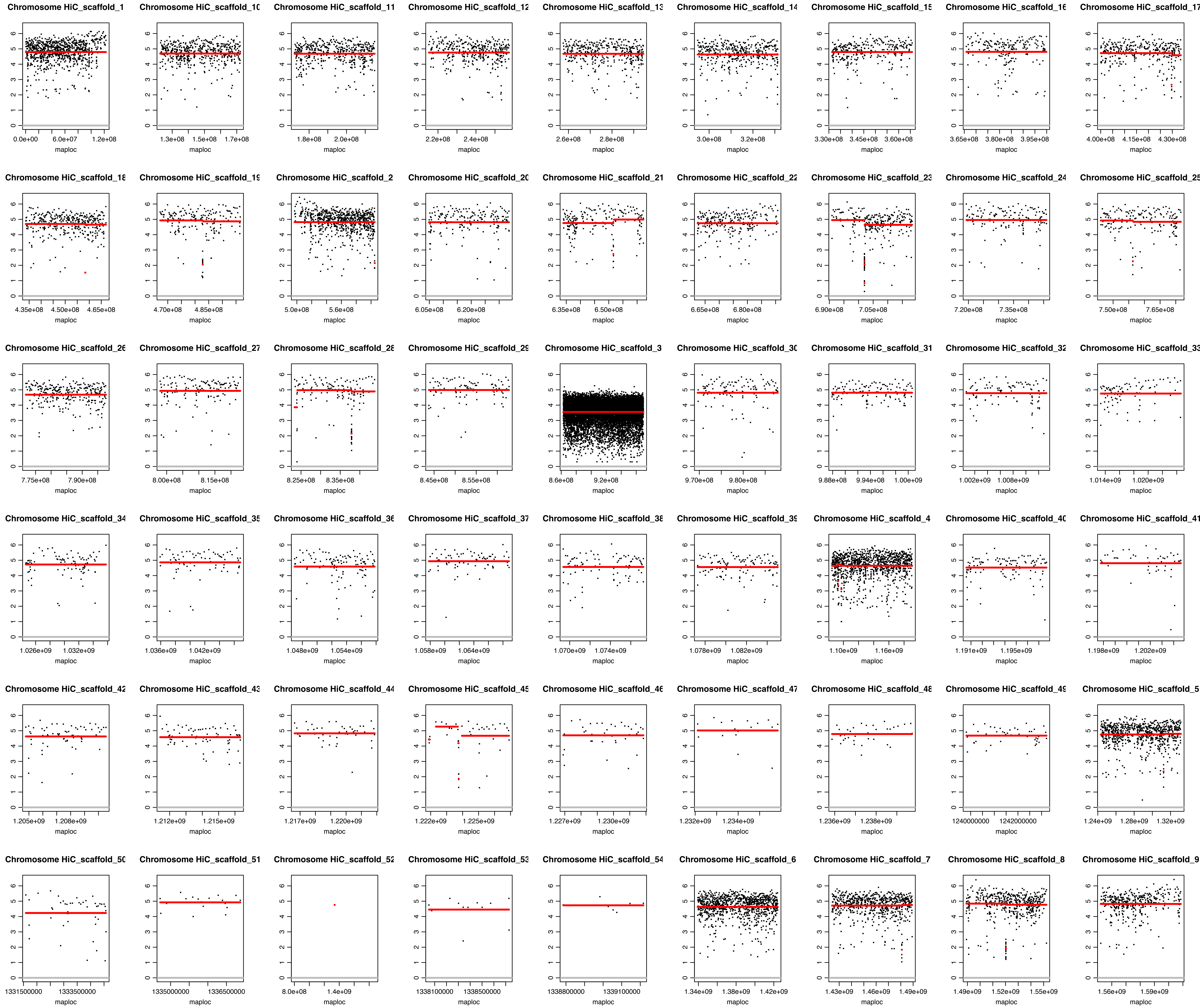

Chromosome HiC_scaffold_1
Chromosome HiC_scaffold_10
Chromosome HiC_scaffold_11
Chromosome HiC_scaffold_12
Chromosome HiC_scaffold_13
Chromosome HiC_scaffold_14
Chromosome HiC_scaffold_15
Chromosome HiC_scaffold_16
Chromosome HiC_scaffold_17
Chromosome HiC_scaffold_18
Chromosome HiC_scaffold_19
Chromosome HiC_scaffold_2
Chromosome HiC_scaffold_20
Chromosome HiC_scaffold_21
Chromosome HiC_scaffold_22
Chromosome HiC_scaffold_23
Chromosome HiC_scaffold_24
Chromosome HiC_scaffold_25
Chromosome HiC_scaffold_26
Chromosome HiC_scaffold_27
Chromosome HiC_scaffold_28
Chromosome HiC_scaffold_29
Chromosome HiC_scaffold_3
Chromosome HiC_scaffold_30
Chromosome HiC_scaffold_31
Chromosome HiC_scaffold_32
Chromosome HiC_scaffold_33
Chromosome HiC_scaffold_34
Chromosome HiC_scaffold_35
Chromosome HiC_scaffold_36
Chromosome HiC_scaffold_37
Chromosome HiC_scaffold_38
Chromosome HiC_scaffold_39
Chromosome HiC_scaffold_4
Chromosome HiC_scaffold_40
Chromosome HiC_scaffold_41
Chromosome HiC_scaffold_42
Chromosome HiC_scaffold_43
Chromosome HiC_scaffold_44
Chromosome HiC_scaffold_45
Chromosome HiC_scaffold_46
Chromosome HiC_scaffold_47
Chromosome HiC_scaffold_48
Chromosome HiC_scaffold_49
Chromosome HiC_scaffold_5
Chromosome HiC_scaffold_50
Chromosome HiC_scaffold_51
Chromosome HiC_scaffold_52
Chromosome HiC_scaffold_53
Chromosome HiC_scaffold_54
Chromosome HiC_scaffold_6
Chromosome HiC_scaffold_7
Chromosome HiC_scaffold_8
Chromosome HiC_scaffold_9
maploc

ARU_4

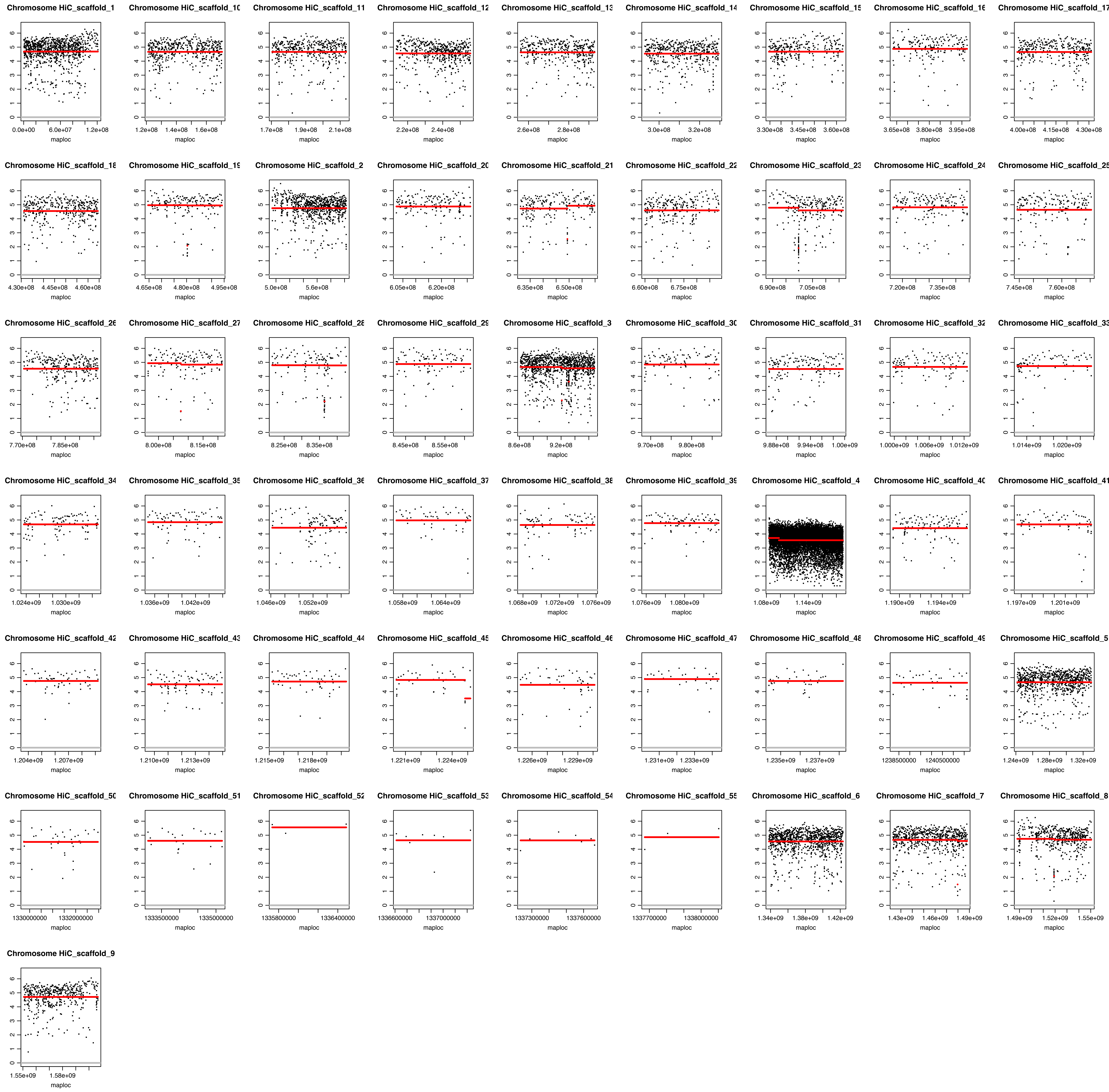
Chromosome HiC_scaffold_1
Chromosome HiC_scaffold_10
Chromosome HiC_scaffold_11
Chromosome HiC_scaffold_12
Chromosome HiC_scaffold_13
Chromosome HiC_scaffold_14
Chromosome HiC_scaffold_15
Chromosome HiC_scaffold_16
Chromosome HiC_scaffold_17
Chromosome HiC_scaffold_18
Chromosome HiC_scaffold_19
Chromosome HiC_scaffold_2
Chromosome HiC_scaffold_20
Chromosome HiC_scaffold_21
Chromosome HiC_scaffold_22
Chromosome HiC_scaffold_23
Chromosome HiC_scaffold_24
Chromosome HiC_scaffold_25
Chromosome HiC_scaffold_26
Chromosome HiC_scaffold_27
Chromosome HiC_scaffold_28
Chromosome HiC_scaffold_29
Chromosome HiC_scaffold_3
Chromosome HiC_scaffold_30
Chromosome HiC_scaffold_31
Chromosome HiC_scaffold_32
Chromosome HiC_scaffold_33
Chromosome HiC_scaffold_34
Chromosome HiC_scaffold_35
Chromosome HiC_scaffold_36
Chromosome HiC_scaffold_37
Chromosome HiC_scaffold_38
Chromosome HiC_scaffold_39
Chromosome HiC_scaffold_4
Chromosome HiC_scaffold_40
Chromosome HiC_scaffold_41
Chromosome HiC_scaffold_42
Chromosome HiC_scaffold_43
Chromosome HiC_scaffold_44
Chromosome HiC_scaffold_45
Chromosome HiC_scaffold_46
Chromosome HiC_scaffold_47
Chromosome HiC_scaffold_48
Chromosome HiC_scaffold_49
Chromosome HiC_scaffold_5
Chromosome HiC_scaffold_50
Chromosome HiC_scaffold_51
Chromosome HiC_scaffold_52
Chromosome HiC_scaffold_53
Chromosome HiC_scaffold_54
Chromosome HiC_scaffold_55
Chromosome HiC_scaffold_6
Chromosome HiC_scaffold_7
Chromosome HiC_scaffold_8
Chromosome HiC_scaffold_9
maploc

ARU_5

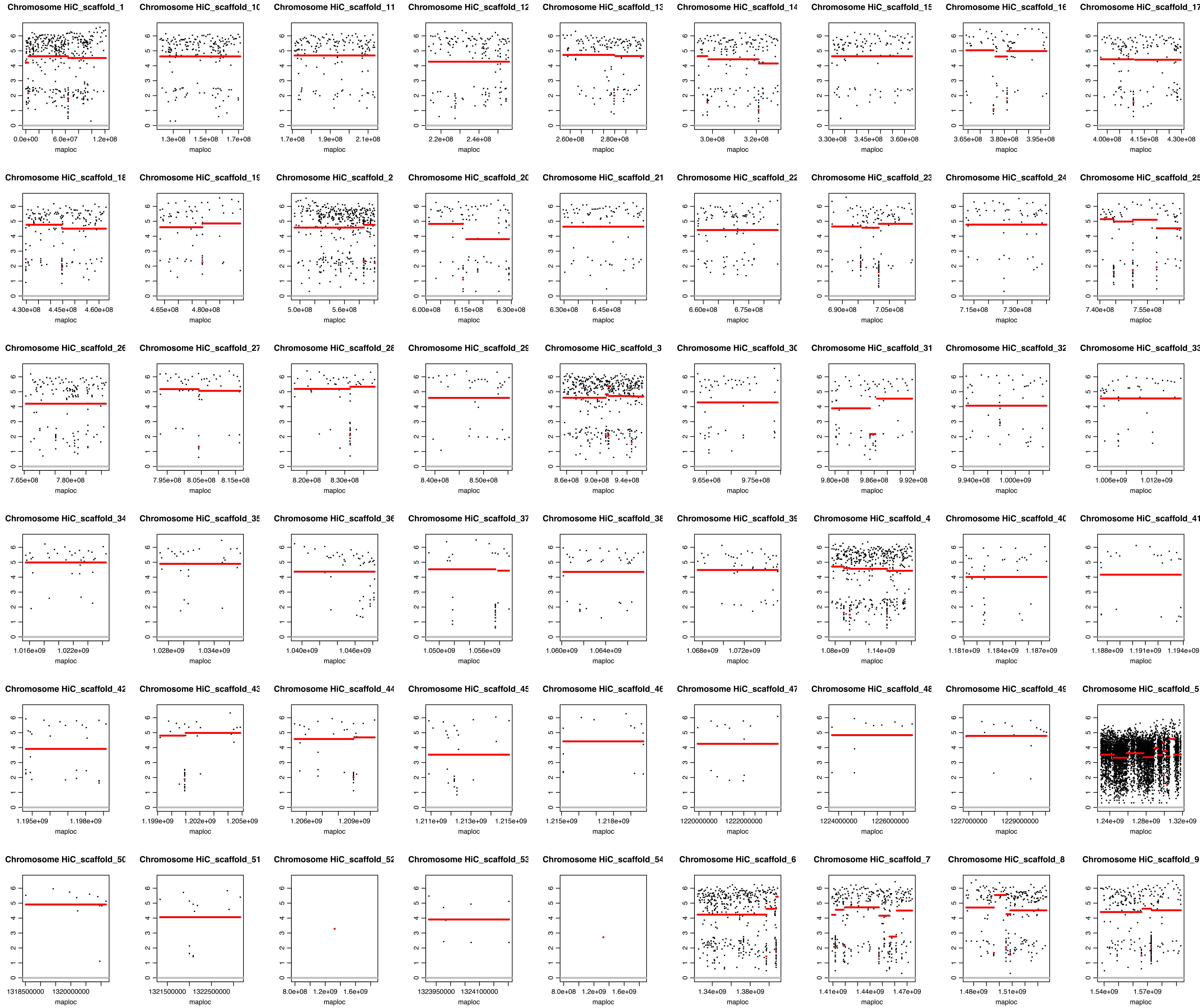

Chromosome HiC_scaffold_1
Chromosome HiC_scaffold_10
Chromosome HiC_scaffold_11
Chromosome HiC_scaffold_12
Chromosome HiC_scaffold_13
Chromosome HiC_scaffold_14
Chromosome HiC_scaffold_15
Chromosome HiC_scaffold_16
Chromosome HiC_scaffold_17
Chromosome HiC_scaffold_18
Chromosome HiC_scaffold_19
Chromosome HiC_scaffold_2
Chromosome HiC_scaffold_20
Chromosome HiC_scaffold_21
Chromosome HiC_scaffold_22
Chromosome HiC_scaffold_23
Chromosome HiC_scaffold_24
Chromosome HiC_scaffold_25
Chromosome HiC_scaffold_26
Chromosome HiC_scaffold_27
Chromosome HiC_scaffold_28
Chromosome HiC_scaffold_29
Chromosome HiC_scaffold_3
Chromosome HiC_scaffold_30
Chromosome HiC_scaffold_31
Chromosome HiC_scaffold_32
Chromosome HiC_scaffold_33
Chromosome HiC_scaffold_34
Chromosome HiC_scaffold_35
Chromosome HiC_scaffold_36
Chromosome HiC_scaffold_37
Chromosome HiC_scaffold_38
Chromosome HiC_scaffold_39
Chromosome HiC_scaffold_4
Chromosome HiC_scaffold_40
Chromosome HiC_scaffold_41
Chromosome HiC_scaffold_42
Chromosome HiC_scaffold_43
Chromosome HiC_scaffold_44
Chromosome HiC_scaffold_45
Chromosome HiC_scaffold_46
Chromosome HiC_scaffold_47
Chromosome HiC_scaffold_48
Chromosome HiC_scaffold_49
Chromosome HiC_scaffold_5
Chromosome HiC_scaffold_50
Chromosome HiC_scaffold_51
Chromosome HiC_scaffold_52
Chromosome HiC_scaffold_53
Chromosome HiC_scaffold_54
Chromosome HiC_scaffold_6
Chromosome HiC_scaffold_7
Chromosome HiC_scaffold_8
Chromosome HiC_scaffold_9
maploc

ARU_6

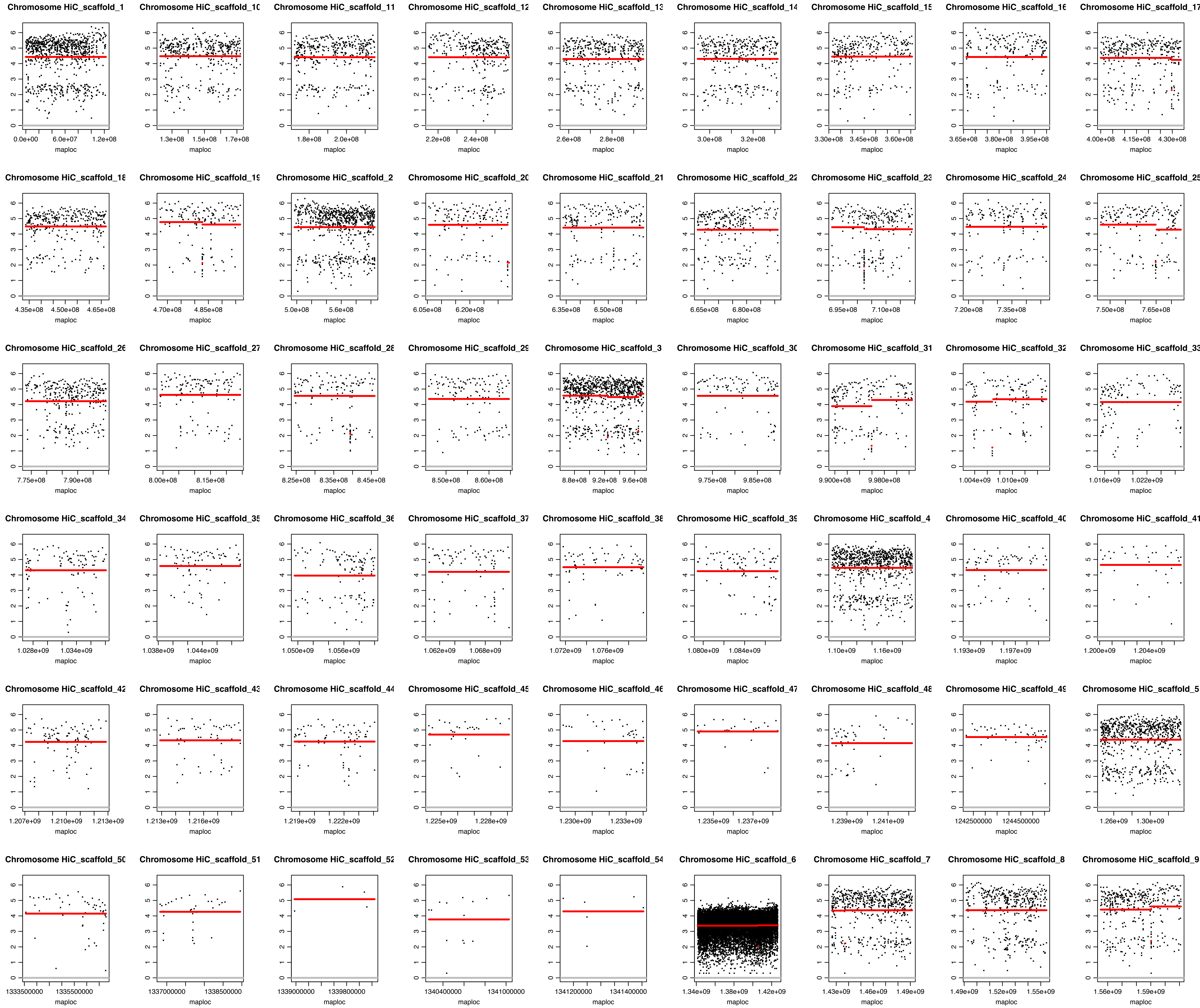
Chromosome HiC_scaffold_1
Chromosome HiC_scaffold_10
Chromosome HiC_scaffold_11
Chromosome HiC_scaffold_12
Chromosome HiC_scaffold_13
Chromosome HiC_scaffold_14
Chromosome HiC_scaffold_15
Chromosome HiC_scaffold_16
Chromosome HiC_scaffold_17
Chromosome HiC_scaffold_18
Chromosome HiC_scaffold_19
Chromosome HiC_scaffold_2
Chromosome HiC_scaffold_20
Chromosome HiC_scaffold_21
Chromosome HiC_scaffold_22
Chromosome HiC_scaffold_23
Chromosome HiC_scaffold_24
Chromosome HiC_scaffold_25
Chromosome HiC_scaffold_26
Chromosome HiC_scaffold_27
Chromosome HiC_scaffold_28
Chromosome HiC_scaffold_29
Chromosome HiC_scaffold_3
Chromosome HiC_scaffold_30
Chromosome HiC_scaffold_31
Chromosome HiC_scaffold_32
Chromosome HiC_scaffold_33
Chromosome HiC_scaffold_34
Chromosome HiC_scaffold_35
Chromosome HiC_scaffold_36
Chromosome HiC_scaffold_37
Chromosome HiC_scaffold_38
Chromosome HiC_scaffold_39
Chromosome HiC_scaffold_4
Chromosome HiC_scaffold_40
Chromosome HiC_scaffold_41
Chromosome HiC_scaffold_42
Chromosome HiC_scaffold_43
Chromosome HiC_scaffold_44
Chromosome HiC_scaffold_45
Chromosome HiC_scaffold_46
Chromosome HiC_scaffold_47
Chromosome HiC_scaffold_48
Chromosome HiC_scaffold_49
Chromosome HiC_scaffold_5
Chromosome HiC_scaffold_50
Chromosome HiC_scaffold_51
Chromosome HiC_scaffold_52
Chromosome HiC_scaffold_53
Chromosome HiC_scaffold_54
Chromosome HiC_scaffold_6
Chromosome HiC_scaffold_7
Chromosome HiC_scaffold_8
Chromosome HiC_scaffold_9
maploc

ARU_7.reg

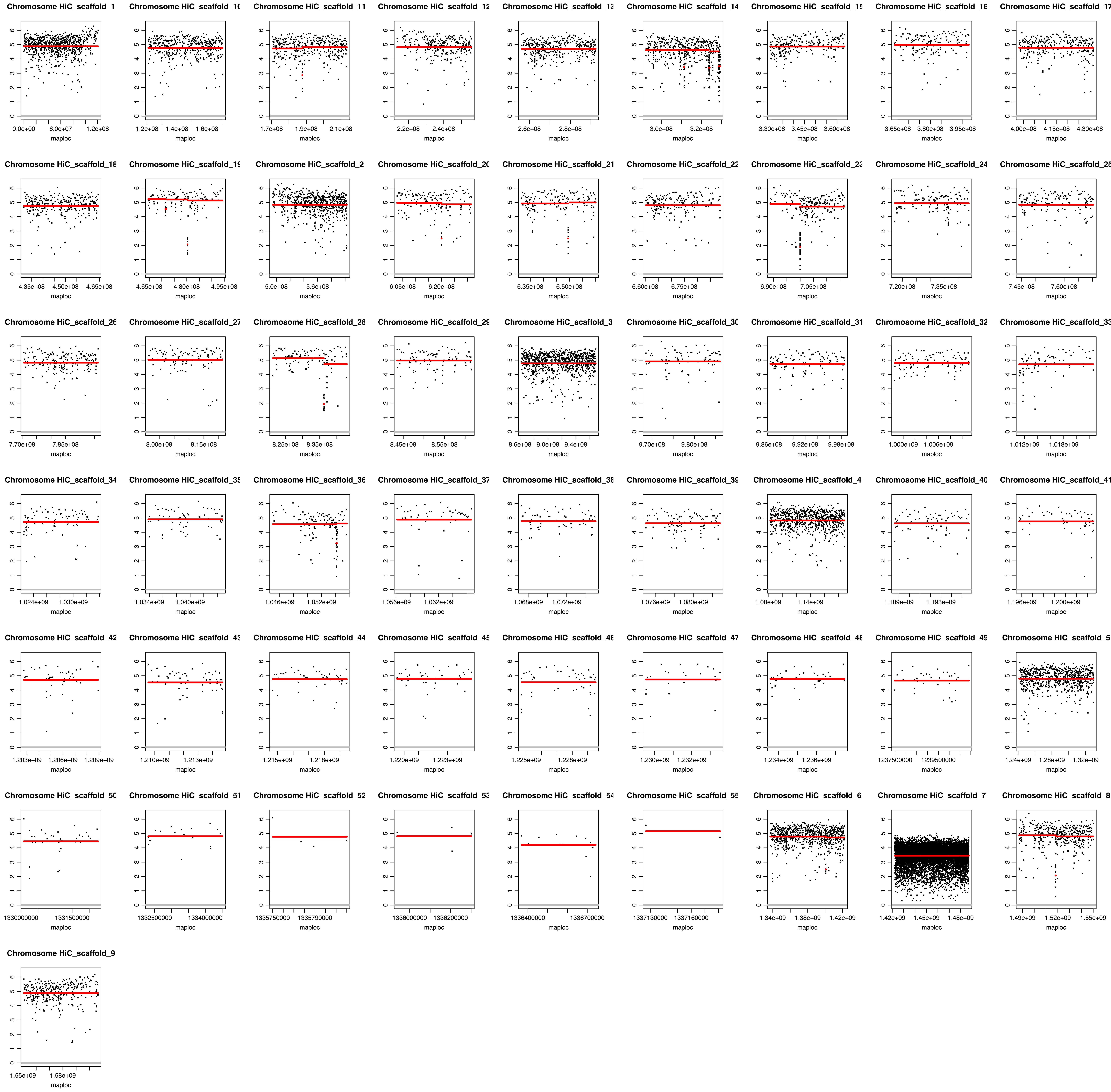
Chromosome HiC_scaffold_1
Chromosome HiC_scaffold_10
Chromosome HiC_scaffold_11
Chromosome HiC_scaffold_12
Chromosome HiC_scaffold_13
Chromosome HiC_scaffold_14
Chromosome HiC_scaffold_15
Chromosome HiC_scaffold_16
Chromosome HiC_scaffold_17
Chromosome HiC_scaffold_18
Chromosome HiC_scaffold_19
Chromosome HiC_scaffold_2
Chromosome HiC_scaffold_20
Chromosome HiC_scaffold_21
Chromosome HiC_scaffold_22
Chromosome HiC_scaffold_23
Chromosome HiC_scaffold_24
Chromosome HiC_scaffold_25
Chromosome HiC_scaffold_26
Chromosome HiC_scaffold_27
Chromosome HiC_scaffold_28
Chromosome HiC_scaffold_29
Chromosome HiC_scaffold_3
Chromosome HiC_scaffold_30
Chromosome HiC_scaffold_31
Chromosome HiC_scaffold_32
Chromosome HiC_scaffold_33
Chromosome HiC_scaffold_34
Chromosome HiC_scaffold_35
Chromosome HiC_scaffold_36
Chromosome HiC_scaffold_37
Chromosome HiC_scaffold_38
Chromosome HiC_scaffold_39
Chromosome HiC_scaffold_4
Chromosome HiC_scaffold_40
Chromosome HiC_scaffold_41
Chromosome HiC_scaffold_42
Chromosome HiC_scaffold_43
Chromosome HiC_scaffold_44
Chromosome HiC_scaffold_45
Chromosome HiC_scaffold_46
Chromosome HiC_scaffold_47
Chromosome HiC_scaffold_48
Chromosome HiC_scaffold_49
Chromosome HiC_scaffold_5
Chromosome HiC_scaffold_50
Chromosome HiC_scaffold_51
Chromosome HiC_scaffold_52
Chromosome HiC_scaffold_53
Chromosome HiC_scaffold_54
Chromosome HiC_scaffold_55
Chromosome HiC_scaffold_6
Chromosome HiC_scaffold_7
Chromosome HiC_scaffold_8
Chromosome HiC_scaffold_9
maploc

ARU_8q.reg

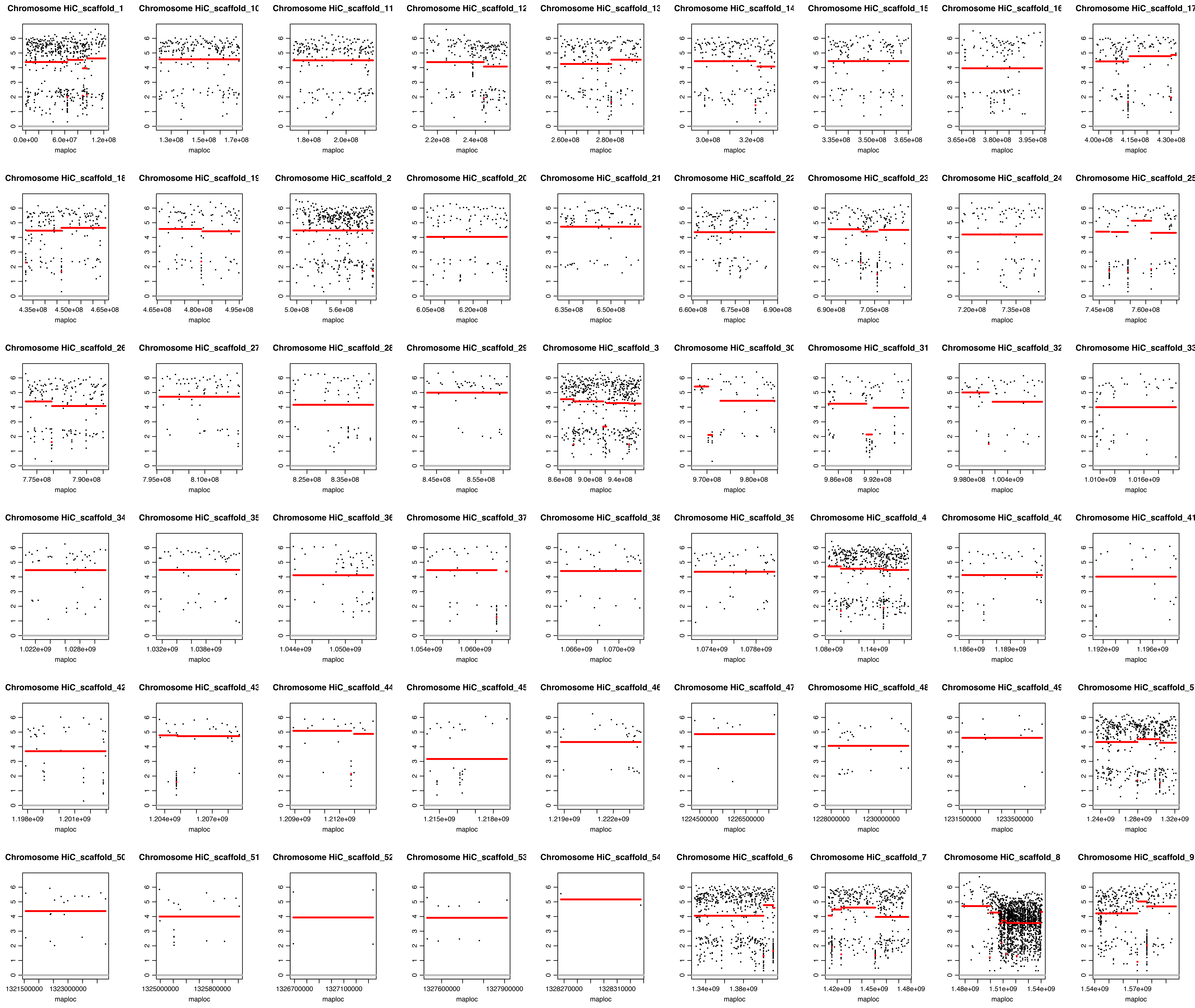
Chromosome HiC_scaffold_1
Chromosome HiC_scaffold_10
Chromosome HiC_scaffold_11
Chromosome HiC_scaffold_12
Chromosome HiC_scaffold_13
Chromosome HiC_scaffold_14
Chromosome HiC_scaffold_15
Chromosome HiC_scaffold_16
Chromosome HiC_scaffold_17
Chromosome HiC_scaffold_18
Chromosome HiC_scaffold_19
Chromosome HiC_scaffold_2
Chromosome HiC_scaffold_20
Chromosome HiC_scaffold_21
Chromosome HiC_scaffold_22
Chromosome HiC_scaffold_23
Chromosome HiC_scaffold_24
Chromosome HiC_scaffold_25
Chromosome HiC_scaffold_26
Chromosome HiC_scaffold_27
Chromosome HiC_scaffold_28
Chromosome HiC_scaffold_29
Chromosome HiC_scaffold_3
Chromosome HiC_scaffold_30
Chromosome HiC_scaffold_31
Chromosome HiC_scaffold_32
Chromosome HiC_scaffold_33
Chromosome HiC_scaffold_34
Chromosome HiC_scaffold_35
Chromosome HiC_scaffold_36
Chromosome HiC_scaffold_37
Chromosome HiC_scaffold_38
Chromosome HiC_scaffold_39
Chromosome HiC_scaffold_4
Chromosome HiC_scaffold_40
Chromosome HiC_scaffold_41
Chromosome HiC_scaffold_42
Chromosome HiC_scaffold_43
Chromosome HiC_scaffold_44
Chromosome HiC_scaffold_45
Chromosome HiC_scaffold_46
Chromosome HiC_scaffold_47
Chromosome HiC_scaffold_48
Chromosome HiC_scaffold_49
Chromosome HiC_scaffold_5
Chromosome HiC_scaffold_50
Chromosome HiC_scaffold_51
Chromosome HiC_scaffold_52
Chromosome HiC_scaffold_53
Chromosome HiC_scaffold_54
Chromosome HiC_scaffold_6
Chromosome HiC_scaffold_7
Chromosome HiC_scaffold_8
Chromosome HiC_scaffold_9
maploc

ARU_9

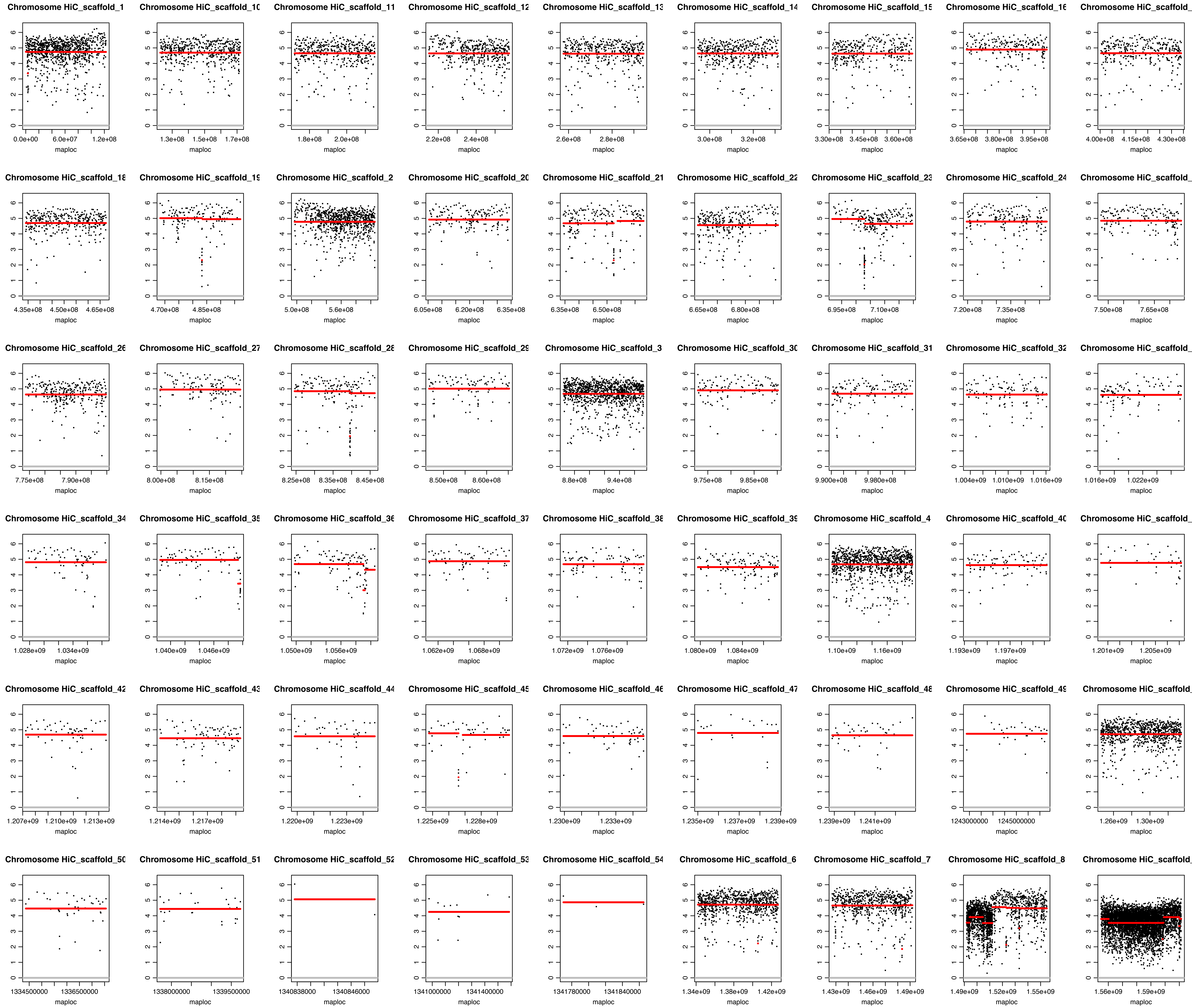
Chromosome HiC_scaffold_1
Chromosome HiC_scaffold_10
Chromosome HiC_scaffold_11
Chromosome HiC_scaffold_12
Chromosome HiC_scaffold_13
Chromosome HiC_scaffold_14
Chromosome HiC_scaffold_15
Chromosome HiC_scaffold_16
Chromosome HiC_scaffold_17
Chromosome HiC_scaffold_18
Chromosome HiC_scaffold_19
Chromosome HiC_scaffold_2
Chromosome HiC_scaffold_20
Chromosome HiC_scaffold_21
Chromosome HiC_scaffold_22
Chromosome HiC_scaffold_23
Chromosome HiC_scaffold_24
Chromosome HiC_scaffold_25
Chromosome HiC_scaffold_26
Chromosome HiC_scaffold_27
Chromosome HiC_scaffold_28
Chromosome HiC_scaffold_29
Chromosome HiC_scaffold_3
Chromosome HiC_scaffold_30
Chromosome HiC_scaffold_31
Chromosome HiC_scaffold_32
Chromosome HiC_scaffold_33
Chromosome HiC_scaffold_34
Chromosome HiC_scaffold_35
Chromosome HiC_scaffold_36
Chromosome HiC_scaffold_37
Chromosome HiC_scaffold_38
Chromosome HiC_scaffold_39
Chromosome HiC_scaffold_4
Chromosome HiC_scaffold_40
Chromosome HiC_scaffold_41
Chromosome HiC_scaffold_42
Chromosome HiC_scaffold_43
Chromosome HiC_scaffold_44
Chromosome HiC_scaffold_45
Chromosome HiC_scaffold_46
Chromosome HiC_scaffold_47
Chromosome HiC_scaffold_48
Chromosome HiC_scaffold_49
Chromosome HiC_scaffold_5
Chromosome HiC_scaffold_50
Chromosome HiC_scaffold_51
Chromosome HiC_scaffold_52
Chromosome HiC_scaffold_53
Chromosome HiC_scaffold_54
Chromosome HiC_scaffold_6
Chromosome HiC_scaffold_7
Chromosome HiC_scaffold_8
Chromosome HiC_scaffold_9
maploc

ARU_13

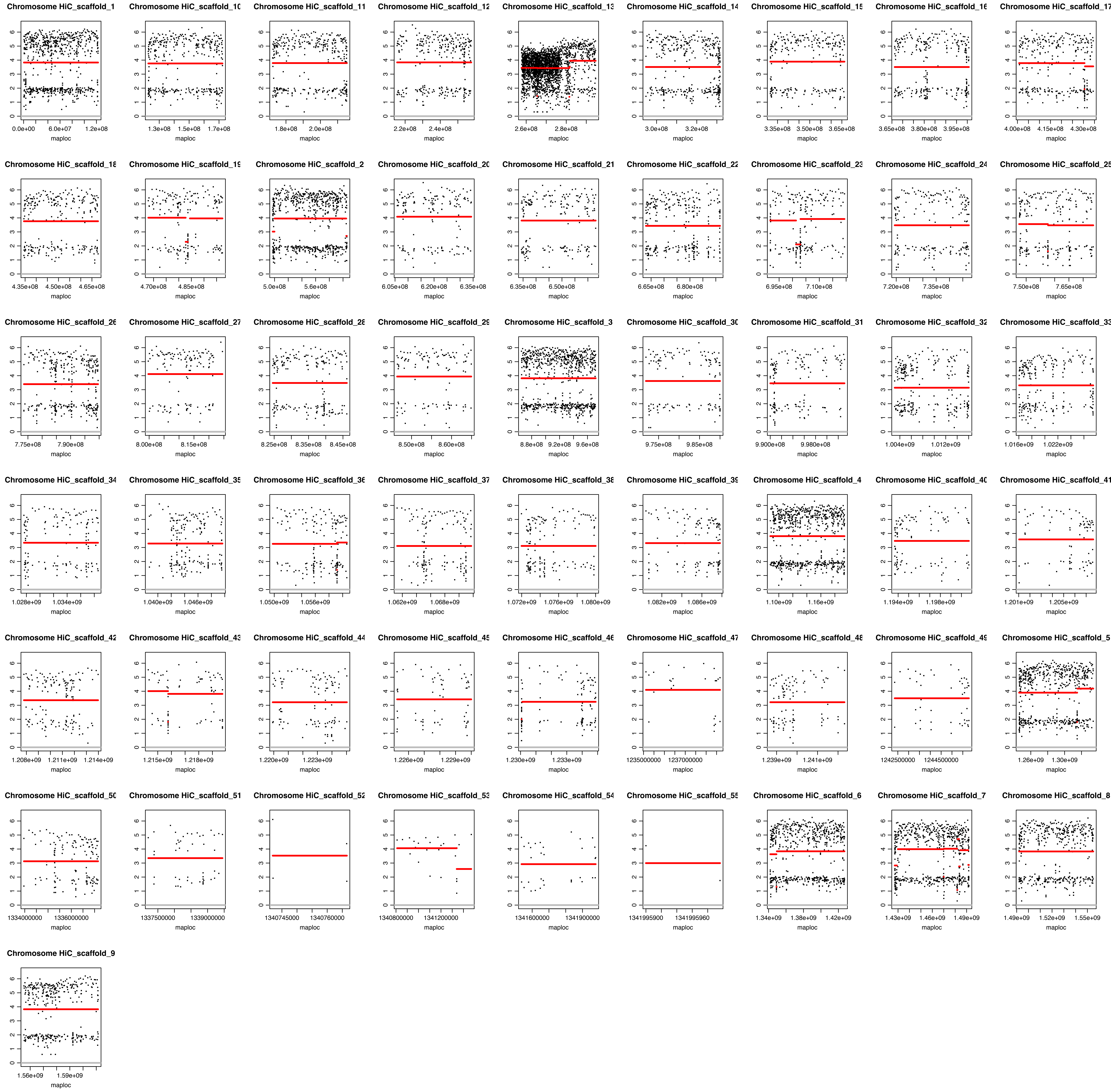
Chromosome HiC_scaffold_1
0.0e+00 6.0e+07 1.2e+08
Chromosome HiC_scaffold_10
1.3e+08 1.5e+08 1.7e+08
Chromosome HiC_scaffold_11
1.8e+08 2.0e+08
Chromosome HiC_scaffold_12
2.2e+08 2.4e+08
Chromosome HiC_scaffold_13
2.6e+08 2.8e+08
Chromosome HiC_scaffold_14
3.0e+08 3.2e+08
Chromosome HiC_scaffold_15
3.35e+08 3.50e+08 3.65e+08
Chromosome HiC_scaffold_16
3.65e+08 3.80e+08 3.95e+08
Chromosome HiC_scaffold_17
4.00e+08 4.15e+08 4.30e+08
Chromosome HiC_scaffold_18
4.35e+08 4.50e+08 4.65e+08
Chromosome HiC_scaffold_19
4.70e+08 4.85e+08
Chromosome HiC_scaffold_2
5.0e+08 5.6e+08
Chromosome HiC_scaffold_20
6.05e+08 6.20e+08 6.35e+08
Chromosome HiC_scaffold_21
6.35e+08 6.50e+08
Chromosome HiC_scaffold_22
6.65e+08 6.80e+08
Chromosome HiC_scaffold_23
6.95e+08 7.10e+08
Chromosome HiC_scaffold_24
7.20e+08 7.35e+08
Chromosome HiC_scaffold_25
7.50e+08 7.65e+08
Chromosome HiC_scaffold_26
7.75e+08 7.90e+08
Chromosome HiC_scaffold_27
8.00e+08 8.15e+08
Chromosome HiC_scaffold_28
8.25e+08 8.35e+08 8.45e+08
Chromosome HiC_scaffold_29
8.50e+08 8.60e+08
Chromosome HiC_scaffold_3
8.8e+08 9.2e+08 9.6e+08
Chromosome HiC_scaffold_30
9.75e+08 9.85e+08
Chromosome HiC_scaffold_31
9.900e+08 9.980e+08
Chromosome HiC_scaffold_32
1.004e+09 1.012e+09
Chromosome HiC_scaffold_33
1.016e+09 1.022e+09
Chromosome HiC_scaffold_34
1.028e+09 1.034e+09
Chromosome HiC_scaffold_35
1.040e+09 1.046e+09
Chromosome HiC_scaffold_36
1.050e+09 1.056e+09
Chromosome HiC_scaffold_37
1.062e+09 1.068e+09
Chromosome HiC_scaffold_38
1.072e+09 1.076e+09 1.080e+09
Chromosome HiC_scaffold_39
1.082e+09 1.086e+09
Chromosome HiC_scaffold_4
1.10e+09 1.16e+09
Chromosome HiC_scaffold_40
1.194e+09 1.198e+09
Chromosome HiC_scaffold_41
1.201e+09 1.205e+09
Chromosome HiC_scaffold_42
1.208e+09 1.211e+09 1.214e+09
Chromosome HiC_scaffold_43
1.215e+09 1.218e+09
Chromosome HiC_scaffold_44
1.220e+09 1.223e+09
Chromosome HiC_scaffold_45
1.226e+09 1.229e+09
Chromosome HiC_scaffold_46
1.230e+09 1.233e+09
Chromosome HiC_scaffold_47
1235000000 1237000000
Chromosome HiC_scaffold_48
1.239e+09 1.241e+09
Chromosome HiC_scaffold_49
1242500000 1244500000
Chromosome HiC_scaffold_5
1.26e+09 1.30e+09
Chromosome HiC_scaffold_50
1334000000 1336000000
Chromosome HiC_scaffold_51
1337500000 1339000000
Chromosome HiC_scaffold_52
1340745000 1340760000
Chromosome HiC_scaffold_53
1340800000 1341200000
Chromosome HiC_scaffold_54
1341600000 1341900000
Chromosome HiC_scaffold_55
1341995900 1341995960
Chromosome HiC_scaffold_6
1.34e+09 1.38e+09 1.42e+09
Chromosome HiC_scaffold_7
1.43e+09 1.46e+09 1.49e+09
Chromosome HiC_scaffold_8
1.49e+09 1.52e+09 1.55e+09
Chromosome HiC_scaffold_9
1.56e+09 1.59e+09
maploc

ARU_14

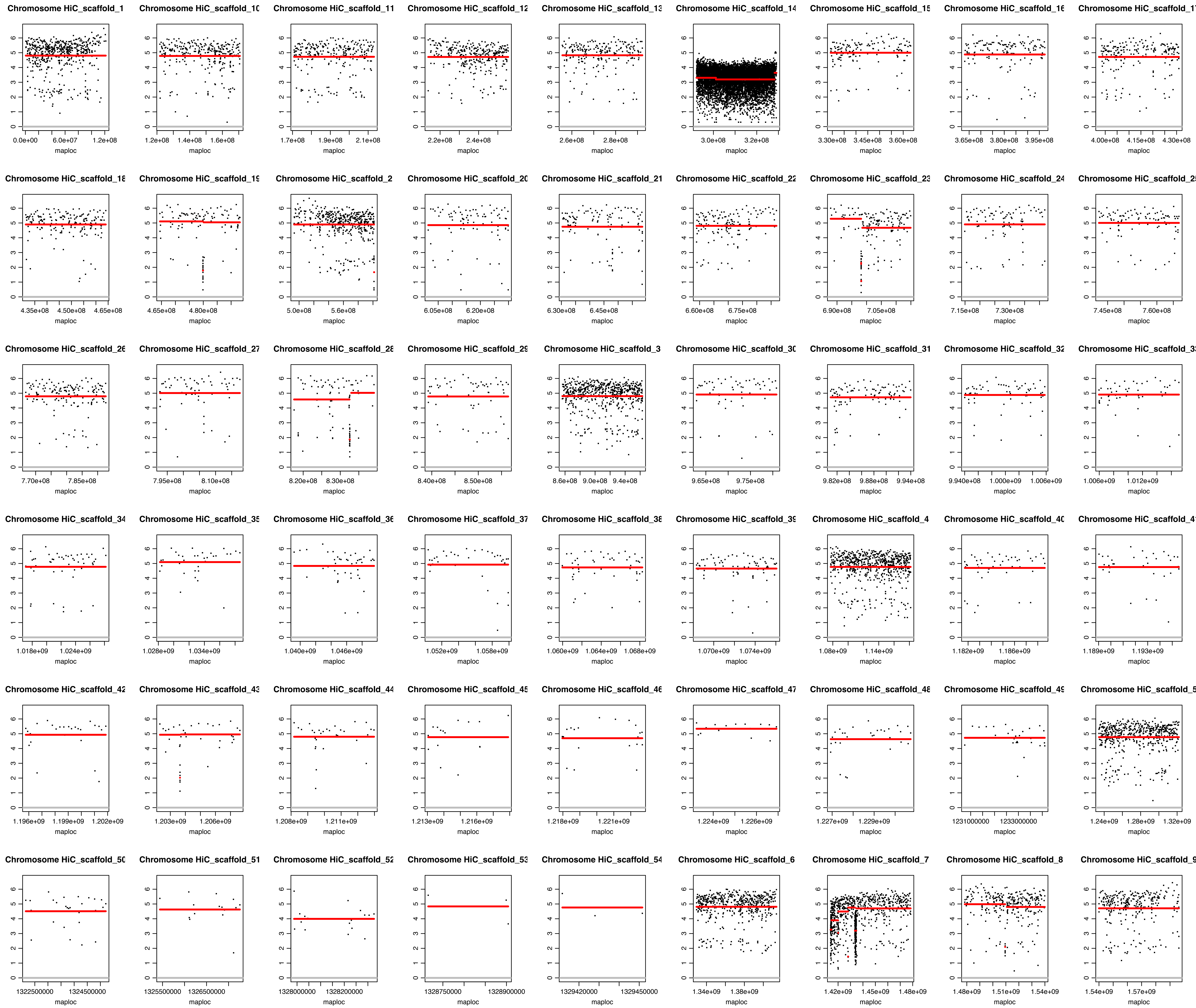

Chromosome HiC_scaffold_1
0.0e+00 6.0e+07 1.2e+08
maploc
Chromosome HiC_scaffold_10
1.2e+08 1.4e+08 1.6e+08
maploc
Chromosome HiC_scaffold_11
1.7e+08 1.9e+08 2.1e+08
maploc
Chromosome HiC_scaffold_12
2.2e+08 2.4e+08
maploc
Chromosome HiC_scaffold_13
2.6e+08 2.8e+08
maploc
Chromosome HiC_scaffold_14
3.0e+08 3.2e+08
maploc
Chromosome HiC_scaffold_15
3.30e+08 3.45e+08 3.60e+08
maploc
Chromosome HiC_scaffold_16
3.65e+08 3.80e+08 3.95e+08
maploc
Chromosome HiC_scaffold_17
4.00e+08 4.15e+08 4.30e+08
maploc
Chromosome HiC_scaffold_18
4.35e+08 4.50e+08 4.65e+08
maploc
Chromosome HiC_scaffold_19
4.65e+08 4.80e+08
maploc
Chromosome HiC_scaffold_2
5.0e+08 5.6e+08
maploc
Chromosome HiC_scaffold_20
6.05e+08 6.20e+08
maploc
Chromosome HiC_scaffold_21
6.30e+08 6.45e+08
maploc
Chromosome HiC_scaffold_22
6.60e+08 6.75e+08
maploc
Chromosome HiC_scaffold_23
6.90e+08 7.05e+08
maploc
Chromosome HiC_scaffold_24
7.15e+08 7.30e+08
maploc
Chromosome HiC_scaffold_25
7.45e+08 7.60e+08
maploc
Chromosome HiC_scaffold_26
7.70e+08 7.85e+08
maploc
Chromosome HiC_scaffold_27
7.95e+08 8.10e+08
maploc
Chromosome HiC_scaffold_28
8.20e+08 8.30e+08
maploc
Chromosome HiC_scaffold_29
8.40e+08 8.50e+08
maploc
Chromosome HiC_scaffold_3
8.6e+08 9.0e+08 9.4e+08
maploc
Chromosome HiC_scaffold_30
9.65e+08 9.75e+08
maploc
Chromosome HiC_scaffold_31
9.82e+08 9.88e+08 9.94e+08
maploc
Chromosome HiC_scaffold_32
9.940e+08 1.000e+09 1.006e+09
maploc
Chromosome HiC_scaffold_33
1.006e+09 1.012e+09
maploc
Chromosome HiC_scaffold_34
1.018e+09 1.024e+09
maploc
Chromosome HiC_scaffold_35
1.028e+09 1.034e+09
maploc
Chromosome HiC_scaffold_36
1.040e+09 1.046e+09
maploc
Chromosome HiC_scaffold_37
1.052e+09 1.058e+09
maploc
Chromosome HiC_scaffold_38
1.060e+09 1.064e+09 1.068e+09
maploc
Chromosome HiC_scaffold_39
1.070e+09 1.074e+09
maploc
Chromosome HiC_scaffold_4
1.08e+09 1.14e+09
maploc
Chromosome HiC_scaffold_40
1.182e+09 1.186e+09
maploc
Chromosome HiC_scaffold_41
1.189e+09 1.193e+09
maploc
Chromosome HiC_scaffold_42
1.196e+09 1.199e+09 1.202e+09
maploc
Chromosome HiC_scaffold_43
1.203e+09 1.206e+09
maploc
Chromosome HiC_scaffold_44
1.208e+09 1.211e+09
maploc
Chromosome HiC_scaffold_45
1.213e+09 1.216e+09
maploc
Chromosome HiC_scaffold_46
1.218e+09 1.221e+09
maploc
Chromosome HiC_scaffold_47
1.224e+09 1.226e+09
maploc
Chromosome HiC_scaffold_48
1.227e+09 1.229e+09
maploc
Chromosome HiC_scaffold_49
1231000000 1233000000
maploc
Chromosome HiC_scaffold_5
1.24e+09 1.28e+09 1.32e+09
maploc
Chromosome HiC_scaffold_50
1322500000 1324500000
maploc
Chromosome HiC_scaffold_51
1325500000 1326500000
maploc
Chromosome HiC_scaffold_52
1328000000 1328200000
maploc
Chromosome HiC_scaffold_53
1328750000 1328900000
maploc
Chromosome HiC_scaffold_54
1329420000 1329450000
maploc
Chromosome HiC_scaffold_6
1.34e+09 1.38e+09
maploc
Chromosome HiC_scaffold_7
1.42e+09 1.45e+09 1.48e+09
maploc
Chromosome HiC_scaffold_8
1.48e+09 1.51e+09 1.54e+09
maploc
Chromosome HiC_scaffold_9
1.54e+09 1.57e+09
maploc
