# Supplementary material for: The sterlet sturgeon genome sequence and the mechanisms of segmental rediploidization
Source: Nat Ecol Evol. 2020 Mar 30;4(6):841–52. doi: 10.1038/s41559-020-1166-x (PMC7269910; doi:10.1038/s41559-020-1166-x)
Supplement: Supplementary file 2 — Reporting Summary [file 41559_2020_1166_MOESM2_ESM.pdf]

nature research

Corresponding author(s): Manfred Schartl, Matthias Stöck

Last updated by author(s): Feb 27, 2020

Reporting Summary

Nature Research wishes to improve the reproducibility of the work that we publish. This form provides structure for consistency and transparency in reporting. For further information on Nature Research policies, see Authors & Referees and the Editorial Policy Checklist.

Statistics

For all statistical analyses, confirm that the following items are present in the figure legend, table legend, main text, or Methods section.

n/a	Confirmed	
☒	☐	The exact sample size (*n*) for each experimental group/condition, given as a discrete number and unit of measurement
☒	☐	A statement on whether measurements were taken from distinct samples or whether the same sample was measured repeatedly
☒	☐	The statistical test(s) used AND whether they are one- or two-sided<br>*Only common tests should be described solely by name; describe more complex techniques in the Methods section.*
☒	☐	A description of all covariates tested
☒	☐	A description of any assumptions or corrections, such as tests of normality and adjustment for multiple comparisons
☒	☐	A full description of the statistical parameters including central tendency (e.g. means) or other basic estimates (e.g. regression coefficient) AND variation (e.g. standard deviation) or associated estimates of uncertainty (e.g. confidence intervals)
☒	☐	For null hypothesis testing, the test statistic (e.g. *F*, *t*, *r*) with confidence intervals, effect sizes, degrees of freedom and *P* value noted<br>*Give P values as exact values whenever suitable.*
☒	☐	For Bayesian analysis, information on the choice of priors and Markov chain Monte Carlo settings
☒	☐	For hierarchical and complex designs, identification of the appropriate level for tests and full reporting of outcomes
☒	☐	Estimates of effect sizes (e.g. Cohen's *d*, Pearson's *r*), indicating how they were calculated

Our web collection on statistics for biologists contains articles on many of the points above.

Software and code

Policy information about availability of computer code

Data collection	All commercial, open source and custom codes used to collect the data are described and referenced in the manuscript and are publicly available.
Data analysis	All commercial, open source and custom codes used to analyse the data are described and referenced in the manuscript and are publicly available.

For manuscripts utilizing custom algorithms or software that are central to the research but not yet described in published literature, software must be made available to editors/reviewers. We strongly encourage code deposition in a community repository (e.g. GitHub). See the Nature Research guidelines for submitting code & software for further information.

Data

Policy information about availability of data

All manuscripts must include a data availability statement. This statement should provide the following information, where applicable:

- Accession codes, unique identifiers, or web links for publicly available datasets
- A list of figures that have associated raw data
- A description of any restrictions on data availability

The Acipenser ruthenus Whole Genome Shotgun project has been deposited at DDBJ/ENA/GenBank under the accession VTUV00000000. The version described in this paper is version VTUV01000000. Genomic and transcriptomic reads are deposited in the sequence read archive under accession numbers SRR10188515-10188518 and SRR11013451-11013458.

Field-specific reporting

Please select the one below that is the best fit for your research. If you are not sure, read the appropriate sections before making your selection.

☒ Life sciences ☐ Behavioural & social sciences ☐ Ecological, evolutionary & environmental sciences

For a reference copy of the document with all sections, see nature.com/documents/nr-reporting-summary-flat.pdf

Life sciences study design

All studies must disclose on these points even when the disclosure is negative.

Sample size	One individual male was used for whole genome sequencing, RNA-seq was done on samples from RNAs were obtained from six adult males, one juvenile male and three adult females. RAD-tags were generated from 31 females and 30 males.
Data exclusions	No data were excluded from the analysis
Replication	n/a
Randomization	n/a
Blinding	n/a

Reporting for specific materials, systems and methods

We require information from authors about some types of materials, experimental systems and methods used in many studies. Here, indicate whether each material, system or method listed is relevant to your study. If you are not sure if a list item applies to your research, read the appropriate section before selecting a response.

Materials & experimental systems

n/a	Involved in the study	
☒	☐	Antibodies
☒	☐	Eukaryotic cell lines
☒	☐	Palaeontology
☐	☒	Animals and other organisms
☒	☐	Human research participants
☒	☐	Clinical data

Methods

n/a	Involved in the study	
☒	☐	ChIP-seq
☒	☐	Flow cytometry
☒	☐	MRI-based neuroimaging

Animals and other organisms

Policy information about studies involving animals; ARRIVE guidelines recommended for reporting animal research

Laboratory animals	Acipenser ruthenus, from the sterlet sturgeon population maintained at the Leibniz Institute of Freshwater Ecology and Inland fisheries (IGB), Berlin. This stock is derived from the Danube population of A. ruthenus.
Wild animals	n/a
Field-collected samples	n/a
Ethics oversight	The experiments were carried out in accordance with the European Directive 2010/63/EU and German national legislation (Animal protection law, TierSchG). All experimental protocols that are part of this study were approved through an authorization (File # ZH 114, issued 06.02.2014) of the LAGeSo, Berlin, Germany.

Note that full information on the approval of the study protocol must also be provided in the manuscript.
